# Supplementary material for: Risk of dementia and Parkinson’s disease in patients treated with androgen deprivation therapy using gonadotropin-releasing hormone agonist for prostate cancer: A nationwide population-based cohort study
Source: PLoS One. 2020 Dec 30;15(12):e0244660. doi: 10.1371/journal.pone.0244660 (PMC7773184; doi:10.1371/journal.pone.0244660)
Supplement: S1 Table — (DOCX) [file pone.0244660.s001.docx]

**S1 Table. Codes used to identify diagnosis.**

| **Diagnosis** | **ICD-10 code** |
| --- | --- |
| **Dementia** | F00–F03, F05 G30, G31 |
| **Parkinson’s disease** | G20 |
| **Cerebrovascular attack** | I60–I69 |
| **Ischemic heart disease** | I20I25 |
| **Myocardial infarction** | I21 |
| **Diabetes mellitus** | E11–E14 |
| **Hypertension** | I10–I15 |
| **Hyperlipidaemia** | E78 |
| **Liver disease** |  |
| Mild | K70, K71, K73, K75 |
| Moderate to severe | K72, K74 |
| **Other cancer** | All C codes except C61 |
| **Chronic kidney disease** | N1–N19 |
| **Chronic obstructive pulmonary disease** | J41, J43, J44, J47 |
| **Asthma** | J45, J46 |
| **Congestive heart failure** | I50 |
| **Peripheral vascular disease** | I70–I89 |
| **Connective tissue disease** | M30–M36 |
| **Peptic ulcer disease** | K25–K28 |
| **Hemiplegia** | G810, G811, G819 |
| **Acquired immune deficiency syndrome, AIDS** | B20–B24 |
