# Supplementary material for: Risk of dementia and Parkinson’s disease in patients treated with androgen deprivation therapy using gonadotropin-releasing hormone agonist for prostate cancer: A nationwide population-based cohort study
Source: PLoS One. 2020 Dec 30;15(12):e0244660. doi: 10.1371/journal.pone.0244660 (PMC7773184; doi:10.1371/journal.pone.0244660)
Supplement: S2 Table — (DOCX) [file pone.0244660.s002.docx]

**S2 Table. Codes used to identify medications.**

| **Medication** | **NHI main component code** |
| --- | --- |
| **Gonadotropin-releasing hormone agonist (GnRHa)** |  |
| Triptorelin | 244902BIJ, 244930BIJ, 467501BIJ |
| Leuprolide | 182602BIJ, 182604BIJ, |
|  | 182605BIJ-182610BIJ, 182630BIJ |
| Goserelin | 167201BIJ, 167202BIJ |
| **Statin** | 111501ATB-111504ATB, 136901ATB, |
|  | 162401ACH, 162402ACH, 162403ATR, |
|  | 165001ACH, 185801ATB, 194930ATB, |
|  | 216601ATB-216604ATB, 227801ATB, |
|  | 227801ATR, 227802ATB, 240301ATB, |
|  | 454001ATB-454003ATB, |
|  | 470901ATB-470903ATB, 471000ATB, |
|  | 471100ATB, 502201ATB-502204ATB, |
|  | 507800ATB, 519300ACH, 520301ACH, |
|  | 553700ATB, 631400ATB, 631500ATB, |
|  | 633800ATB, 633900ATB, 634600ATB, |
|  | 634800ATB, 634900ATB, 635000ATB, |
|  | 635100ATB, 635200ATB, 640700ATB, |
|  | 640800ATB, 640900ATB |
| **Antihypertensive** | 107601ATB, 107601ATD, 107602ATB, |
|  | 107602ATD, 110201ATB, 110202ATB, |
|  | 111401ATB-111403ATB, |
|  | 114001ACH-114003ACH, 115102ATB, |
|  | 115103ATB, 117901ATB-117904ATB, |
|  | 122601ATB-122604ATB, |
|  | 122901ATB-122903ATB, |
|  | 125001ATB-125003ATB, 125004ACR, |
|  | 125005ATB, 125006ACR-125008ACR, |
|  | 133001ATB-133003ATB, 133101ATB, |
|  | 133102ATB, 145703ACR, 145706ATB, |
|  | 145707ATR, 151601ATB-151603ATB, |
|  | 163501ATB, 163502ATB, 170701ATB, |
|  | 177301ATB, 177303ATB, 178403ACR, |
|  | 178407ATR, 178432CSI, 178501ATB, |
|  | 178504ATR, 180301ATB-180303ATB, |
|  | 182001ATB, 182002ATB, 185701ATB, |
|  | 185702ATB, 188001ATB-188003ATB, |
|  | 191502ATB, 191502ATR, 193802ATB, |
|  | 196102ATB, 167001ATB, 167002ATB, |
|  | 201301ATB-201303ATB, 201407ACS, |
|  | 201409ATR, 202506CPC, 202511COM, |
|  | 202601ATL, 202602ATL, 202605COM, |
|  | 202634CPC, 202636COM, 211301ATB, |
|  | 211302ATB, 219901ATB, 219904ATB, |
|  | 222401ATB, 222402ATB, 222404ATB, |
|  | 235002ATB, 244501ATB, 244502ATR, |
|  | 247101ATB-247104ATB, 249610CSI, |
|  | 262500ATB, 356400ATB, 378801ATB, |
|  | 378802ATB, 378900ATB, 385700ATB, |
|  | 385800ATB, 423700ATB, 429201ATB, |
|  | 441201ATB, 441202ATB, 442600ATB, |
|  | 443200ATB, 443300ATB, 447100ATB, |
|  | 447200ATB, 448600ATB, 448700ATB, |
|  | 459901ATB, 460500ATB, |
|  | 468501ATB-468503ATB, 486900ATB, |
|  | 489501ATB-489503ATB, 492800ATB, |
|  | 492900ATB, 495800ATB, 500500ATB, |
|  | 500600ATB, 501601ATB, 501602ATB, |
|  | 501801ATB, 502600ATB, 502700ATB, |
|  | 503000ATB, 509200ATB, |
|  | 510401ATB-510403ATB, 511500ATB, |
|  | 511600ATB, 511700ATB, 513600ATB, |
|  | 513900ATB, 515201ATB-515203ATB, |
|  | 519700ATB, 519800ATB, 519900ATB, |
|  | 520000ATB, 520100ATB, 522000ATB, |
|  | 522200ATB, 522300ATB, 522400ATB, |
|  | 526800ATB, 528201ATR, 528202ATR, |
|  | 553301ATB, 553800ATB, 556100ATB, |
|  | 556200ATB, 564701ATB, 564702ATB, |
|  | 582200ATB, 582400ATB, |
|  | 651401ATB-615403ATB, 651900ATB, |
|  | 652000ATB, 652100ATB, 652700ATB, |
|  | 652900ATB, 653000ATB, 653100ATB, |
|  | 662800ATB, 662900ATB, 663000ATB, |
|  | 663900ATB, 664000ATB, 664100ATB, |
|  | 664200ATB, 664300ATB, 664400ATB |
| **Anti-cancer drug** | 148301BIJ, 148302BIJ, 148310BIJ, |
|  | 148340BIJ-148342BIJ, |
|  | 148344BIJ-148346BIJ, |
|  | 148348BIJ-148351BIJ |
| **Anticoagulants** | 109301BIJ, 152101BIJ, 152130BIJ, |
|  | 152132BIJ-152134BIJ, 168501BIJ, |
|  | 168601BIJ-168603BIJ, 168606BIJ, |
|  | 168608BIJ, 168609CSI, 168610CSI, |
|  | 168630BIJ-168635BIJ, 249101ATB, |
|  | 249103ATB-249109ATB, |
|  | 511401ATB-511404ATB, |
|  | 613701ACH-613703ACH, |
|  | 643601ATB-643603ATB |
| **Antiplatelets** | 100430BIJ, 100431BIJ, 110701ATB, |
|  | 110701ATE, 110702ATB, 110704ATB, |
|  | 110705ACE, 110706ATB, 110801ATB, |
|  | 110802ATB, 110902BIJ, 111001ACE, |
|  | 111001ATB, 111001ATE, 111002ATE |
|  | 111003ACE, 111003ATE, 133201ACR, |
|  | 133201ATB, 133201ATD, 133202APD, |
|  | 133202ATB, 133202ATD, 133203ACR, |
|  | 133203ATR, 136901ATB, 244101ACH, |
|  | 498801ATB, 506100ATB, 517900ACE, |
|  | 517900ATE, 597301ATB, 597302ATB, |
|  | 617001ATB, 617002ATB |
