# Supplementary material for: Risk of dementia and Parkinson’s disease in patients treated with androgen deprivation therapy using gonadotropin-releasing hormone agonist for prostate cancer: A nationwide population-based cohort study
Source: PLoS One. 2020 Dec 30;15(12):e0244660. doi: 10.1371/journal.pone.0244660 (PMC7773184; doi:10.1371/journal.pone.0244660)
Supplement: S3 Table — (DOCX) [file pone.0244660.s003.docx]

**S3 Table. Distribution of baseline characteristics across GnRHa users or nonusers before and after the propensity score adjustment in the hospital validation cohort.**

|  | **N (%)** | |  | ***P*** | |
| --- | --- | --- | --- | --- | --- |
| **Characteristics** | **GnRHa Users**  **N = 205** | **GnRHa Nonusers**  **N = 479** |  | **Before adjustment** | **After propensity adjustment** |
| **Age at diagnosis, year** |  |  |  | 0.0019 | 0.5428 |
| <55 | 8 (3.8) | 51 (10.7) |  |  |  |
| 55-64 | 25 (12.4) | 115 (24.1) |  |  |  |
| 65-74 | 98 (47.8) | 198 (41.3) |  |  |  |
| ≥75 | 74 (36.0) | 115 (23.9) |  |  |  |
| **Residence** |  |  |  | 0.0026 | 0.7514 |
| Urban | 175 (85.2) | 425 (88.8) |  |  |  |
| Suburban/rural | 30 (14.8) | 54 (11.2) |  |  |  |
| **Insurance type** |  |  |  | 0.0612 | 0.7021 |
| National health insurance | 195 (95.2) | 458 (95.6) |  |  |  |
| Medicare | 10 (4.8) | 21 (4.4) |  |  |  |
| **Alcohol consumption** |  |  |  | 0.0074 | 0.5746 |
| None | 30 (14.5) | 80 (16.8) |  |  |  |
| Light | 35 (16.9) | 225 (47.0) |  |  |  |
| Moderate | 107 (52.2) | 147 (30.7) |  |  |  |
| Heavy | 33 (16.4) | 27 (5.5) |  |  |  |
| **Smoking status (pack years)** |  |  |  | <0.0001 | 0.3985 |
| Never smokers | 67 (32.7) | 241 (50.4) |  |  |  |
| Ex-smokers | 16 (7.8) | 51 (10.6) |  |  |  |
| < 30 | 31 (14.9) | 76 (15.9) |  |  |  |
| 30-59 | 51 (25.0) | 80 (16.7) |  |  |  |
| ≥ 60 | 40 (19.6) | 31 (6.4) |  |  |  |
| **Body mass index (kg/m^2^)** |  |  |  | 0.5384 | 0.8942 |
| < 18.5 | 7 (3.5) | 11 (2.3) |  |  |  |
| 18.5-22.9 | 65 (31.8) | 154 (32.1) |  |  |  |
| 20.3-24.9 | 84 (41.2) | 195 (40.8) |  |  |  |
| ≥ 25 | 49 (23.5) | 119 (24.8) |  |  |  |
| **Prostate-specific antigen (ng/mL)** |  |  |  | <0.0001 |  |
| < 30 | 104 (50.6) | 385 (80.4) |  |  |  |
| ≥ 30 | 101 (49.4) | 94 (19.6) |  |  |  |
| **Biopsy Gleason score** |  |  |  | <0.0001 | 0.2648 |
| ≤6 | 17 (8.3) | 215 (44.9) |  |  |  |
| 7 | 72 (35.1) | 161 (33.7) |  |  |  |
| ≥8 | 116 (56.6) | 103 (21.4) |  |  |  |
| **Clinical T stage** |  |  |  | <0.0001 | 0.3514 |
| T1 | 15 (7.4) | 92 (19.3) |  |  |  |
| T2 | 72 (34.9) | 335 (69.9) |  |  |  |
| T3, T4 | 118 (57.7) | 52 (10.8) |  |  |  |
| **Prior medication use** |  |  |  |  |  |
| Statin | 32 (15.7) | 88 (18.4) |  | 0.0215 | 0.7581 |
| Antihypertensive | 41 (19.8) | 85 (17.7) |  | 0.1951 | 0.8425 |
| Anticoagulants | 30 (14.5) | 78 (16.3) |  | 0.0842 | 0.4527 |
| Antiplatelet therapy | 15 (7.2) | 36 (7.6) |  | 0.2855 | 0.6564 |
| **Prior antiandrogen use** | 43 (20.9) | 6 (1.2) |  | <0.0001 |  |
| **Medical history** |  |  |  |  |  |
| Hypertension | 108 (52.8) | 146 (30.4) |  | <0.0001 | 0.2154 |
| Diabetes mellitus | 62 (30.4) | 88 (18.4) |  | 0.0032 | 0.5417 |
| Hyperlipidaemia | 40 (19.4) | 79 (16.5) |  | 0.0197 | 0.3842 |
| Cardiovascular disease | 51 (24.8) | 65 (13.6) |  | 0.0024 | 0.7514 |
| Liver disease | 3 (1.5) | 9 (1.9) |  | 0.2684 | 0.8724 |
| Other cancer | 10 (4.8) | 15 (3.2) |  | 0.6285 | 0.3576 |
| Chronic kidney disease | 6 (2.8) | 10 (2.1) |  | 0.3858 | 0.4541 |
| COPD | 15 (7.5) | 28 (5.9) |  | 0.0598 | 0.7452 |
| Asthma | 8 (3.7) | 23 (4.8) |  | 0.1423 | 0.5424 |
| Peripheral vascular disease | 6 (2.8) | 7 (1.5) |  | 0.2157 | 0.3564 |
| **Charlson comorbidity index** |  |  |  | 0.0008 | 0.2549 |
| 0, 1 | 11 (5.2) | 45 (9.3) |  |  |  |
| 2 | 20 (9.6) | 113 (23.5) |  |  |  |
| 3 | 59 (28.9) | 109 (22.8) |  |  |  |
| 4 | 65 (31.5) | 136 (28.3) |  |  |  |
| ≥5 | 50 (24.8) | 76 (16.1) |  |  |  |
| **Other treatment** |  |  |  | <0.0001 |  |
| Radical prostatectomy | 40 (19.5) | 300 (62.7) |  |  |  |
| Radiotherapy | 55 (26.8) | 114 (23.8) |  |  |  |
