# Supplementary material for: Risk of dementia and Parkinson’s disease in patients treated with androgen deprivation therapy using gonadotropin-releasing hormone agonist for prostate cancer: A nationwide population-based cohort study
Source: PLoS One. 2020 Dec 30;15(12):e0244660. doi: 10.1371/journal.pone.0244660 (PMC7773184; doi:10.1371/journal.pone.0244660)
Supplement: S1 Data — (DOCX) [file pone.0244660.s004.docx]

LIBNAME SHIM '/vol/userdata3/sta_room090'

/*

DATA SHIM.MIDNAL(KEEP=MID NAL);

SET SHIM.T530_2018Q1_09;

NAL=SUBSTR(PRSCP_GRANT_NO,1,8);

RUN;

PROC SORT DATA=SHIM.MIDNAL OUT=SHIM.MIDNAL_S NODUPKEY; BY MID NAL; RUN;

DATA SHIM.MIDDRUG;

SET SHIM.T300_2018Q1_09;

IF GNL_CD='244902BIJ' OR

GNL_CD='467501BIJ' OR

GNL_CD='182604BIJ' OR

GNL_CD='182602BIJ' OR

GNL_CD='167202BIJ' OR

GNL_CD='167201BIJ' OR

GNL_CD='182610BIJ' OR

GNL_CD='182609BIJ' OR

GNL_CD='182608BIJ' OR

GNL_CD='182607BIJ' OR

GNL_CD='182606BIJ' OR

GNL_CD='182605BIJ' THEN DRUG=1;

ELSE DRUG=0;

RUN;

DATA SHIM.MIDDRUG_1(KEEP=MID GNL_CD);

SET SHIM.MIDDRUG;

IF DRUG=1;

RUN;

DATA SHIM.MIDDRUG_2;

SET SHIM.MIDDRUG_1;

IF GNL_CD='244902BIJ' OR

GNL_CD='182602BIJ' OR

GNL_CD='182609BIJ' OR

GNL_CD='182605BIJ' OR

GNL_CD='167202BIJ' THEN ADT_1=1; ELSE ADT_1=0;

IF GNL_CD='467501BIJ' OR

GNL_CD='182604BIJ' OR

GNL_CD='182606BIJ' OR

GNL_CD='167201BIJ' THEN ADT_3=1; ELSE ADT_3=0;

IF GNL_CD='182607BIJ' THEN ADT_4=1; ELSE ADT_4=0;

IF GNL_CD='182610BIJ' OR

GNL_CD='182608BIJ' THEN ADT_6=1; ELSE ADT_6=0;

RUN;

DATA SHIM.MIDMAIN;

SET SHIM.T200_2018Q1_09;

KEEP MID JID PAT_AGE CL_CD RVD_PLC_CD MAIN_SICK SUB_SICK RECU_FR_DD;

RUN;

DATA SHIM.MIDMAIN_1;

SET SHIM.MIDMAIN;

SICK_1=SUBSTR(MAIN_SICK,1,3);

SICK_2=SUBSTR(MAIN_SICK,1,4);

SICK_3=SUBSTR(SUB_SICK,1,3);

SICK_4=SUBSTR(SUB_SICK,1,4);

RUN;

DATA SHIM.MIDMAIN_2;

SET SHIM.MIDMAIN_1;

IF 'E11'<=SICK_1<='E14' THEN DM_1=1; ELSE DM_1=0;

IF 'I10'<=SICK_1<='I15' THEN HTN_1=1; ELSE HTN_1=0;

IF 'I20'<=SICK_1<='I25' THEN IHD_1=1; ELSE IHD_1=0;

IF 'I60'<=SICK_1<='I69' THEN CVA_1=1; ELSE CVA_1=0;

IF 'G20'<=SICK_1<='G22' THEN PKS_1=1; ELSE PKS_1=0;

IF 'E11'<=SICK_3<='E14' THEN DM_2=1; ELSE DM_2=0;

IF 'I10'<=SICK_3<='I15' THEN HTN_2=1; ELSE HTN_2=0;

IF 'I20'<=SICK_3<='I25' THEN IHD_2=1; ELSE IHD_2=0;

IF 'I60'<=SICK_3<='I69' THEN CVA_2=1; ELSE CVA_2=0;

IF 'G20'<=SICK_3<='G22' THEN PKS_2=1; ELSE PKS_2=0;

IF DM_1=1 OR DM_2=1 THEN DM=1; ELSE DM=0;

IF HTN_1=1 OR HTN_2=1 THEN HTN=1; ELSE HTN=0;

IF IHD_1=1 OR IHD_2=1 THEN IHD=1; ELSE IHD=0;

IF CVA_1=1 OR CVA_2=1 THEN CVA=1; ELSE CVA=0;

IF PKS_1=1 OR PKS_2=1 THEN PKS=1; ELSE PKS=0;

IF SICK_2='F000' THEN ALZ_1=1;

ELSE IF SICK_2='F001' THEN ALZ_1=1;

ELSE IF SICK_2='F002' THEN ALZ_1=1;

ELSE IF SICK_2='F009' THEN ALZ_1=1;

ELSE IF SICK_2='G300' THEN ALZ_1=1;

ELSE IF SICK_2='G301' THEN ALZ_1=1;

ELSE IF SICK_2='G308' THEN ALZ_1=1;

ELSE IF SICK_2='G309' THEN ALZ_1=1;

ELSE ALZ_1=0;

IF SICK_4='F000' THEN ALZ_2=1;

ELSE IF SICK_4='F001' THEN ALZ_2=1;

ELSE IF SICK_4='F002' THEN ALZ_2=1;

ELSE IF SICK_4='F009' THEN ALZ_2=1;

ELSE IF SICK_4='G300' THEN ALZ_2=1;

ELSE IF SICK_4='G301' THEN ALZ_2=1;

ELSE IF SICK_4='G308' THEN ALZ_2=1;

ELSE IF SICK_4='G309' THEN ALZ_2=1;

ELSE ALZ_2=0;

IF ALZ_1=1 OR ALZ_2=1 THEN ALZ=1; ELSE ALZ=0;

IF SICK_1='K75' OR SICK_1='K73' OR SICK_1='K70' OR SICK_1='K71' THEN MLIV_1=1; ELSE MLIV_1=0;

IF SICK_3='K75' OR SICK_3='K73' OR SICK_3='K70' OR SICK_3='K71' THEN MLIV_2=1; ELSE MLIV_2=0;

IF MLIV_1=1 OR MLIV_2=1 THEN MLIV=1; ELSE MLIV=0;

IF SICK_1='K72' OR SICK_1='K74' THEN SLIV_1=1; ELSE SLIV_1=0;

IF SICK_3='K72' OR SICK_3='K74' THEN SLIV_2=1; ELSE SLIV_2=0;

IF SLIV_1=1 OR SLIV_2=1 THEN SLIV=1; ELSE SLIV=0;

IF 'C00'<=SICK_1<='C99' THEN CAN_1=1; ELSE CAN_1=0;

IF 'C00'<=SICK_3<='C99' THEN CAN_2=1; ELSE CAN_2=0;

IF CAN_1=1 OR CAN_2=1 THEN CAN=1; ELSE CAN=0;

IF SICK_1='C61' OR SICK_3='C61' THEN CAN=0;

IF 'B20'<=SICK_1<='B24' THEN AIDS_1=1; ELSE AIDS_1=0;

IF 'B20'<=SICK_3<='B24' THEN AIDS_2=1; ELSE AIDS_2=0;

IF AIDS_1=1 OR AIDS_2=1 THEN AIDS=1; ELSE AIDS=0;

IF 'N17'<=SICK_1<='N19' THEN CKD_1=1; ELSE CKD_1=0;

IF 'N17'<=SICK_3<='N19' THEN CKD_2=1; ELSE CKD_2=0;

IF CKD_1=1 OR CKD_2=1 THEN CKD=1; ELSE CKD=0;

IF SICK_1='N50' THEN CHF_1=1; ELSE CHF_1=0;

IF SICK_3='N50' THEN CHF_2=1; ELSE CHF_2=0;

IF CHF_1=1 OR CHF_2=1 THEN CHF=1; ELSE CHF=0;

IF SICK_1='I21' THEN MI_1=1; ELSE MI_1=0;

IF SICK_3='I21' THEN MI_2=1; ELSE MI_2=0;

IF MI_1=1 OR MI_2=1 THEN MI=1; ELSE MI=0;

IF SICK_1='J41' OR SICK_1='J43' OR SICK_1='J44' OR SICK_1='J47' THEN COPD_1=1; ELSE COPD_1=0;

IF SICK_3='J41' OR SICK_3='J43' OR SICK_3='J44' OR SICK_3='J47' THEN COPD_2=1; ELSE COPD_2=0;

IF COPD_1=1 OR COPD_2=1 THEN COPD=1; ELSE COPD=0;

IF 'J45'<=SICK_1<='J46' THEN ASTH_1=1; ELSE ASTH_1=0;

IF 'J45'<=SICK_3<='J46' THEN ASTH_2=1; ELSE ASTH_2=0;

IF ASTH_1=1 OR ASTH_2=1 THEN ASTH=1; ELSE ASTH=0;

IF 'I70'<=SICK_1<='I89' THEN PVD_1=1; ELSE PVD_1=0;

IF 'I70'<=SICK_3<='I89' THEN PVD_2=1; ELSE PVD_2=0;

IF PVD_1=1 OR PVD_2=1 THEN PVD=1; ELSE PVD=0;

IF SICK_1='G81' THEN HEMI_1=1; ELSE HEMI_1=0;

IF SICK_3='G81' THEN HEMI_2=1; ELSE HEMI_2=0;

IF HEMI_1=1 OR HEMI_2=1 THEN HEMI=1; ELSE HEMI=0;

IF 'M30'<=SICK_1<='M36' THEN CONN_1=1; ELSE CONN_1=0;

IF 'M30'<=SICK_3<='M36' THEN CONN_2=1; ELSE CONN_2=0;

IF CONN_1=1 OR CONN_2=1 THEN CONN=1; ELSE CONN=0;

IF 'K25'<=SICK_1<='K28' THEN PUD_1=1; ELSE PUD_1=0;

IF 'K25'<=SICK_3<='K28' THEN PUD_2=1; ELSE PUD_2=0;

IF PUD_1=1 OR PUD_2=1 THEN PUD=1; ELSE PUD=0;

IF 'I60'<=SICK_1<='I62' THEN HCVA_1=1; ELSE HCVA_1=0;

IF 'I60'<=SICK_3<='I62' THEN HCVA_2=1; ELSE HCVA_2=0;

IF HCVA_1=1 OR HCVA_2=1 THEN HCVA=1; ELSE HCVA=0;

IF 'I63'<=SICK_1<='I66' THEN ICVA_1=1; ELSE ICVA_1=0;

IF 'I63'<=SICK_3<='I66' THEN ICVA_2=1; ELSE ICVA_2=0;

IF ICVA_1=1 OR ICVA_2=1 THEN ICVA=1; ELSE ICVA=0;

RUN;

*==================================;

*==================================;

DATA SHIM.MIDMAIN_3;

SET SHIM.MIDMAIN_2;

KEEP MID JID PAT_AGE CL_CD RVD_PLC_CD RECU_FR_DD DM HTN IHD CVA PKS ALZ MLIV SLIV CAN AIDS CKD CHF MI COPD ASTH PVD HEMI CONN PUD;

RUN;

PROC SORT DATA=SHIM.MIDDRUG_2 OUT=SHIM.MIDDRUG_S; BY MID; RUN;

PROC SORT DATA=SHIM.MIDMAIN_3 OUT=SHIM.MIDMAIN_S; BY MID; RUN;

DATA SHIM.TOTAL;

MERGE SHIM.MIDDRUG_S SHIM.MIDMAIN_S;

BY MID;

RUN;

DATA SHIM.TOTAL_1;

SET SHIM.TOTAL;

IF ADT_1=. THEN ADT_1=0;

IF ADT_3=. THEN ADT_3=0;

IF ADT_4=. THEN ADT_4=0;

IF ADT_6=. THEN ADT_6=0;

RUN;

DATA SHIM.TYPE;

SET SHIM.T200_2018Q1_09;

KEEP MID INSUP_TP_CD;

RUN;

PROC SORT DATA=SHIM.TYPE OUT=SHIM.TYPE_S; BY MID; RUN;

DATA SHIM.MIDMX(KEEP=MID MX);

SET SHIM.T300_2018Q1_09;

IF GNL_CD='148301BIJ' OR

GNL_CD='148302BIJ' OR

GNL_CD='148310BIJ' OR

GNL_CD='148340BIJ' OR

GNL_CD='148341BIJ' OR

GNL_CD='148342BIJ' OR

GNL_CD='148344BIJ' OR

GNL_CD='148345BIJ' OR

GNL_CD='148346BIJ' OR

GNL_CD='148348BIJ' OR

GNL_CD='148350BIJ' OR

GNL_CD='148351BIJ' THEN MX=1;

ELSE IF GNL_CD='111501ATB' OR

GNL_CD='111502ATB' OR

GNL_CD='111503ATB' OR

GNL_CD='111504ATB' OR

GNL_CD='136901ATB' OR

GNL_CD='162401ACH' OR

GNL_CD='162402ACH' OR

GNL_CD='162403ATR' OR

GNL_CD='165001ACH' OR

GNL_CD='185801ATB' OR

GNL_CD='194930ATB' OR

GNL_CD='216601ATB' OR

GNL_CD='216602ATB' OR

GNL_CD='216603ATB' OR

GNL_CD='216604ATB' OR

GNL_CD='227801ATB' OR

GNL_CD='227801ATR' OR

GNL_CD='227802ATB' OR

GNL_CD='240301ATB' OR

GNL_CD='454001ATB' OR

GNL_CD='454002ATB' OR

GNL_CD='454003ATB' OR

GNL_CD='470901ATB' OR

GNL_CD='470902ATB' OR

GNL_CD='470903ATB' OR

GNL_CD='471000ATB' OR

GNL_CD='471100ATB' OR

GNL_CD='502201ATB' OR

GNL_CD='502202ATB' OR

GNL_CD='502203ATB' OR

GNL_CD='502204ATB' OR

GNL_CD='507800ATB' OR

GNL_CD='519300ACH' OR

GNL_CD='520301ACH' OR

GNL_CD='553700ATB' OR

GNL_CD='631400ATB' OR

GNL_CD='631500ATB' OR

GNL_CD='633800ATB' OR

GNL_CD='633900ATB' OR

GNL_CD='634600ATB' OR

GNL_CD='634800ATB' OR

GNL_CD='634900ATB' OR

GNL_CD='635000ATB' OR

GNL_CD='635100ATB' OR

GNL_CD='635200ATB' OR

GNL_CD='640700ATB' OR

GNL_CD='640800ATB' OR

GNL_CD='640900ATB' THEN MX=2;

ELSE IF GNL_CD='107601ATB' OR

GNL_CD='107601ATD' OR

GNL_CD='107602ATB' OR

GNL_CD='107602ATD' OR

GNL_CD='110201ATB' OR

GNL_CD='110202ATB' OR

GNL_CD='111401ATB' OR

GNL_CD='111402ATB' OR

GNL_CD='111403ATB' OR

GNL_CD='114001ACH' OR

GNL_CD='114002ACH' OR

GNL_CD='114003ACH' OR

GNL_CD='115102ATB' OR

GNL_CD='115103ATB' OR

GNL_CD='117901ATB' OR

GNL_CD='117902ATB' OR

GNL_CD='117903ATB' OR

GNL_CD='117904ATB' OR

GNL_CD='122601ATB' OR

GNL_CD='122602ATB' OR

GNL_CD='122603ATB' OR

GNL_CD='122604ATB' OR

GNL_CD='122901ATB' OR

GNL_CD='122902ATB' OR

GNL_CD='122903ATB' OR

GNL_CD='125001ATB' OR

GNL_CD='125002ATB' OR

GNL_CD='125003ATB' OR

GNL_CD='125004ACR' OR

GNL_CD='125005ATB' OR

GNL_CD='125006ACR' OR

GNL_CD='125007ACR' OR

GNL_CD='125008ACR' OR

GNL_CD='133001ATB' OR

GNL_CD='133002ATB' OR

GNL_CD='133003ATB' OR

GNL_CD='133101ATB' OR

GNL_CD='133102ATB' OR

GNL_CD='145703ACR' OR

GNL_CD='145704BIJ' OR

GNL_CD='145706ATB' OR

GNL_CD='145707ATR' OR

GNL_CD='151601ATB' OR

GNL_CD='151602ATB' OR

GNL_CD='151603ATB' OR

GNL_CD='163501ATB' OR

GNL_CD='163502ATB' OR

GNL_CD='170701ATB' OR

GNL_CD='170730BIJ' OR

GNL_CD='177301ATB' OR

GNL_CD='177303ATB' OR

GNL_CD='178403ACR' OR

GNL_CD='178407ATR' OR

GNL_CD='178430BIJ' OR

GNL_CD='178431BIJ' OR

GNL_CD='178432CSI' OR

GNL_CD='178501ATB' OR

GNL_CD='178504ATR' OR

GNL_CD='180230BIJ' OR

GNL_CD='180231BIJ' OR

GNL_CD='180301ATB' OR

GNL_CD='180302ATB' OR

GNL_CD='180303ATB' OR

GNL_CD='182001ATB' OR

GNL_CD='182002ATB' OR

GNL_CD='185701ATB' OR

GNL_CD='185702ATB' OR

GNL_CD='188001ATB' OR

GNL_CD='188002ATB' OR

GNL_CD='188003ATB' OR

GNL_CD='191502ATB' OR

GNL_CD='191502ATR' OR

GNL_CD='193802ATB' OR

GNL_CD='196102ATB' OR

GNL_CD='197001ATB' OR

GNL_CD='197002ATB' OR

GNL_CD='201301ATB' OR

GNL_CD='201302BIJ' OR

GNL_CD='201303BIJ' OR

GNL_CD='201407ACS' OR

GNL_CD='201409ATR' OR

GNL_CD='202503BIJ' OR

GNL_CD='202506CPC' OR

GNL_CD='202511COM' OR

GNL_CD='202601ATL' OR

GNL_CD='202602ATL' OR

GNL_CD='202605COM' OR

GNL_CD='202632BIJ' OR

GNL_CD='202633BIJ' OR

GNL_CD='202634CPC' OR

GNL_CD='202635BIJ' OR

GNL_CD='202636COM' OR

GNL_CD='211301ATB' OR

GNL_CD='211302ATB' OR

GNL_CD='219901ATB' OR

GNL_CD='219904ATB' OR

GNL_CD='222401ATB' OR

GNL_CD='222402ATB' OR

GNL_CD='222404ATB' OR

GNL_CD='235002ATB' OR

GNL_CD='244501ATB' OR

GNL_CD='244502ATB' OR

GNL_CD='247101ATB' OR

GNL_CD='247102ATB' OR

GNL_CD='247103ATB' OR

GNL_CD='247104ATB' OR

GNL_CD='249610CSI' OR

GNL_CD='262500ATB' OR

GNL_CD='356400ATB' OR

GNL_CD='378801ATB' OR

GNL_CD='378802ATB' OR

GNL_CD='378900ATB' OR

GNL_CD='385700ATB' OR

GNL_CD='385800ATB' OR

GNL_CD='423700ATB' OR

GNL_CD='429201ATB' OR

GNL_CD='441201ATB' OR

GNL_CD='441202ATB' OR

GNL_CD='442600ATB' OR

GNL_CD='443200ATB' OR

GNL_CD='443300ATB' OR

GNL_CD='447100ATB' OR

GNL_CD='447200ATB' OR

GNL_CD='448600ATB' OR

GNL_CD='448700ATB' OR

GNL_CD='459901ATB' OR

GNL_CD='460500ATB' OR

GNL_CD='468501ATB' OR

GNL_CD='468502ATB' OR

GNL_CD='468503ATB' OR

GNL_CD='486900ATB' OR

GNL_CD='489501ATB' OR

GNL_CD='489502ATB' OR

GNL_CD='489503ATB' OR

GNL_CD='492800ATB' OR

GNL_CD='492900ATB' OR

GNL_CD='495800ATB' OR

GNL_CD='500500ATB' OR

GNL_CD='500600ATB' OR

GNL_CD='501601ATB' OR

GNL_CD='501602ATB' OR

GNL_CD='501801ATB' OR

GNL_CD='502600ATB' OR

GNL_CD='502700ATB' OR

GNL_CD='503000ATB' OR

GNL_CD='509200ATB' OR

GNL_CD='510401ATB' OR

GNL_CD='510402ATB' OR

GNL_CD='510403ATB' OR

GNL_CD='511500ATB' OR

GNL_CD='511600ATB' OR

GNL_CD='511700ATB' OR

GNL_CD='513600ATB' OR

GNL_CD='513900ATB' OR

GNL_CD='515201ATB' OR

GNL_CD='515202ATB' OR

GNL_CD='515203ATB' OR

GNL_CD='519700ATB' OR

GNL_CD='519800ATB' OR

GNL_CD='519900ATB' OR

GNL_CD='520000ATB' OR

GNL_CD='520100ATB' OR

GNL_CD='522000ATB' OR

GNL_CD='522200ATB' OR

GNL_CD='522300ATB' OR

GNL_CD='522400ATB' OR

GNL_CD='526800ATB' OR

GNL_CD='528201ATR' OR

GNL_CD='528202ATR' OR

GNL_CD='553301ATB' OR

GNL_CD='553800ATB' OR

GNL_CD='556100ATB' OR

GNL_CD='556200ATB' OR

GNL_CD='564701ATB' OR

GNL_CD='564702ATB' OR

GNL_CD='582200ATB' OR

GNL_CD='582400ATB' OR

GNL_CD='651401ATB' OR

GNL_CD='651402ATB' OR

GNL_CD='651403ATB' OR

GNL_CD='651900ATB' OR

GNL_CD='652000ATB' OR

GNL_CD='652100ATB' OR

GNL_CD='652700ATB' OR

GNL_CD='652900ATB' OR

GNL_CD='653000ATB' OR

GNL_CD='653100ATB' OR

GNL_CD='662800ATB' OR

GNL_CD='662900ATB' OR

GNL_CD='663000ATB' OR

GNL_CD='663900ATB' OR

GNL_CD='664000ATB' OR

GNL_CD='664100ATB' OR

GNL_CD='664200ATB' OR

GNL_CD='664300ATB' OR

GNL_CD='664400ATB' THEN MX=3;

ELSE IF GNL_CD='109301BIJ' OR

GNL_CD='152101BIJ' OR

GNL_CD='152130BIJ' OR

GNL_CD='152132BIJ' OR

GNL_CD='152133BIJ' OR

GNL_CD='152134BIJ' OR

GNL_CD='168501BIJ' OR

GNL_CD='168601BIJ' OR

GNL_CD='168602BIJ' OR

GNL_CD='168603BIJ' OR

GNL_CD='168606BIJ' OR

GNL_CD='168608BIJ' OR

GNL_CD='168609CSI' OR

GNL_CD='168610CSI' OR

GNL_CD='168630BIJ' OR

GNL_CD='168631BIJ' OR

GNL_CD='168632BIJ' OR

GNL_CD='168633BIJ' OR

GNL_CD='168634BIJ' OR

GNL_CD='168635BIJ' OR

GNL_CD='249101ATB' OR

GNL_CD='249103ATB' OR

GNL_CD='249104ATB' OR

GNL_CD='249105ATB' OR

GNL_CD='249106ATB' OR

GNL_CD='249107ATB' OR

GNL_CD='249108ATB' OR

GNL_CD='249109ATB' OR

GNL_CD='511401ATB' OR

GNL_CD='511402ATB' OR

GNL_CD='511403ATB' OR

GNL_CD='511404ATB' OR

GNL_CD='613701ACH' OR

GNL_CD='613702ACH' OR

GNL_CD='613703ACH' OR

GNL_CD='643601ATB' OR

GNL_CD='643602ATB' OR

GNL_CD='643603ATB' THEN MX=4;

ELSE IF GNL_CD='100430BIJ' OR

GNL_CD='100431BIJ' OR

GNL_CD='110701ATB' OR

GNL_CD='110701ATE' OR

GNL_CD='110702ATB' OR

GNL_CD='110704ATB' OR

GNL_CD='110705ACE' OR

GNL_CD='110706ATB' OR

GNL_CD='110801ATB' OR

GNL_CD='110802ATB' OR

GNL_CD='110902BIJ' OR

GNL_CD='111001ACE' OR

GNL_CD='111001ATB' OR

GNL_CD='111001ATE' OR

GNL_CD='111002ATE' OR

GNL_CD='111003ACE' OR

GNL_CD='111003ATE' OR

GNL_CD='133201ACR' OR

GNL_CD='133201ATB' OR

GNL_CD='133201ATD' OR

GNL_CD='133202APD' OR

GNL_CD='133202ATB' OR

GNL_CD='133202ATD' OR

GNL_CD='133203ACR' OR

GNL_CD='133203ATR' OR

GNL_CD='244101ACH' OR

GNL_CD='498801ATB' OR

GNL_CD='506100ATB' OR

GNL_CD='517900ACE' OR

GNL_CD='517900ATE' OR

GNL_CD='597301ATB' OR

GNL_CD='597302ATB' OR

GNL_CD='617001ATB' OR

GNL_CD='617002ATB' THEN MX=5;

ELSE IF GNL_CD='117201ATB' OR

GNL_CD='139401ATB' THEN MX=6;

ELSE MX=0;

RUN;

PROC SORT DATA=SHIM.MIDMX OUT=SHIM.MIDMX_S; BY MID; RUN;

DATA SHIM.MIDMX_S_2;

SET SHIM.MIDMX_S;

IF MX=1 THEN MX_CAN=1; ELSE MX_CAN=0;

IF MX=2 THEN MX_CHOL=1; ELSE MX_CHOL=0;

IF MX=3 THEN MX_HTN=1; ELSE MX_HTN=0;

IF MX=4 THEN MX_COA=1; ELSE MX_COA=0;

IF MX=5 THEN MX_PLT=1; ELSE MX_PLT=0;

IF MX=6 THEN MX_ANT=1; ELSE MX_ANT=0;

RUN;

PROC SQL;

CREATE TABLE SHIM.MIDMX_CAN AS

SELECT MID,

SUM(MX_CAN) AS CAN_SUM

FROM SHIM.MIDMX_S_2

GROUP BY MID;

QUIT;

RUN;

PROC SQL;

CREATE TABLE SHIM.MIDMX_CHOL AS

SELECT MID,

SUM(MX_CHOL) AS CHOL_SUM

FROM SHIM.MIDMX_S_2

GROUP BY MID;

QUIT;

RUN;

PROC SQL;

CREATE TABLE SHIM.MIDMX_HTN AS

SELECT MID,

SUM(MX_HTN) AS HTN_SUM

FROM SHIM.MIDMX_S_2

GROUP BY MID;

QUIT;

RUN;

PROC SQL;

CREATE TABLE SHIM.MIDMX_COA AS

SELECT MID,

SUM(MX_COA) AS COA_SUM

FROM SHIM.MIDMX_S_2

GROUP BY MID;

QUIT;

RUN;

PROC SQL;

CREATE TABLE SHIM.MIDMX_PLT AS

SELECT MID,

SUM(MX_PLT) AS PLT_SUM

FROM SHIM.MIDMX_S_2

GROUP BY MID;

QUIT;

PROC SQL;

CREATE TABLE SHIM.MIDMX_ANT AS

SELECT MID,

SUM(MX_ANT) AS ANT_SUM

FROM SHIM.MIDMX_S_2

GROUP BY MID;

QUIT;

RUN;

DATA SHIM.MID_SUM;

MERGE SHIM.MIDMX_CAN SHIM.MIDMX_CHOL SHIM.MIDMX_HTN SHIM.MIDMX_COA SHIM.MIDMX_PLT SHIM.MIDMX_ANT;

BY MID;

RUN;

DATA SHIM.MID_SUM_1(KEEP=MID TX_CAN TX_CHOL TX_HTN TX_COA TX_PLT TX_ANT);

SET SHIM.MID_SUM;

IF CAN_SUM>0 THEN TX_CAN=1; ELSE TX_CAN=0;

IF CHOL_SUM>0 THEN TX_CHOL=1; ELSE TX_CHOL=0;

IF HTN_SUM>0 THEN TX_HTN=1; ELSE TX_HTN=0;

IF COA_SUM>0 THEN TX_COA=1; ELSE TX_COA=0;

IF PLT_SUM>0 THEN TX_PLT=1; ELSE TX_PLT=0;

IF ANT_SUM>0 THEN TX_ANT=1; ELSE TX_ANT=0;

RUN;

*/

*=====================================;

*=====================================;

/*

DATA IMSI;

INPUT MID MX_CAN MX_CHOL MX_HTN MX_COA MX_PLT;

CARDS;

1 0 0 1 0 0

1 0 0 1 0 0

1 0 0 1 0 0

1 0 0 0 0 1

3 1 0 0 0 0

3 0 1 0 0 0

3 0 0 1 0 0

4 0 0 0 1 0

4 0 0 0 1 0

RUN;

PROC SQL;

CREATE TABLE IMSI_1 AS

SELECT MID,

SUM(MX_CAN) AS CAN_SUM

FROM IMSI

GROUP BY MID;

QUIT;

RUN;

PROC SQL;

CREATE TABLE IMSI_2 AS

SELECT MID,

SUM(MX_CHOL) AS CHOL_SUM

FROM IMSI

GROUP BY MID;

QUIT;

RUN;

PROC SQL;

CREATE TABLE IMSI_3 AS

SELECT MID,

SUM(MX_HTN) AS HTN_SUM

FROM IMSI

GROUP BY MID;

QUIT;

RUN;

PROC SQL;

CREATE TABLE IMSI_4 AS

SELECT MID,

SUM(MX_COA) AS COA_SUM

FROM IMSI

GROUP BY MID;

QUIT;

RUN;

PROC SQL;

CREATE TABLE IMSI_5 AS

SELECT MID,

SUM(MX_PLT) AS PLT_SUM

FROM IMSI

GROUP BY MID;

QUIT;

RUN;

DATA IMSI_SUM;

MERGE IMSI_1 IMSI_2 IMSI_3 IMSI_4 IMSI_5;

BY MID;

RUN;

DATA IMSI_T(KEEP=MID TX_CAN TX_CHOL TX_HTN TX_COA TX_PLT);

SET IMSI_SUM;

IF CAN_SUM>0 THEN TX_CAN=1; ELSE TX_CAN=0;

IF CHOL_SUM>0 THEN TX_CHOL=1; ELSE TX_CHOL=0;

IF HTN_SUM>0 THEN TX_HTN=1; ELSE TX_HTN=0;

IF COA_SUM>0 THEN TX_COA=1; ELSE TX_COA=0;

IF PLT_SUM>0 THEN TX_PLT=1; ELSE TX_PLT=0;

RUN;

*/

/*

DATA SHIM.TOTAL_2;

MERGE SHIM.TOTAL_1 SHIM.MID_SUM_1 SHIM.TYPE_S;

BY MID;

RUN;

PROC SORT DATA=SHIM.TOTAL_2 OUT=SHIM.TOTAL_S; BY JID RECU_FR_DD; RUN;

DATA SHIM.TOTAL_S;

SET SHIM.TOTAL_S;

IF MLIV=1 OR SLIV=1 THEN LIV=1; ELSE LIV=0;

RUN;

*/

/*

DATA SHIM.TOTAL_S_1;

SET SHIM.TOTAL_S;

YR=SUBSTR(RECU_FR_DD,1,4);

MO=SUBSTR(RECU_FR_DD,5,2);

DA=SUBSTR(RECU_FR_DD,7,2);

DXDATE=MDY(MO,DA,YR);

FORMAT DXDATE DATE9.;

FIDATE='31DEC2016'D;

FORMAT FIDATE DATE9.;

DIFFDA_1=DXDATE-'01JAN2013'D;

DIFFMO_1=DIFFDA_1/30;

DIFFMO_2=DXDATE-'30JUN2013'D;

IF '01JAN2012'D<=DXDATE<='31DEC2012'D THEN TAGE=PAT_AGE;

ELSE TAGE=.;

SAGE=0*(PAT_AGE<50)+1*(50<=PAT_AGE<60)+2*(60<=PAT_AGE<70)+3*(70<=PAT_AGE<80)+4*(80<=PAT_AGE);

NAGE=0*(PAT_AGE<55)+1*(55<=PAT_AGE<65)+2*(65<=PAT_AGE<75)+3*(75<=PAT_AGE);

BAGE=0*(PAT_AGE<65)+1*(65<=PAT_AGE);

SIDO=SUBSTR(RVD_PLC_CD,1,2);

IF SIDO='11' THEN KWON=1;

IF SIDO='22' OR SIDO='31' THEN KWON=2;

IF SIDO='25' OR SIDO='33' OR SIDO='34' OR SIDO='41' THEN KWON=3;

IF SIDO='32' THEN KWON=4;

IF SIDO='23' OR SIDO='37' THEN KWON=5;

IF SIDO='21' OR SIDO='26' OR SIDO='38' THEN KWON=6;

IF SIDO='24' OR SIDO='35' OR SIDO='36' OR SIDO='39' THEN KWON=7;

RUN;

DATA SHIM.TOTAL_S_2(KEEP=JID TX_CAN TX_CHOL TX_HTN TX_COA TX_PLT TX_ANT);

SET SHIM.TOTAL_S_1;

IF '01JAN2012'D<=DXDATE<='31DEC2012'D;

RUN;

PROC SQL;

CREATE TABLE SHIM.TOTAL_S_2_CAN AS

SELECT JID,

SUM(TX_CAN) AS CAN_SUM

FROM SHIM.TOTAL_S_2

GROUP BY JID;

QUIT;

RUN;

PROC SQL;

CREATE TABLE SHIM.TOTAL_S_2_CHOL AS

SELECT JID,

SUM(TX_CHOL) AS CHOL_SUM

FROM SHIM.TOTAL_S_2

GROUP BY JID;

QUIT;

RUN;

PROC SQL;

CREATE TABLE SHIM.TOTAL_S_2_HTN AS

SELECT JID,

SUM(TX_HTN) AS HTN_SUM

FROM SHIM.TOTAL_S_2

GROUP BY JID;

QUIT;

RUN;

PROC SQL;

CREATE TABLE SHIM.TOTAL_S_2_COA AS

SELECT JID,

SUM(TX_COA) AS COA_SUM

FROM SHIM.TOTAL_S_2

GROUP BY JID;

QUIT;

RUN;

PROC SQL;

CREATE TABLE SHIM.TOTAL_S_2_PLT AS

SELECT JID,

SUM(TX_PLT) AS PLT_SUM

FROM SHIM.TOTAL_S_2

GROUP BY JID;

QUIT;

PROC SQL;

CREATE TABLE SHIM.TOTAL_S_2_ANT AS

SELECT JID,

SUM(TX_ANT) AS ANT_SUM

FROM SHIM.TOTAL_S_2

GROUP BY JID;

QUIT;

RUN;

DATA SHIM.TOTAL_S_2_SUM;

MERGE SHIM.TOTAL_S_2_CAN SHIM.TOTAL_S_2_CHOL SHIM.TOTAL_S_2_HTN SHIM.TOTAL_S_2_COA

SHIM.TOTAL_S_2_PLT SHIM.TOTAL_S_2_ANT;

BY JID;

RUN;

DATA SHIM.TOTAL_S_2_SUM_1(KEEP=JID TTX_CAN TTX_CHOL TTX_HTN TTX_COA TTX_PLT TTX_ANT);

SET SHIM.TOTAL_S_2_SUM;

IF CAN_SUM>0 THEN TTX_CAN=1; ELSE TTX_CAN=0;

IF CHOL_SUM>0 THEN TTX_CHOL=1; ELSE TTX_CHOL=0;

IF HTN_SUM>0 THEN TTX_HTN=1; ELSE TTX_HTN=0;

IF COA_SUM>0 THEN TTX_COA=1; ELSE TTX_COA=0;

IF PLT_SUM>0 THEN TTX_PLT=1; ELSE TTX_PLT=0;

IF ANT_SUM>0 THEN TTX_ANT=1; ELSE TTX_ANT=0;

RUN;

DATA SHIM.TOTAL_S_3(KEEP=JID DM HTN IHD CVA ALZ MLIV SLIV LIV CAN AIDS CKD CHF MI COPD ASTH PVD HEMI CONN PUD);

SET SHIM.TOTAL_S_1;

IF '01JAN2012'D<=DXDATE<='31DEC2012'D;

RUN;

PROC SQL;

CREATE TABLE SHIM.TOTAL_S_3_DM AS

SELECT JID,

SUM(DM) AS DM_SUM

FROM SHIM.TOTAL_S_3

GROUP BY JID;

QUIT;

RUN;

PROC SQL;

CREATE TABLE SHIM.TOTAL_S_3_HTN AS

SELECT JID,

SUM(HTN) AS HTN_SUM

FROM SHIM.TOTAL_S_3

GROUP BY JID;

QUIT;

RUN;

PROC SQL;

CREATE TABLE SHIM.TOTAL_S_3_IHD AS

SELECT JID,

SUM(IHD) AS IHD_SUM

FROM SHIM.TOTAL_S_3

GROUP BY JID;

QUIT;

RUN;

PROC SQL;

CREATE TABLE SHIM.TOTAL_S_3_CVA AS

SELECT JID,

SUM(CVA) AS CVA_SUM

FROM SHIM.TOTAL_S_3

GROUP BY JID;

QUIT;

RUN;

PROC SQL;

CREATE TABLE SHIM.TOTAL_S_3_PKS AS

SELECT JID,

SUM(PKS) AS PKS_SUM

FROM SHIM.TOTAL_S_3

GROUP BY JID;

QUIT;

RUN;

PROC SQL;

CREATE TABLE SHIM.TOTAL_S_3_ALZ AS

SELECT JID,

SUM(ALZ) AS ALZ_SUM

FROM SHIM.TOTAL_S_3

GROUP BY JID;

QUIT;

RUN;

PROC SQL;

CREATE TABLE SHIM.TOTAL_S_3_MLIV AS

SELECT JID,

SUM(MLIV) AS MLIV_SUM

FROM SHIM.TOTAL_S_3

GROUP BY JID;

QUIT;

RUN;

PROC SQL;

CREATE TABLE SHIM.TOTAL_S_3_SLIV AS

SELECT JID,

SUM(SLIV) AS SLIV_SUM

FROM SHIM.TOTAL_S_3

GROUP BY JID;

QUIT;

RUN;

PROC SQL;

CREATE TABLE SHIM.TOTAL_S_3_LIV AS

SELECT JID,

SUM(LIV) AS LIV_SUM

FROM SHIM.TOTAL_S_3

GROUP BY JID;

QUIT;

RUN;

PROC SQL;

CREATE TABLE SHIM.TOTAL_S_3_CAN AS

SELECT JID,

SUM(CAN) AS CAN_SUM

FROM SHIM.TOTAL_S_3

GROUP BY JID;

QUIT;

RUN;

PROC SQL;

CREATE TABLE SHIM.TOTAL_S_3_AIDS AS

SELECT JID,

SUM(AIDS) AS AIDS_SUM

FROM SHIM.TOTAL_S_3

GROUP BY JID;

QUIT;

RUN;

PROC SQL;

CREATE TABLE SHIM.TOTAL_S_3_CKD AS

SELECT JID,

SUM(CKD) AS CKD_SUM

FROM SHIM.TOTAL_S_3

GROUP BY JID;

QUIT;

RUN;

PROC SQL;

CREATE TABLE SHIM.TOTAL_S_3_CHF AS

SELECT JID,

SUM(CHF) AS CHF_SUM

FROM SHIM.TOTAL_S_3

GROUP BY JID;

QUIT;

RUN;

PROC SQL;

CREATE TABLE SHIM.TOTAL_S_3_MI AS

SELECT JID,

SUM(MI) AS MI_SUM

FROM SHIM.TOTAL_S_3

GROUP BY JID;

QUIT;

RUN;

PROC SQL;

CREATE TABLE SHIM.TOTAL_S_3_COPD AS

SELECT JID,

SUM(COPD) AS COPD_SUM

FROM SHIM.TOTAL_S_3

GROUP BY JID;

QUIT;

RUN;

PROC SQL;

CREATE TABLE SHIM.TOTAL_S_3_ASTH AS

SELECT JID,

SUM(ASTH) AS ASTH_SUM

FROM SHIM.TOTAL_S_3

GROUP BY JID;

QUIT;

RUN;

PROC SQL;

CREATE TABLE SHIM.TOTAL_S_3_PVD AS

SELECT JID,

SUM(PVD) AS PVD_SUM

FROM SHIM.TOTAL_S_3

GROUP BY JID;

QUIT;

RUN;

PROC SQL;

CREATE TABLE SHIM.TOTAL_S_3_HEMI AS

SELECT JID,

SUM(HEMI) AS HEMI_SUM

FROM SHIM.TOTAL_S_3

GROUP BY JID;

QUIT;

RUN;

PROC SQL;

CREATE TABLE SHIM.TOTAL_S_3_CONN AS

SELECT JID,

SUM(CONN) AS CONN_SUM

FROM SHIM.TOTAL_S_3

GROUP BY JID;

QUIT;

RUN;

PROC SQL;

CREATE TABLE SHIM.TOTAL_S_3_PUD AS

SELECT JID,

SUM(PUD) AS PUD_SUM

FROM SHIM.TOTAL_S_3

GROUP BY JID;

QUIT;

RUN;

DATA SHIM.TOTAL_S_3_SUM;

MERGE SHIM.TOTAL_S_3_DM SHIM.TOTAL_S_3_HTN SHIM.TOTAL_S_3_IHD SHIM.TOTAL_S_3_CVA SHIM.TOTAL_S_3_PKS

SHIM.TOTAL_S_3_ALZ SHIM.TOTAL_S_3_MLIV SHIM.TOTAL_S_3_SLIV SHIM.TOTAL_S_3_LIV SHIM.TOTAL_S_3_CAN

SHIM.TOTAL_S_3_AIDS SHIM.TOTAL_S_3_CKD SHIM.TOTAL_S_3_CHF SHIM.TOTAL_S_3_MI SHIM.TOTAL_S_3_COPD

SHIM.TOTAL_S_3_ASTH SHIM.TOTAL_S_3_PVD SHIM.TOTAL_S_3_HEMI SHIM.TOTAL_S_3_CONN SHIM.TOTAL_S_3_PUD;

BY JID;

RUN;

DATA SHIM.TOTAL_S_3_SUM_1(KEEP=JID DDX_DM DDX_HTN DDX_IHD DDX_CVA DDX_PKS DDX_ALZ DDX_MLIV DDX_SLIV DDX_LIV

DDX_CAN DDX_AIDS DDX_CKD DDX_CHF DDX_MI DDX_COPD DDX_ASTH DDX_PVD DDX_HEMI DDX_CONN DDX_PUD);

SET SHIM.TOTAL_S_3_SUM;

IF DM_SUM>0 THEN DDX_DM=1; ELSE DDX_DM=0;

IF HTN_SUM>0 THEN DDX_HTN=1; ELSE DDX_HTN=0;

IF IHD_SUM>0 THEN DDX_IHD=1; ELSE DDX_IHD=0;

IF CVA_SUM>0 THEN DDX_CVA=1; ELSE DDX_CVA=0;

IF PKS_SUM>0 THEN DDX_PKS=1; ELSE DDX_PKS=0;

IF ALZ_SUM>0 THEN DDX_ALZ=1; ELSE DDX_ALZ=0;

IF MLIV_SUM>0 THEN DDX_MLIV=1; ELSE DDX_MLIV=0;

IF SLIV_SUM>0 THEN DDX_SLIV=1; ELSE DDX_SLIV=0;

IF LIV_SUM>0 THEN DDX_LIV=1; ELSE DDX_LIV=0;

IF CAN_SUM>0 THEN DDX_CAN=1; ELSE DDX_CAN=0;

IF AIDS_SUM>0 THEN DDX_AIDS=1; ELSE DDX_AIDS=0;

IF CKD_SUM>0 THEN DDX_CKD=1; ELSE DDX_CKD=0;

IF CHF_SUM>0 THEN DDX_CHF=1; ELSE DDX_CHF=0;

IF MI_SUM>0 THEN DDX_MI=1; ELSE DDX_MI=0;

IF COPD_SUM>0 THEN DDX_COPD=1; ELSE DDX_COPD=0;

IF ASTH_SUM>0 THEN DDX_ASTH=1; ELSE DDX_ASTH=0;

IF PVD_SUM>0 THEN DDX_PVD=1; ELSE DDX_PVD=0;

IF HEMI_SUM>0 THEN DDX_HEMI=1; ELSE DDX_HEMI=0;

IF CONN_SUM>0 THEN DDX_CONN=1; ELSE DDX_CONN=0;

IF PUD_SUM>0 THEN DDX_PUD=1; ELSE DDX_PUD=0;

RUN;

DATA SHIM.TOTAL_S_4;

MERGE SHIM.TOTAL_S_1 SHIM.TOTAL_S_2_SUM_1 SHIM.TOTAL_S_3_SUM_1;

BY JID;

RUN;

DATA SHIM.TOTAL_S_NEW;

SET SHIM.TOTAL_S_4;

IF TX_CAN=. THEN TX_CAN=0;

IF TX_CHOL=. THEN TX_CHOL=0;

IF TX_HTN=. THEN TX_HTN=0;

IF TX_COA=. THEN TX_COA=0;

IF TX_PLT=. THEN TX_PLT=0;

IF TX_ANT=. THEN TX_ANT=0;

IF TTX_CAN=. THEN TTX_CAN=0;

IF TTX_CHOL=. THEN TTX_CHOL=0;

IF TTX_HTN=. THEN TTX_HTN=0;

IF TTX_COA=. THEN TTX_COA=0;

IF TTX_PLT=. THEN TTX_PLT=0;

IF TTX_ANT=. THEN TTX_ANT=0;

RUN;

DATA SHIM.TOTAL_S_NEW_1;

SET SHIM.TOTAL_S_NEW;

IF '110001'<=RVD_PLC_CD<='210015' THEN CITY=1;

ELSE IF '220001'<=RVD_PLC_CD<='220008' THEN CITY=1;

ELSE IF '230001'<=RVD_PLC_CD<='230007' THEN CITY=1;

ELSE IF '240001'<=RVD_PLC_CD<='260004' THEN CITY=1;

ELSE IF '310301'<=RVD_PLC_CD<='312003' THEN CITY=1;

ELSE IF '320400'<=RVD_PLC_CD<='320500' THEN CITY=1;

ELSE IF '330100'<=RVD_PLC_CD<='330200' THEN CITY=1;

ELSE IF '340200'<=RVD_PLC_CD<='340202' THEN CITY=1;

ELSE IF '350401'<=RVD_PLC_CD<='350402' THEN CITY=1;

ELSE IF '360200'<=RVD_PLC_CD<='360700' THEN CITY=1;

ELSE IF '370100'<=RVD_PLC_CD<='370300' THEN CITY=1;

ELSE IF '370700'<=RVD_PLC_CD<='370702' THEN CITY=1;

ELSE IF '380100'<=RVD_PLC_CD<='380800' THEN CITY=1;

ELSE IF '390200'<=RVD_PLC_CD<='410000' THEN CITY=1;

ELSE CITY=0;

IF RVD_PLC_CD='310006' THEN CITY=1;

IF RVD_PLC_CD='310016' THEN CITY=1;

IF RVD_PLC_CD='310100' THEN CITY=1;

IF RVD_PLC_CD='312300' THEN CITY=1;

IF RVD_PLC_CD='381100' THEN CITY=1;

RUN;

DATA SHIM.TOTAL_S_NEW_3;

SET SHIM.TOTAL_S_NEW_1;

IF DDX_DM=. THEN DDX_DM=0;

IF DDX_HTN=. THEN DDX_HTN=0;

IF DDX_IHD=. THEN DDX_IHD=0;

IF DDX_CVA=. THEN DDX_CVA=0;

IF DDX_PKS=. THEN DDX_PKS=0;

IF DDX_ALZ=. THEN DDX_ALZ=0;

IF DDX_MLIV=. THEN DDX_MLIV=0;

IF DDX_SLIV=. THEN DDX_SLIV=0;

IF DDX_LIV=. THEN DDX_LIV=0;

IF DDX_CAN=. THEN DDX_CAN=0;

IF DDX_AIDS=. THEN DDX_AIDS=0;

IF DDX_CKD=. THEN DDX_CKD=0;

IF DDX_CHF=. THEN DDX_CHF=0;

IF DDX_MI=. THEN DDX_MI=0;

IF DDX_COPD=. THEN DDX_COPD=0;

IF DDX_ASTH=. THEN DDX_ASTH=0;

IF DDX_PVD=. THEN DDX_PVD=0;

IF DDX_HEMI=. THEN DDX_HEMI=0;

IF DDX_CONN=. THEN DDX_CONN=0;

IF DDX_PUD=. THEN DDX_PUD=0;

RUN;

*=============================================================================;

*=============================================================================;

DATA SHIM.TIME_1(KEEP=JID ADT_1 ADT_3 ADT_4 ADT_6);

SET SHIM.TOTAL_S_NEW_3;

RUN;

PROC SQL;

CREATE TABLE SHIM.TIME_1_ADT_1 AS

SELECT JID,

SUM(ADT_1) AS ADT_1_SUM

FROM SHIM.TIME_1

GROUP BY JID;

QUIT;

RUN;

PROC SQL;

CREATE TABLE SHIM.TIME_1_ADT_3 AS

SELECT JID,

SUM(ADT_3) AS ADT_3_SUM

FROM SHIM.TIME_1

GROUP BY JID;

QUIT;

RUN;

PROC SQL;

CREATE TABLE SHIM.TIME_1_ADT_4 AS

SELECT JID,

SUM(ADT_4) AS ADT_4_SUM

FROM SHIM.TIME_1

GROUP BY JID;

QUIT;

RUN;

PROC SQL;

CREATE TABLE SHIM.TIME_1_ADT_6 AS

SELECT JID,

SUM(ADT_6) AS ADT_6_SUM

FROM SHIM.TIME_1

GROUP BY JID;

QUIT;

RUN;

DATA SHIM.TIME_IM;

MERGE SHIM.TIME_1_ADT_1 SHIM.TIME_1_ADT_3 SHIM.TIME_1_ADT_4 SHIM.TIME_1_ADT_6;

BY JID;

RUN;

DATA SHIM.TIME_TOT;

SET SHIM.TIME_IM;

ADT_MO=ADT_1_SUM*1+ADT_3_SUM*3+ADT_4_SUM*4+ADT_6_SUM*6;

ADT_CAT=0*(ADT_MO=0)+1*(0<ADT_MO<=3)+2*(3<ADT_MO<=6)+3*(6<ADT_MO<=24)+4*(24<ADT_MO);

RUN;

DATA SHIM.TOTAL_S_NEW_6;

MERGE SHIM.TOTAL_S_NEW_3 SHIM.TIME_TOT;

BY JID;

RUN;

*/

*================================;

*================================;

/*

DATA SHIM.JID_2012 (KEEP=JID STAND);

SET SHIM.TOTAL_S_NEW_6;

IF '01JAN2012'D<=DXDATE<='31DEC2012'D;

STAND=1;

RUN;

PROC SORT DATA=SHIM.JID_2012 OUT=SHIM.JID_2012_1 NODUPKEY; BY JID; RUN;

DATA SHIM.TOTAL_S_NEW_7;

MERGE SHIM.TOTAL_S_NEW_6 SHIM.JID_2012_1;

BY JID;

RUN;

DATA SHIM.TOTAL_S_NEW_8 (DROP=STAND);

SET SHIM.TOTAL_S_NEW_7;

IF STAND=. THEN DELETE;

RUN;

*/

*==================================================;

*==================================================;

/*

DATA SHIM.CONTROL;

SET SHIM.TOTAL_S_NEW_8;

KEEP MID JID DXDATE ADT_1 ADT_3 ADT_4 ADT_6;

RUN;

DATA SHIM.CONTROL_1(KEEP=MID JID DXDATE ADT);

SET SHIM.CONTROL;

IF ADT_1=1 OR ADT_3=1 OR ADT_4=1 OR ADT_6=1 THEN ADT=1; ELSE ADT=2;

RUN;

DATA SHIM.CONTROL_2_1;

SET SHIM.CONTROL_1;

IF ADT=1 AND '01JAN2012'D<=DXDATE<='30JUN2012'D THEN CAT_1=1; ELSE CAT_1=2;

RUN;

DATA SHIM.CONTROL_2_2;

SET SHIM.CONTROL_1;

IF ADT=1 AND '01JUL2012'D<=DXDATE<='31DEC2012'D THEN CAT_2=1; ELSE CAT_2=2;

RUN;

DATA SHIM.CONTROL_2_3;

SET SHIM.CONTROL_1;

IF ADT=1 AND '01JAN2013'D<=DXDATE<='31DEC2016'D THEN CAT_3=1; ELSE CAT_3=2;

RUN;

PROC SORT DATA=SHIM.CONTROL_2_1 OUT=SHIM.CONTROL_2_1_S; BY JID CAT_1 DXDATE; RUN;

PROC SORT DATA=SHIM.CONTROL_2_1_S OUT=SHIM.CONTROL_2_1_SS NODUPKEY; BY JID; RUN;

PROC SORT DATA=SHIM.CONTROL_2_2 OUT=SHIM.CONTROL_2_2_S; BY JID CAT_2 DXDATE; RUN;

PROC SORT DATA=SHIM.CONTROL_2_2_S OUT=SHIM.CONTROL_2_2_SS NODUPKEY; BY JID; RUN;

PROC SORT DATA=SHIM.CONTROL_2_3 OUT=SHIM.CONTROL_2_3_S; BY JID CAT_3 DXDATE; RUN;

PROC SORT DATA=SHIM.CONTROL_2_3_S OUT=SHIM.CONTROL_2_3_SS NODUPKEY; BY JID; RUN;

DATA SHIM.CONTROL_3;

MERGE SHIM.CONTROL_2_1_SS SHIM.CONTROL_2_2_SS SHIM.CONTROL_2_3_SS;

BY JID;

RUN;

DATA SHIM.CONTROL_4;

SET SHIM.CONTROL_3;

IF CAT_1=1 AND CAT_2=1 AND CAT_3=1 THEN ADT_GR=1;

IF CAT_1=1 AND CAT_2=1 AND CAT_3=2 THEN ADT_GR=2;

IF CAT_1=1 AND CAT_2=2 AND CAT_3=1 THEN ADT_GR=3;

IF CAT_1=1 AND CAT_2=2 AND CAT_3=2 THEN ADT_GR=4;

IF CAT_1=2 AND CAT_2=1 AND CAT_3=1 THEN ADT_GR=5;

IF CAT_1=2 AND CAT_2=1 AND CAT_3=2 THEN ADT_GR=6;

IF CAT_1=2 AND CAT_2=2 AND CAT_3=1 THEN ADT_GR=7;

IF CAT_1=2 AND CAT_2=2 AND CAT_3=2 THEN ADT_GR=8;

RUN;

*/

/*

PROC FREQ DATA=SHIM.CONTROL_4;

TABLES ADT_GR;

RUN;

*/

/*

DATA SHIM.CONTROL_5;

SET SHIM.CONTROL_4;

IF ADT_GR=5 OR ADT_GR=6 THEN CASE=1;

ELSE IF ADT_GR=8 THEN CASE=2;

ELSE CASE=.;

RUN;

DATA SHIM.CONTROL_6(KEEP=JID CASE);

SET SHIM.CONTROL_5;

IF CASE=1 OR CASE=2;

RUN;

DATA SHIM.TOTAL_S_NEW_8_1;

MERGE SHIM.TOTAL_S_NEW_8 SHIM.CONTROL_6;

BY JID;

RUN;

DATA SHIM.TOTAL_S_NEW_8_2;

SET SHIM.TOTAL_S_NEW_8_1;

IF CASE=. THEN DELETE;

RUN;

DATA SHIM.TOTAL_S_NEW_8_3;

SET SHIM.TOTAL_S_NEW_8_2;

IF DDX_CVA=1 THEN DELETE;

IF DDX_MI=1 THEN DELETE;

IF DDX_IHD=1 THEN DELETE;

IF DDX_DM=1 THEN DELETE;

IF TTX_CAN=1 THEN DELETE;

RUN;

DATA SHIM.TOTAL_S_NEW_8_4;

SET SHIM.TOTAL_S_NEW_8_2;

IF DDX_ALZ=1 THEN DELETE;

IF DDX_PKS=1 THEN DELETE;

IF TTX_CAN=1 THEN DELETE;

RUN;

DATA SHIM.DIS_1;

SET SHIM.TOTAL_S_NEW_8_3;

IF DM=0 THEN Y_DM=2; ELSE IF DM=1 THEN Y_DM=1; ELSE Y_DM=3;

IF IHD=0 THEN Y_IHD=2; ELSE IF IHD=1 THEN Y_IHD=1; ELSE Y_IHD=3;

IF CVA=0 THEN Y_CVA=2; ELSE IF CVA=1 THEN Y_CVA=1; ELSE Y_CVA=3;

IF MI=0 THEN Y_MI=2; ELSE IF MI=1 THEN Y_MI=1; ELSE Y_MI=3;

IF '01JAN2012'D<=DXDATE<='31DEC2012'D AND (CVA=1 OR DM=1 OR IHD=1 OR MI=1) THEN DELETE;

DIFFDA_2=DXDATE-'31DEC2012'D;

RUN;

PROC SORT DATA=SHIM.DIS_1 OUT=SHIM.DIS_1_DM; BY JID Y_DM DXDATE; RUN;

PROC SORT DATA=SHIM.DIS_1_DM OUT=SHIM.DIS_1_DM_S NODUPKEY; BY JID; RUN;

PROC SORT DATA=SHIM.DIS_1 OUT=SHIM.DIS_1_IHD; BY JID Y_IHD DXDATE; RUN;

PROC SORT DATA=SHIM.DIS_1_IHD OUT=SHIM.DIS_1_IHD_S NODUPKEY; BY JID; RUN;

PROC SORT DATA=SHIM.DIS_1 OUT=SHIM.DIS_1_CVA; BY JID Y_CVA DXDATE; RUN;

PROC SORT DATA=SHIM.DIS_1_CVA OUT=SHIM.DIS_1_CVA_S NODUPKEY; BY JID; RUN;

PROC SORT DATA=SHIM.DIS_1 OUT=SHIM.DIS_1_MI; BY JID Y_MI DXDATE; RUN;

PROC SORT DATA=SHIM.DIS_1_MI OUT=SHIM.DIS_1_MI_S NODUPKEY; BY JID; RUN;

DATA SHIM.DIS_2;

SET SHIM.TOTAL_S_NEW_8_4;

IF PKS=0 THEN Y_PKS=2; ELSE IF PKS=1 THEN Y_PKS=1; ELSE Y_PKS=3;

IF ALZ=0 THEN Y_ALZ=2; ELSE IF ALZ=1 THEN Y_ALZ=1; ELSE Y_ALZ=3;

IF '01JAN2012'D<=DXDATE<='31DEC2012'D AND (ALZ=1 OR PKS=1) THEN DELETE;

DIFFDA_2=DXDATE-'31DEC2012'D;

RUN;

PROC SORT DATA=SHIM.DIS_2 OUT=SHIM.DIS_2_PKS; BY JID Y_PKS DXDATE; RUN;

PROC SORT DATA=SHIM.DIS_2_PKS OUT=SHIM.DIS_2_PKS_S NODUPKEY; BY JID; RUN;

PROC SORT DATA=SHIM.DIS_2 OUT=SHIM.DIS_2_ALZ; BY JID Y_ALZ DXDATE; RUN;

PROC SORT DATA=SHIM.DIS_2_ALZ OUT=SHIM.DIS_2_ALZ_S NODUPKEY; BY JID; RUN;

*===================================================;

*=================== 2018.03.29.======================;

*===================================================;

DATA SHIM.DIS_1_CVA_S_1(DROP=DM IHD MI ALZ PKS);

SET SHIM.DIS_1_CVA_S;

CHARLSON_1=SUM(OF SAGE,DDX_DM,DDX_MLIV,3*DDX_SLIV,2*DDX_CAN,6*DDX_AIDS,2*DDX_CKD,

DDX_CHF,DDX_MI,DDX_COPD,DDX_PVD,DDX_CVA,DDX_ALZ,2*DDX_HEMI,DDX_CONN,DDX_PUD);

IF Y_CVA=3 THEN CVA=0;

IF CVA=0 AND DIFFDA_1<0 THEN DIFFDA_2='31DEC2016'D-'31DEC2012'D;

DIFFMO_2=DIFFDA_2*12/365.25;

RUN;

DATA SHIM.DIS_1_DM_S_1(DROP=CVA IHD MI ALZ PKS);

SET SHIM.DIS_1_DM_S;

CHARLSON_1=SUM(OF SAGE,DDX_DM,DDX_MLIV,3*DDX_SLIV,2*DDX_CAN,6*DDX_AIDS,2*DDX_CKD,

DDX_CHF,DDX_MI,DDX_COPD,DDX_PVD,DDX_CVA,DDX_ALZ,2*DDX_HEMI,DDX_CONN,DDX_PUD);

IF Y_DM=3 THEN DM=0;

IF DM=0 AND DIFFDA_1<0 THEN DIFFDA_2='31DEC2016'D-'31DEC2012'D;

DIFFMO_2=DIFFDA_2*12/365.25;

RUN;

DATA SHIM.DIS_1_IHD_S_1(DROP=CVA DM MI ALZ PKS);

SET SHIM.DIS_1_IHD_S;

CHARLSON_1=SUM(OF SAGE,DDX_DM,DDX_MLIV,3*DDX_SLIV,2*DDX_CAN,6*DDX_AIDS,2*DDX_CKD,

DDX_CHF,DDX_MI,DDX_COPD,DDX_PVD,DDX_CVA,DDX_ALZ,2*DDX_HEMI,DDX_CONN,DDX_PUD);

IF Y_IHD=3 THEN IHD=0;

IF IHD=0 AND DIFFDA_1<0 THEN DIFFDA_2='31DEC2016'D-'31DEC2012'D;

DIFFMO_2=DIFFDA_2*12/365.25;

RUN;

DATA SHIM.DIS_1_MI_S_1(DROP=CVA DM IHD ALZ PKS);

SET SHIM.DIS_1_MI_S;

CHARLSON_1=SUM(OF SAGE,DDX_DM,DDX_MLIV,3*DDX_SLIV,2*DDX_CAN,6*DDX_AIDS,2*DDX_CKD,

DDX_CHF,DDX_MI,DDX_COPD,DDX_PVD,DDX_CVA,DDX_ALZ,2*DDX_HEMI,DDX_CONN,DDX_PUD);

IF Y_MI=3 THEN MI=0;

IF MI=0 AND DIFFDA_1<0 THEN DIFFDA_2='31DEC2016'D-'31DEC2012'D;

DIFFMO_2=DIFFDA_2*12/365.25;

RUN;

DATA SHIM.DIS_2_ALZ_S_1(DROP=CVA DM IHD MI PKS);

SET SHIM.DIS_2_ALZ_S;

CHARLSON_1=SUM(OF SAGE,DDX_DM,DDX_MLIV,3*DDX_SLIV,2*DDX_CAN,6*DDX_AIDS,2*DDX_CKD,

DDX_CHF,DDX_MI,DDX_COPD,DDX_PVD,DDX_CVA,DDX_ALZ,2*DDX_HEMI,DDX_CONN,DDX_PUD);

IF Y_ALZ=3 THEN ALZ=0;

IF ALZ=0 AND DIFFDA_1<0 THEN DIFFDA_2='31DEC2016'D-'31DEC2012'D;

DIFFMO_2=DIFFDA_2*12/365.25;

RUN;

DATA SHIM.DIS_2_PKS_S_1(DROP=CVA DM IHD MI ALZ);

SET SHIM.DIS_2_PKS_S;

CHARLSON_1=SUM(OF SAGE,DDX_DM,DDX_MLIV,3*DDX_SLIV,2*DDX_CAN,6*DDX_AIDS,2*DDX_CKD,

DDX_CHF,DDX_MI,DDX_COPD,DDX_PVD,DDX_CVA,DDX_ALZ,2*DDX_HEMI,DDX_CONN,DDX_PUD);

IF Y_PKS=3 THEN PKS=0;

IF PKS=0 AND DIFFDA_1<0 THEN DIFFDA_2='31DEC2016'D-'31DEC2012'D;

DIFFMO_2=DIFFDA_2*12/365.25;

RUN;

*/

/*

DATA SHIM.DXDX_T;

SET SHIM.T200_2018Q1_09;

PCA_1=SUBSTR(MAIN_SICK,1,3);

PCA_2=SUBSTR(SUB_SICK,1,3);

YR=SUBSTR(RECU_FR_DD,1,4);

MO=SUBSTR(RECU_FR_DD,5,2);

DA=SUBSTR(RECU_FR_DD,7,2);

DXDATE=MDY(MO,DA,YR);

FORMAT DXDATE DATE9.;

IF '01JAN2012'D<=DXDATE<='31DEC2012'D AND (PCA_1='C61' OR PCA_2='C61');

PCA_T=1;

RUN;

PROC SQL;

CREATE TABLE SHIM.DXDX_TT AS

SELECT JID,

SUM(PCA_T) AS PCA_T_SUM

FROM SHIM.DXDX_T

GROUP BY JID;

QUIT;

RUN;

*/

/*

DATA SHIM.DXDX_T_1;

SET SHIM.T200_2018Q1_09;

PCA_1=SUBSTR(MAIN_SICK,1,3);

PCA_2=SUBSTR(SUB_SICK,1,3);

YR=SUBSTR(RECU_FR_DD,1,4);

MO=SUBSTR(RECU_FR_DD,5,2);

DA=SUBSTR(RECU_FR_DD,7,2);

DXDATE=MDY(MO,DA,YR);

FORMAT DXDATE DATE9.;

IF '01JAN2012'D<=DXDATE<='31DEC2016'D AND (PCA_1='C61' OR PCA_2='C61');

PCA_T=1;

RUN;

PROC SQL;

CREATE TABLE SHIM.DXDX_TT_1 AS

SELECT JID,

SUM(PCA_T) AS PCA_T_SUM

FROM SHIM.DXDX_T_1

GROUP BY JID;

QUIT;

RUN;

DATA SHIM.DXDX_TT_2;

SET SHIM.DXDX_TT_1;

IF PCA_T_SUM>=2;

RUN;

*/

/*

DATA SHIM.DXDX_AF;

SET SHIM.T200_2018Q1_09;

PCA_1=SUBSTR(MAIN_SICK,1,3);

PCA_2=SUBSTR(SUB_SICK,1,3);

YR=SUBSTR(RECU_FR_DD,1,4);

MO=SUBSTR(RECU_FR_DD,5,2);

DA=SUBSTR(RECU_FR_DD,7,2);

DXDATE=MDY(MO,DA,YR);

FORMAT DXDATE DATE9.;

IF '01JUL2012'D<=DXDATE<='31DEC2012'D AND (PCA_1='C61' OR PCA_2='C61');

PCA_AF=1;

RUN;

PROC SQL;

CREATE TABLE SHIM.DXDX_AFAF AS

SELECT JID,

SUM(PCA_AF) AS PCA_AF_SUM

FROM SHIM.DXDX_AF

GROUP BY JID;

QUIT;

RUN;

DATA SHIM.DXDX_NEW;

MERGE SHIM.DXDX_TT SHIM.DXDX_AFAF;

BY JID;

RUN;

DATA SHIM.DXDX_NEW_1;

SET SHIM.DXDX_NEW;

IF PCA_T_SUM=PCA_AF_SUM THEN DO;

ICDCASE=1;

END;

RUN;

DATA SHIM.DXDX_NEW_2;

SET SHIM.DXDX_NEW_1;

IF ICDCASE=1;

RUN;

DATA SHIM.DXDX_NEW_3(KEEP=JID PCA_T_SUM OPT_CASE);

SET SHIM.DXDX_NEW_2;

OPT_CASE=1;

RUN;

DATA SHIM.DXDX_NEW_4;

SET SHIM.DXDX_NEW_3;

IF PCA_T_SUM>=2;

RUN;

*/

/*

DATA SHIM.TEMP_1 (KEEP=JID TEMP);

SET SHIM.CONTROL_4;

IF ADT_GR=8;

TEMP=1;

RUN;

*/

/*

DATA SHIM.DXDX_NEW_TEMP;

MERGE SHIM.DXDX_NEW_4 SHIM.TEMP_1;

BY JID;

RUN;

DATA SHIM.DXDX_NEW_TEMP_1;

SET SHIM.DXDX_NEW_TEMP;

IF PCA_T_SUM=. THEN DELETE;

RUN;

*/

/*

PROC FREQ DATA=SHIM.DXDX_NEW_TEMP_1;

TABLES TEMP;

RUN;

*/

*===========================================;

*===========================================;

*============= 2018.03.29====================;

*===========================================;

/*

DATA SHIM.DIS_1_CVA_S_2;

MERGE SHIM.DIS_1_CVA_S_1 SHIM.DXDX_NEW_4;

BY JID;

RUN;

DATA SHIM.DIS_1_CVA_S_3;

SET SHIM.DIS_1_CVA_S_2;

IF CASE=1 OR (CASE=2 AND OPT_CASE=1);

RUN;

DATA SHIM.DIS_1_DM_S_2;

MERGE SHIM.DIS_1_DM_S_1 SHIM.DXDX_NEW_4;

BY JID;

RUN;

DATA SHIM.DIS_1_DM_S_3;

SET SHIM.DIS_1_DM_S_2;

IF CASE=1 OR (CASE=2 AND OPT_CASE=1);

RUN;

DATA SHIM.DIS_1_IHD_S_2;

MERGE SHIM.DIS_1_IHD_S_1 SHIM.DXDX_NEW_4;

BY JID;

RUN;

DATA SHIM.DIS_1_IHD_S_3;

SET SHIM.DIS_1_IHD_S_2;

IF CASE=1 OR (CASE=2 AND OPT_CASE=1);

RUN;

DATA SHIM.DIS_1_MI_S_2;

MERGE SHIM.DIS_1_MI_S_1 SHIM.DXDX_NEW_4;

BY JID;

RUN;

DATA SHIM.DIS_1_MI_S_3;

SET SHIM.DIS_1_MI_S_2;

IF CASE=1 OR (CASE=2 AND OPT_CASE=1);

RUN;

DATA SHIM.DIS_2_ALZ_S_2;

MERGE SHIM.DIS_2_ALZ_S_1 SHIM.DXDX_NEW_4;

BY JID;

RUN;

DATA SHIM.DIS_2_ALZ_S_3;

SET SHIM.DIS_2_ALZ_S_2;

IF CASE=1 OR (CASE=2 AND OPT_CASE=1);

RUN;

DATA SHIM.DIS_2_PKS_S_2;

MERGE SHIM.DIS_2_PKS_S_1 SHIM.DXDX_NEW_4;

BY JID;

RUN;

DATA SHIM.DIS_2_PKS_S_3;

SET SHIM.DIS_2_PKS_S_2;

IF CASE=1 OR (CASE=2 AND OPT_CASE=1);

RUN;

*/

/*

DATA SHIM.DIS_1_CVA_S_4;

SET SHIM.DIS_1_CVA_S_3;

KEEP

JID PAT_AGE INSUP_TP_CD KWON

TTX_CAN TTX_CHOL TTX_HTN TTX_COA TTX_PLT TTX_ANT

DDX_DM DDX_HTN DDX_IHD DDX_CVA DDX_PKS DDX_ALZ DDX_LIV DDX_CAN DDX_CKD DDX_CHF DDX_MI DDX_COPD

DDX_ASTH DDX_PVD

CITY ADT_MO ADT_CAT

CASE

CHARLSON_1

DIFFMO_2

CVA;

RUN;

DATA SHIM.DIS_1_IHD_S_4;

SET SHIM.DIS_1_IHD_S_3;

KEEP

JID PAT_AGE INSUP_TP_CD KWON

TTX_CAN TTX_CHOL TTX_HTN TTX_COA TTX_PLT TTX_ANT

DDX_DM DDX_HTN DDX_IHD DDX_CVA DDX_PKS DDX_ALZ DDX_LIV DDX_CAN DDX_CKD DDX_CHF DDX_MI DDX_COPD

DDX_ASTH DDX_PVD

CITY ADT_MO ADT_CAT

CASE

CHARLSON_1

DIFFMO_2

IHD;

RUN;

DATA SHIM.DIS_1_DM_S_4;

SET SHIM.DIS_1_DM_S_3;

KEEP

JID PAT_AGE INSUP_TP_CD KWON

TTX_CAN TTX_CHOL TTX_HTN TTX_COA TTX_PLT TTX_ANT

DDX_DM DDX_HTN DDX_IHD DDX_CVA DDX_PKS DDX_ALZ DDX_LIV DDX_CAN DDX_CKD DDX_CHF DDX_MI DDX_COPD

DDX_ASTH DDX_PVD

CITY ADT_MO ADT_CAT

CASE

CHARLSON_1

DIFFMO_2

DM;

RUN;

DATA SHIM.DIS_1_MI_S_4;

SET SHIM.DIS_1_MI_S_3;

KEEP

JID PAT_AGE INSUP_TP_CD KWON

TTX_CAN TTX_CHOL TTX_HTN TTX_COA TTX_PLT TTX_ANT

DDX_DM DDX_HTN DDX_IHD DDX_CVA DDX_PKS DDX_ALZ DDX_LIV DDX_CAN DDX_CKD DDX_CHF DDX_MI DDX_COPD

DDX_ASTH DDX_PVD

CITY ADT_MO ADT_CAT

CASE

CHARLSON_1

DIFFMO_2

MI;

RUN;

DATA SHIM.DIS_2_ALZ_S_4;

SET SHIM.DIS_2_ALZ_S_3;

KEEP

JID PAT_AGE INSUP_TP_CD KWON

TTX_CAN TTX_CHOL TTX_HTN TTX_COA TTX_PLT TTX_ANT

DDX_DM DDX_HTN DDX_IHD DDX_CVA DDX_PKS DDX_ALZ DDX_LIV DDX_CAN DDX_CKD DDX_CHF DDX_MI DDX_COPD

DDX_ASTH DDX_PVD

CITY ADT_MO ADT_CAT

CASE

CHARLSON_1

DIFFMO_2

ALZ;

RUN;

DATA SHIM.DIS_2_PKS_S_4;

SET SHIM.DIS_2_PKS_S_3;

KEEP

JID PAT_AGE INSUP_TP_CD KWON

TTX_CAN TTX_CHOL TTX_HTN TTX_COA TTX_PLT TTX_ANT

DDX_DM DDX_HTN DDX_IHD DDX_CVA DDX_PKS DDX_ALZ DDX_LIV DDX_CAN DDX_CKD DDX_CHF DDX_MI DDX_COPD

DDX_ASTH DDX_PVD

CITY ADT_MO ADT_CAT

CASE

CHARLSON_1

DIFFMO_2

PKS;

RUN;

*/

/*===============ADT==================

PROC SQL;

CREATE TABLE SHIM.TOTAL_S_NEW_8_ADT_1 AS

SELECT JID,

SUM(ADT_1) AS ADT_1_SUM

FROM SHIM.TOTAL_S_NEW_8

GROUP BY JID;

QUIT;

RUN;

PROC SQL;

CREATE TABLE SHIM.TOTAL_S_NEW_8_ADT_3 AS

SELECT JID,

SUM(ADT_3) AS ADT_3_SUM

FROM SHIM.TOTAL_S_NEW_8

GROUP BY JID;

QUIT;

RUN;

PROC SQL;

CREATE TABLE SHIM.TOTAL_S_NEW_8_ADT_4 AS

SELECT JID,

SUM(ADT_4) AS ADT_4_SUM

FROM SHIM.TOTAL_S_NEW_8

GROUP BY JID;

QUIT;

RUN;

PROC SQL;

CREATE TABLE SHIM.TOTAL_S_NEW_8_ADT_6 AS

SELECT JID,

SUM(ADT_6) AS ADT_6_SUM

FROM SHIM.TOTAL_S_NEW_8

GROUP BY JID;

QUIT;

RUN;

DATA SHIM.TOTAL_S_NEW_8_ADT_TOT;

MERGE SHIM.TOTAL_S_NEW_8_ADT_1 SHIM.TOTAL_S_NEW_8_ADT_3 SHIM.TOTAL_S_NEW_8_ADT_4

SHIM.TOTAL_S_NEW_8_ADT_6;

BY JID;

RUN;

DATA SHIM.TOTAL_S_NEW_8_ADT_TOT_1;

SET SHIM.TOTAL_S_NEW_8_ADT_TOT;

ADT_1346=ADT_1_SUM+3*ADT_3_SUM+4*ADT_4_SUM+6*ADT_6_SUM;

RUN;

PROC FREQ DATA=SHIM.TOTAL_S_NEW_8_ADT_TOT_1;

TABLES ADT_1346;

RUN;

=============================================*/

/*

DATA SHIM.DIS_1_CVA_S_5;

SET SHIM.DIS_1_CVA_S_4;

IF PAT_AGE<40 THEN DELETE;

RUN;

DATA SHIM.DIS_1_IHD_S_5;

SET SHIM.DIS_1_IHD_S_4;

IF PAT_AGE<40 THEN DELETE;

RUN;

DATA SHIM.DIS_1_MI_S_5;

SET SHIM.DIS_1_MI_S_4;

IF PAT_AGE<40 THEN DELETE;

RUN;

DATA SHIM.DIS_1_DM_S_5;

SET SHIM.DIS_1_DM_S_4;

IF PAT_AGE<40 THEN DELETE;

RUN;

DATA SHIM.DIS_2_ALZ_S_5;

SET SHIM.DIS_2_ALZ_S_4;

IF PAT_AGE<40 THEN DELETE;

RUN;

DATA SHIM.DIS_2_PKS_S_5;

SET SHIM.DIS_2_PKS_S_4;

IF PAT_AGE<40 THEN DELETE;

RUN;

*/

/*

*/

/*

DATA SHIM.DIS_1_CVA_S_6;

SET SHIM.DIS_1_CVA_S_5;

SAGE=0*(PAT_AGE<50)+1*(50<=PAT_AGE<60)+2*(60<=PAT_AGE<70)+3*(70<=PAT_AGE<80)+4*(80<=PAT_AGE);

NAGE=0*(PAT_AGE<55)+1*(55<=PAT_AGE<65)+2*(65<=PAT_AGE<75)+3*(75<=PAT_AGE);

BAGE=0*(PAT_AGE<65)+1*(65<=PAT_AGE);

ADT_NEW_CAT=1*(ADT_MO<6)+2*(6<=ADT_MO<24)+3*(24<=ADT_MO);

CHA_NEW=1*(CHARLSON_1<=1)+2*(CHARLSON_1=2)+3*(CHARLSON_1=3)+4*(CHARLSON_1=4)+5*(5<=CHARLSON_1);

INSUP=1*(INSUP_TP_CD='4')+2*(INSUP_TP_CD='5' OR INSUP_TP_CD='7');

RUN;

DATA SHIM.DIS_1_IHD_S_6;

SET SHIM.DIS_1_IHD_S_5;

SAGE=0*(PAT_AGE<50)+1*(50<=PAT_AGE<60)+2*(60<=PAT_AGE<70)+3*(70<=PAT_AGE<80)+4*(80<=PAT_AGE);

NAGE=0*(PAT_AGE<55)+1*(55<=PAT_AGE<65)+2*(65<=PAT_AGE<75)+3*(75<=PAT_AGE);

BAGE=0*(PAT_AGE<65)+1*(65<=PAT_AGE);

ADT_NEW_CAT=1*(ADT_MO<6)+2*(6<=ADT_MO<24)+3*(24<=ADT_MO);

CHA_NEW=1*(CHARLSON_1<=1)+2*(CHARLSON_1=2)+3*(CHARLSON_1=3)+4*(CHARLSON_1=4)+5*(5<=CHARLSON_1);

INSUP=1*(INSUP_TP_CD='4')+2*(INSUP_TP_CD='5' OR INSUP_TP_CD='7');

RUN;

DATA SHIM.DIS_1_DM_S_6;

SET SHIM.DIS_1_DM_S_5;

SAGE=0*(PAT_AGE<50)+1*(50<=PAT_AGE<60)+2*(60<=PAT_AGE<70)+3*(70<=PAT_AGE<80)+4*(80<=PAT_AGE);

NAGE=0*(PAT_AGE<55)+1*(55<=PAT_AGE<65)+2*(65<=PAT_AGE<75)+3*(75<=PAT_AGE);

BAGE=0*(PAT_AGE<65)+1*(65<=PAT_AGE);

ADT_NEW_CAT=1*(ADT_MO<6)+2*(6<=ADT_MO<24)+3*(24<=ADT_MO);

CHA_NEW=1*(CHARLSON_1<=1)+2*(CHARLSON_1=2)+3*(CHARLSON_1=3)+4*(CHARLSON_1=4)+5*(5<=CHARLSON_1);

INSUP=1*(INSUP_TP_CD='4')+2*(INSUP_TP_CD='5' OR INSUP_TP_CD='7');

RUN;

DATA SHIM.DIS_1_MI_S_6;

SET SHIM.DIS_1_MI_S_5;

SAGE=0*(PAT_AGE<50)+1*(50<=PAT_AGE<60)+2*(60<=PAT_AGE<70)+3*(70<=PAT_AGE<80)+4*(80<=PAT_AGE);

NAGE=0*(PAT_AGE<55)+1*(55<=PAT_AGE<65)+2*(65<=PAT_AGE<75)+3*(75<=PAT_AGE);

BAGE=0*(PAT_AGE<65)+1*(65<=PAT_AGE);

ADT_NEW_CAT=1*(ADT_MO<6)+2*(6<=ADT_MO<24)+3*(24<=ADT_MO);

CHA_NEW=1*(CHARLSON_1<=1)+2*(CHARLSON_1=2)+3*(CHARLSON_1=3)+4*(CHARLSON_1=4)+5*(5<=CHARLSON_1);

INSUP=1*(INSUP_TP_CD='4')+2*(INSUP_TP_CD='5' OR INSUP_TP_CD='7');

RUN;

DATA SHIM.DIS_2_ALZ_S_6;

SET SHIM.DIS_2_ALZ_S_5;

SAGE=0*(PAT_AGE<50)+1*(50<=PAT_AGE<60)+2*(60<=PAT_AGE<70)+3*(70<=PAT_AGE<80)+4*(80<=PAT_AGE);

NAGE=0*(PAT_AGE<55)+1*(55<=PAT_AGE<65)+2*(65<=PAT_AGE<75)+3*(75<=PAT_AGE);

BAGE=0*(PAT_AGE<65)+1*(65<=PAT_AGE);

ADT_NEW_CAT=1*(ADT_MO<6)+2*(6<=ADT_MO<24)+3*(24<=ADT_MO);

CHA_NEW=1*(CHARLSON_1<=1)+2*(CHARLSON_1=2)+3*(CHARLSON_1=3)+4*(CHARLSON_1=4)+5*(5<=CHARLSON_1);

IF DDX_IHD=1 OR DDX_MI=1 OR DDX_CVA=1 OR DDX_CHF=1 THEN CVD=1; ELSE CVD=0;

INSUP=1*(INSUP_TP_CD='4')+2*(INSUP_TP_CD='5' OR INSUP_TP_CD='7');

RUN;

DATA SHIM.DIS_2_PKS_S_6;

SET SHIM.DIS_2_PKS_S_5;

SAGE=0*(PAT_AGE<50)+1*(50<=PAT_AGE<60)+2*(60<=PAT_AGE<70)+3*(70<=PAT_AGE<80)+4*(80<=PAT_AGE);

NAGE=0*(PAT_AGE<55)+1*(55<=PAT_AGE<65)+2*(65<=PAT_AGE<75)+3*(75<=PAT_AGE);

BAGE=0*(PAT_AGE<65)+1*(65<=PAT_AGE);

ADT_NEW_CAT=1*(ADT_MO<6)+2*(6<=ADT_MO<24)+3*(24<=ADT_MO);

CHA_NEW=1*(CHARLSON_1<=1)+2*(CHARLSON_1=2)+3*(CHARLSON_1=3)+4*(CHARLSON_1=4)+5*(5<=CHARLSON_1);

IF DDX_IHD=1 OR DDX_MI=1 OR DDX_CVA=1 OR DDX_CHF=1 THEN CVD=1; ELSE CVD=0;

INSUP=1*(INSUP_TP_CD='4')+2*(INSUP_TP_CD='5' OR INSUP_TP_CD='7');

RUN;

*/

*=================================================;

*============ 기술적 분석 / 2018.03.30. ==============;

*=================================================;

*============CVA, DM, IHD, MI==================;

TITLE '=========================================

================= 기술적 분석 ===================

==============================================='

/*

PROC FREQ DATA=SHIM.DIS_1_CVA_S_6;

TABLES (

KWON

CITY

INSUP

TTX_CHOL

TTX_HTN

TTX_COA

TTX_PLT

TTX_ANT

DDX_HTN

DDX_LIV

DDX_CAN

DDX_CKD

DDX_COPD

DDX_ASTH

DDX_PVD

)*CASE / CHISQ;

RUN;

PROC FREQ DATA=SHIM.DIS_1_CVA_S_6;

TABLES (

PAT_AGE

SAGE

NAGE

BAGE

ADT_MO

ADT_NEW_CAT

CHARLSON_1

CHA_NEW

DIFFMO_2

)*CASE / CHISQ;

RUN;

PROC MEANS DATA=SHIM.DIS_1_CVA_S_6 N MEAN STD STDERR;

VAR PAT_AGE ADT_MO CHARLSON_1 ;

RUN;

PROC MEANS DATA=SHIM.DIS_1_CVA_S_6 N MEAN STD STDERR;

WHERE CASE=1;

VAR PAT_AGE ADT_MO CHARLSON_1;

RUN;

PROC MEANS DATA=SHIM.DIS_1_CVA_S_6 N MEAN STD STDERR;

WHERE CASE=2;

VAR PAT_AGE ADT_MO CHARLSON_1;

RUN;

*================ALZ, PKS====================;

PROC FREQ DATA=SHIM.DIS_2_ALZ_S_6;

TABLES (

KWON

CITY

INSUP

TTX_CHOL

TTX_HTN

TTX_COA

TTX_PLT

TTX_ANT

DDX_HTN

DDX_LIV

DDX_CAN

DDX_CKD

DDX_COPD

DDX_ASTH

DDX_PVD

DDX_DM

CVD

)*CASE / CHISQ;

RUN;

PROC FREQ DATA=SHIM.DIS_2_ALZ_S_6;

TABLES (

PAT_AGE

SAGE

NAGE

BAGE

ADT_MO

ADT_NEW_CAT

CHARLSON_1

CHA_NEW

DIFFMO_2

)*CASE / CHISQ;

RUN;

PROC MEANS DATA=SHIM.DIS_2_ALZ_S_6 N MEAN STD STDERR;

VAR PAT_AGE ADT_MO CHARLSON_1 ;

RUN;

PROC MEANS DATA=SHIM.DIS_2_ALZ_S_6 N MEAN STD STDERR;

WHERE CASE=1;

VAR PAT_AGE ADT_MO CHARLSON_1;

RUN;

PROC MEANS DATA=SHIM.DIS_2_ALZ_S_6 N MEAN STD STDERR;

WHERE CASE=2;

VAR PAT_AGE ADT_MO CHARLSON_1;

RUN;

*/

**PROC** **TTEST** DATA=SHIM.DIS_1_CVA_S_6; CLASS CASE; VAR PAT_AGE; **RUN**

**PROC** **TTEST** DATA=SHIM.DIS_1_CVA_S_6; CLASS CASE; VAR CHARLSON_1; **RUN**

**PROC** **TTEST** DATA=SHIM.DIS_2_ALZ_S_6; CLASS CASE; VAR PAT_AGE; **RUN**

**PROC** **TTEST** DATA=SHIM.DIS_2_ALZ_S_6; CLASS CASE; VAR CHARLSON_1; **RUN**

*=================================================;

*============ 분석적 분석 / 2018.03.30. ==============;

*=================================================;

TITLE '=========================================

================= 분석적 분석 ===================

==============================================='

TITLE '====================== CVA ========================'

**PROC** **LIFETEST** DATA=SHIM.DIS_1_CVA_S_6 OUTSURV=CVA_1;

TIME DIFFMO_2*CVA(**0**);

STRATA CASE;

**RUN**

**DATA** CVA_11;

SET CVA_1;

EVENT=**1**-SURVIVAL;

**RUN**

**PROC** **GPLOT** DATA=CVA_11;

PLOT EVENT*DIFFMO_2=CASE / HMINOR=**0**

**RUN**

**PROC** **PHREG** DATA=SHIM.DIS_1_CVA_S_6;

CLASS CASE;

MODEL DIFFMO_2*CVA(**0**)=CASE / RISKLIMITS

**RUN**

TITLE '====================== IHD ========================'

**PROC** **LIFETEST** DATA=SHIM.DIS_1_IHD_S_6 OUTSURV=IHD_1;

TIME DIFFMO_2*IHD(**0**);

STRATA CASE;

**RUN**

**DATA** IHD_11;

SET IHD_1;

EVENT=**1**-SURVIVAL;

**RUN**

**PROC** **GPLOT** DATA=IHD_11;

PLOT EVENT*DIFFMO_2=CASE / HMINOR=**0**

**RUN**

**PROC** **PHREG** DATA=SHIM.DIS_1_IHD_S_6;

CLASS CASE;

MODEL DIFFMO_2*IHD(**0**)=CASE / RISKLIMITS

**RUN**

TITLE '====================== DM ========================'

**PROC** **LIFETEST** DATA=SHIM.DIS_1_DM_S_6 OUTSURV=DM_1;

TIME DIFFMO_2*DM(**0**);

STRATA CASE;

**RUN**

**DATA** DM_11;

SET DM_1;

EVENT=**1**-SURVIVAL;

**RUN**

**PROC** **GPLOT** DATA=DM_11;

PLOT EVENT*DIFFMO_2=CASE / HMINOR=**0**

**RUN**

**PROC** **PHREG** DATA=SHIM.DIS_1_DM_S_6;

CLASS CASE;

MODEL DIFFMO_2*DM(**0**)=CASE / RISKLIMITS

**RUN**

TITLE '====================== MI ========================'

**PROC** **LIFETEST** DATA=SHIM.DIS_1_MI_S_6 OUTSURV=MI_1;

TIME DIFFMO_2*MI(**0**);

STRATA CASE;

**RUN**

**DATA** MI_11;

SET MI_1;

EVENT=**1**-SURVIVAL;

**RUN**

**PROC** **GPLOT** DATA=MI_11;

PLOT EVENT*DIFFMO_2=CASE / HMINOR=**0**

**RUN**

**PROC** **PHREG** DATA=SHIM.DIS_1_MI_S_6;

CLASS CASE;

MODEL DIFFMO_2*MI(**0**)=CASE / RISKLIMITS

**RUN**

TITLE '====================== ALZ ========================'

**PROC** **LIFETEST** DATA=SHIM.DIS_2_ALZ_S_6 OUTSURV=ALZ_1;

TIME DIFFMO_2*ALZ(**0**);

STRATA CASE;

**RUN**

**DATA** ALZ_11;

SET ALZ_1;

EVENT=**1**-SURVIVAL;

**RUN**

**PROC** **GPLOT** DATA=ALZ_11;

PLOT EVENT*DIFFMO_2=CASE / HMINOR=**0**

**RUN**

**PROC** **PHREG** DATA=SHIM.DIS_2_ALZ_S_6;

CLASS CASE;

MODEL DIFFMO_2*ALZ(**0**)=CASE / RISKLIMITS

**RUN**

TITLE '====================== PKS ========================'

**PROC** **LIFETEST** DATA=SHIM.DIS_2_PKS_S_6 OUTSURV=PKS_1;

TIME DIFFMO_2*PKS(**0**);

STRATA CASE;

**RUN**

**DATA** PKS_11;

SET PKS_1;

EVENT=**1**-SURVIVAL;

**RUN**

**PROC** **GPLOT** DATA=PKS_11;

PLOT EVENT*DIFFMO_2=CASE / HMINOR=**0**

**RUN**

**PROC** **PHREG** DATA=SHIM.DIS_2_PKS_S_6;

CLASS CASE;

MODEL DIFFMO_2*PKS(**0**)=CASE / RISKLIMITS

**RUN**

*==================================================================;

*========================== 공변수 분석 =============================;

*==================================================================;

TITLE '============================================================

========================== 공변수 분석 =============================

=================================================================='

TITLE '====================== CVA ========================'

**PROC** **PHREG** DATA=SHIM.DIS_1_CVA_S_6;

CLASS CASE;

MODEL DIFFMO_2*CVA(**0**)=PAT_AGE / RISKLIMITS

**RUN**

**PROC** **PHREG** DATA=SHIM.DIS_1_CVA_S_6;

CLASS CASE;

MODEL DIFFMO_2*CVA(**0**)=TTX_CHOL / RISKLIMITS

**RUN**

**PROC** **PHREG** DATA=SHIM.DIS_1_CVA_S_6;

CLASS CASE;

MODEL DIFFMO_2*CVA(**0**)=TTX_COA / RISKLIMITS

**RUN**

**PROC** **PHREG** DATA=SHIM.DIS_1_CVA_S_6;

CLASS CASE;

MODEL DIFFMO_2*CVA(**0**)=TTX_PLT / RISKLIMITS

**RUN**

**PROC** **PHREG** DATA=SHIM.DIS_1_CVA_S_6;

CLASS CASE;

MODEL DIFFMO_2*CVA(**0**)=TTX_ANT / RISKLIMITS

**RUN**

**PROC** **PHREG** DATA=SHIM.DIS_1_CVA_S_6;

CLASS CASE;

MODEL DIFFMO_2*CVA(**0**)=DDX_HTN / RISKLIMITS

**RUN**

**PROC** **PHREG** DATA=SHIM.DIS_1_CVA_S_6;

CLASS CASE;

MODEL DIFFMO_2*CVA(**0**)=DDX_LIV / RISKLIMITS

**RUN**

**PROC** **PHREG** DATA=SHIM.DIS_1_CVA_S_6;

CLASS CASE;

MODEL DIFFMO_2*CVA(**0**)=DDX_CKD / RISKLIMITS

**RUN**

**PROC** **PHREG** DATA=SHIM.DIS_1_CVA_S_6;

CLASS CASE;

MODEL DIFFMO_2*CVA(**0**)=DDX_COPD / RISKLIMITS

**RUN**

**PROC** **PHREG** DATA=SHIM.DIS_1_CVA_S_6;

CLASS CASE;

MODEL DIFFMO_2*CVA(**0**)=DDX_ASTH / RISKLIMITS

**RUN**

**PROC** **PHREG** DATA=SHIM.DIS_1_CVA_S_6;

CLASS CASE;

MODEL DIFFMO_2*CVA(**0**)=DDX_CAN / RISKLIMITS

**RUN**

**PROC** **PHREG** DATA=SHIM.DIS_1_CVA_S_6;

CLASS CASE;

MODEL DIFFMO_2*CVA(**0**)=CITY / RISKLIMITS

**RUN**

**PROC** **PHREG** DATA=SHIM.DIS_1_CVA_S_6;

CLASS CASE CHA_NEW(REF='1') ;

MODEL DIFFMO_2*CVA(**0**)=CHA_NEW / RISKLIMITS

**RUN**

**PROC** **PHREG** DATA=SHIM.DIS_1_CVA_S_6;

CLASS CASE;

MODEL DIFFMO_2*CVA(**0**)=INSUP / RISKLIMITS

**RUN**

TITLE '====================== IHD ========================'

**PROC** **PHREG** DATA=SHIM.DIS_1_IHD_S_6;

CLASS CASE;

MODEL DIFFMO_2*IHD(**0**)=PAT_AGE / RISKLIMITS

**RUN**

**PROC** **PHREG** DATA=SHIM.DIS_1_IHD_S_6;

CLASS CASE;

MODEL DIFFMO_2*IHD(**0**)=TTX_CHOL / RISKLIMITS

**RUN**

**PROC** **PHREG** DATA=SHIM.DIS_1_IHD_S_6;

CLASS CASE;

MODEL DIFFMO_2*IHD(**0**)=TTX_COA / RISKLIMITS

**RUN**

**PROC** **PHREG** DATA=SHIM.DIS_1_IHD_S_6;

CLASS CASE;

MODEL DIFFMO_2*IHD(**0**)=TTX_PLT / RISKLIMITS

**RUN**

**PROC** **PHREG** DATA=SHIM.DIS_1_IHD_S_6;

CLASS CASE;

MODEL DIFFMO_2*IHD(**0**)=TTX_ANT / RISKLIMITS

**RUN**

**PROC** **PHREG** DATA=SHIM.DIS_1_IHD_S_6;

CLASS CASE;

MODEL DIFFMO_2*IHD(**0**)=DDX_HTN / RISKLIMITS

**RUN**

**PROC** **PHREG** DATA=SHIM.DIS_1_IHD_S_6;

CLASS CASE;

MODEL DIFFMO_2*IHD(**0**)=DDX_LIV / RISKLIMITS

**RUN**

**PROC** **PHREG** DATA=SHIM.DIS_1_IHD_S_6;

CLASS CASE;

MODEL DIFFMO_2*IHD(**0**)=DDX_CKD / RISKLIMITS

**RUN**

**PROC** **PHREG** DATA=SHIM.DIS_1_IHD_S_6;

CLASS CASE;

MODEL DIFFMO_2*IHD(**0**)=DDX_COPD / RISKLIMITS

**RUN**

**PROC** **PHREG** DATA=SHIM.DIS_1_IHD_S_6;

CLASS CASE;

MODEL DIFFMO_2*IHD(**0**)=DDX_ASTH / RISKLIMITS

**RUN**

**PROC** **PHREG** DATA=SHIM.DIS_1_IHD_S_6;

CLASS CASE;

MODEL DIFFMO_2*IHD(**0**)=DDX_CAN / RISKLIMITS

**RUN**

**PROC** **PHREG** DATA=SHIM.DIS_1_IHD_S_6;

CLASS CASE;

MODEL DIFFMO_2*IHD(**0**)=CITY / RISKLIMITS

**RUN**

**PROC** **PHREG** DATA=SHIM.DIS_1_IHD_S_6;

CLASS CASE CHA_NEW(REF='1');

MODEL DIFFMO_2*IHD(**0**)=CHA_NEW / RISKLIMITS

**RUN**

**PROC** **PHREG** DATA=SHIM.DIS_1_IHD_S_6;

CLASS CASE;

MODEL DIFFMO_2*IHD(**0**)=INSUP / RISKLIMITS

**RUN**

TITLE '====================== DM ========================'

**PROC** **PHREG** DATA=SHIM.DIS_1_DM_S_6;

CLASS CASE;

MODEL DIFFMO_2*DM(**0**)=PAT_AGE / RISKLIMITS

**RUN**

**PROC** **PHREG** DATA=SHIM.DIS_1_DM_S_6;

CLASS CASE;

MODEL DIFFMO_2*DM(**0**)=TTX_CHOL / RISKLIMITS

**RUN**

**PROC** **PHREG** DATA=SHIM.DIS_1_DM_S_6;

CLASS CASE;

MODEL DIFFMO_2*DM(**0**)=TTX_COA / RISKLIMITS

**RUN**

**PROC** **PHREG** DATA=SHIM.DIS_1_DM_S_6;

CLASS CASE;

MODEL DIFFMO_2*DM(**0**)=TTX_PLT / RISKLIMITS

**RUN**

**PROC** **PHREG** DATA=SHIM.DIS_1_DM_S_6;

CLASS CASE;

MODEL DIFFMO_2*DM(**0**)=TTX_ANT / RISKLIMITS

**RUN**

**PROC** **PHREG** DATA=SHIM.DIS_1_DM_S_6;

CLASS CASE;

MODEL DIFFMO_2*DM(**0**)=DDX_HTN / RISKLIMITS

**RUN**

**PROC** **PHREG** DATA=SHIM.DIS_1_DM_S_6;

CLASS CASE;

MODEL DIFFMO_2*DM(**0**)=DDX_LIV / RISKLIMITS

**RUN**

**PROC** **PHREG** DATA=SHIM.DIS_1_DM_S_6;

CLASS CASE;

MODEL DIFFMO_2*DM(**0**)=DDX_CKD / RISKLIMITS

**RUN**

**PROC** **PHREG** DATA=SHIM.DIS_1_DM_S_6;

CLASS CASE;

MODEL DIFFMO_2*DM(**0**)=DDX_COPD / RISKLIMITS

**RUN**

**PROC** **PHREG** DATA=SHIM.DIS_1_DM_S_6;

CLASS CASE;

MODEL DIFFMO_2*DM(**0**)=DDX_ASTH / RISKLIMITS

**RUN**

**PROC** **PHREG** DATA=SHIM.DIS_1_DM_S_6;

CLASS CASE;

MODEL DIFFMO_2*DM(**0**)=DDX_CAN / RISKLIMITS

**RUN**

**PROC** **PHREG** DATA=SHIM.DIS_1_DM_S_6;

CLASS CASE;

MODEL DIFFMO_2*DM(**0**)=CITY / RISKLIMITS

**RUN**

**PROC** **PHREG** DATA=SHIM.DIS_1_DM_S_6;

CLASS CASE CHA_NEW(REF='1');

MODEL DIFFMO_2*DM(**0**)=CHA_NEW / RISKLIMITS

**RUN**

**PROC** **PHREG** DATA=SHIM.DIS_1_DM_S_6;

CLASS CASE;

MODEL DIFFMO_2*DM(**0**)=INSUP / RISKLIMITS

**RUN**

TITLE '====================== MI ========================'

**PROC** **PHREG** DATA=SHIM.DIS_1_MI_S_6;

CLASS CASE;

MODEL DIFFMO_2*MI(**0**)=PAT_AGE / RISKLIMITS

**RUN**

**PROC** **PHREG** DATA=SHIM.DIS_1_MI_S_6;

CLASS CASE;

MODEL DIFFMO_2*MI(**0**)=TTX_CHOL / RISKLIMITS

**RUN**

**PROC** **PHREG** DATA=SHIM.DIS_1_MI_S_6;

CLASS CASE;

MODEL DIFFMO_2*MI(**0**)=TTX_COA / RISKLIMITS

**RUN**

**PROC** **PHREG** DATA=SHIM.DIS_1_MI_S_6;

CLASS CASE;

MODEL DIFFMO_2*MI(**0**)=TTX_PLT / RISKLIMITS

**RUN**

**PROC** **PHREG** DATA=SHIM.DIS_1_MI_S_6;

CLASS CASE;

MODEL DIFFMO_2*MI(**0**)=TTX_ANT / RISKLIMITS

**RUN**

**PROC** **PHREG** DATA=SHIM.DIS_1_MI_S_6;

CLASS CASE;

MODEL DIFFMO_2*MI(**0**)=DDX_HTN / RISKLIMITS

**RUN**

**PROC** **PHREG** DATA=SHIM.DIS_1_MI_S_6;

CLASS CASE;

MODEL DIFFMO_2*MI(**0**)=DDX_LIV / RISKLIMITS

**RUN**

**PROC** **PHREG** DATA=SHIM.DIS_1_MI_S_6;

CLASS CASE;

MODEL DIFFMO_2*MI(**0**)=DDX_CKD / RISKLIMITS

**RUN**

**PROC** **PHREG** DATA=SHIM.DIS_1_MI_S_6;

CLASS CASE;

MODEL DIFFMO_2*MI(**0**)=DDX_COPD / RISKLIMITS

**RUN**

**PROC** **PHREG** DATA=SHIM.DIS_1_MI_S_6;

CLASS CASE;

MODEL DIFFMO_2*MI(**0**)=DDX_ASTH / RISKLIMITS

**RUN**

**PROC** **PHREG** DATA=SHIM.DIS_1_MI_S_6;

CLASS CASE;

MODEL DIFFMO_2*MI(**0**)=DDX_CAN / RISKLIMITS

**RUN**

**PROC** **PHREG** DATA=SHIM.DIS_1_MI_S_6;

CLASS CASE;

MODEL DIFFMO_2*MI(**0**)=CITY / RISKLIMITS

**RUN**

**PROC** **PHREG** DATA=SHIM.DIS_1_MI_S_6;

CLASS CASE CHA_NEW(REF='1');

MODEL DIFFMO_2*MI(**0**)=CHA_NEW / RISKLIMITS

**RUN**

**PROC** **PHREG** DATA=SHIM.DIS_1_MI_S_6;

CLASS CASE;

MODEL DIFFMO_2*MI(**0**)=INSUP / RISKLIMITS

**RUN**

TITLE '====================== ALZ ========================'

**PROC** **PHREG** DATA=SHIM.DIS_2_ALZ_S_6;

CLASS CASE;

MODEL DIFFMO_2*ALZ(**0**)=PAT_AGE / RISKLIMITS

**RUN**

**PROC** **PHREG** DATA=SHIM.DIS_2_ALZ_S_6;

CLASS CASE;

MODEL DIFFMO_2*ALZ(**0**)=TTX_CHOL / RISKLIMITS

**RUN**

**PROC** **PHREG** DATA=SHIM.DIS_2_ALZ_S_6;

CLASS CASE;

MODEL DIFFMO_2*ALZ(**0**)=TTX_COA / RISKLIMITS

**RUN**

**PROC** **PHREG** DATA=SHIM.DIS_2_ALZ_S_6;

CLASS CASE;

MODEL DIFFMO_2*ALZ(**0**)=TTX_PLT / RISKLIMITS

**RUN**

**PROC** **PHREG** DATA=SHIM.DIS_2_ALZ_S_6;

CLASS CASE;

MODEL DIFFMO_2*ALZ(**0**)=TTX_ANT / RISKLIMITS

**RUN**

**PROC** **PHREG** DATA=SHIM.DIS_2_ALZ_S_6;

CLASS CASE;

MODEL DIFFMO_2*ALZ(**0**)=DDX_HTN / RISKLIMITS

**RUN**

**PROC** **PHREG** DATA=SHIM.DIS_2_ALZ_S_6;

CLASS CASE;

MODEL DIFFMO_2*ALZ(**0**)=DDX_DM / RISKLIMITS

**RUN**

**PROC** **PHREG** DATA=SHIM.DIS_2_ALZ_S_6;

CLASS CASE;

MODEL DIFFMO_2*ALZ(**0**)=CVD / RISKLIMITS

**RUN**

**PROC** **PHREG** DATA=SHIM.DIS_2_ALZ_S_6;

CLASS CASE;

MODEL DIFFMO_2*ALZ(**0**)=DDX_LIV / RISKLIMITS

**RUN**

**PROC** **PHREG** DATA=SHIM.DIS_2_ALZ_S_6;

CLASS CASE;

MODEL DIFFMO_2*ALZ(**0**)=DDX_CKD / RISKLIMITS

**RUN**

**PROC** **PHREG** DATA=SHIM.DIS_2_ALZ_S_6;

CLASS CASE;

MODEL DIFFMO_2*ALZ(**0**)=DDX_COPD / RISKLIMITS

**RUN**

**PROC** **PHREG** DATA=SHIM.DIS_2_ALZ_S_6;

CLASS CASE;

MODEL DIFFMO_2*ALZ(**0**)=DDX_ASTH / RISKLIMITS

**RUN**

**PROC** **PHREG** DATA=SHIM.DIS_2_ALZ_S_6;

CLASS CASE;

MODEL DIFFMO_2*ALZ(**0**)=DDX_CAN / RISKLIMITS

**RUN**

**PROC** **PHREG** DATA=SHIM.DIS_2_ALZ_S_6;

CLASS CASE;

MODEL DIFFMO_2*ALZ(**0**)=CITY / RISKLIMITS

**RUN**

**PROC** **PHREG** DATA=SHIM.DIS_2_ALZ_S_6;

CLASS CASE CHA_NEW(REF='1');

MODEL DIFFMO_2*ALZ(**0**)=CHA_NEW / RISKLIMITS

**RUN**

**PROC** **PHREG** DATA=SHIM.DIS_2_ALZ_S_6;

CLASS CASE;

MODEL DIFFMO_2*ALZ(**0**)=INSUP / RISKLIMITS

**RUN**

TITLE '====================== PKS ========================'

**PROC** **PHREG** DATA=SHIM.DIS_2_PKS_S_6;

CLASS CASE;

MODEL DIFFMO_2*PKS(**0**)=PAT_AGE / RISKLIMITS

**RUN**

**PROC** **PHREG** DATA=SHIM.DIS_2_PKS_S_6;

CLASS CASE;

MODEL DIFFMO_2*PKS(**0**)=TTX_CHOL / RISKLIMITS

**RUN**

**PROC** **PHREG** DATA=SHIM.DIS_2_PKS_S_6;

CLASS CASE;

MODEL DIFFMO_2*PKS(**0**)=TTX_COA / RISKLIMITS

**RUN**

**PROC** **PHREG** DATA=SHIM.DIS_2_PKS_S_6;

CLASS CASE;

MODEL DIFFMO_2*PKS(**0**)=TTX_PLT / RISKLIMITS

**RUN**

**PROC** **PHREG** DATA=SHIM.DIS_2_PKS_S_6;

CLASS CASE;

MODEL DIFFMO_2*PKS(**0**)=TTX_ANT / RISKLIMITS

**RUN**

**PROC** **PHREG** DATA=SHIM.DIS_2_PKS_S_6;

CLASS CASE;

MODEL DIFFMO_2*PKS(**0**)=DDX_HTN / RISKLIMITS

**RUN**

**PROC** **PHREG** DATA=SHIM.DIS_2_PKS_S_6;

CLASS CASE;

MODEL DIFFMO_2*PKS(**0**)=DDX_DM / RISKLIMITS

**RUN**

**PROC** **PHREG** DATA=SHIM.DIS_2_PKS_S_6;

CLASS CASE;

MODEL DIFFMO_2*PKS(**0**)=CVD / RISKLIMITS

**RUN**

**PROC** **PHREG** DATA=SHIM.DIS_2_PKS_S_6;

CLASS CASE;

MODEL DIFFMO_2*PKS(**0**)=DDX_LIV / RISKLIMITS

**RUN**

**PROC** **PHREG** DATA=SHIM.DIS_2_PKS_S_6;

CLASS CASE;

MODEL DIFFMO_2*PKS(**0**)=DDX_CKD / RISKLIMITS

**RUN**

**PROC** **PHREG** DATA=SHIM.DIS_2_PKS_S_6;

CLASS CASE;

MODEL DIFFMO_2*PKS(**0**)=DDX_COPD / RISKLIMITS

**RUN**

**PROC** **PHREG** DATA=SHIM.DIS_2_PKS_S_6;

CLASS CASE;

MODEL DIFFMO_2*PKS(**0**)=DDX_ASTH / RISKLIMITS

**RUN**

**PROC** **PHREG** DATA=SHIM.DIS_2_PKS_S_6;

CLASS CASE;

MODEL DIFFMO_2*PKS(**0**)=DDX_CAN / RISKLIMITS

**RUN**

**PROC** **PHREG** DATA=SHIM.DIS_2_PKS_S_6;

CLASS CASE;

MODEL DIFFMO_2*PKS(**0**)=CITY / RISKLIMITS

**RUN**

**PROC** **PHREG** DATA=SHIM.DIS_2_PKS_S_6;

CLASS CASE CHA_NEW(REF='1');

MODEL DIFFMO_2*PKS(**0**)=CHA_NEW / RISKLIMITS

**RUN**

**PROC** **PHREG** DATA=SHIM.DIS_2_PKS_S_6;

CLASS CASE;

MODEL DIFFMO_2*PKS(**0**)=INSUP / RISKLIMITS

**RUN**

TITLE '==================================================

==============MULTIVAIRATE ANALYSIS====================

================MULTI-COLLINEARITY ====================

========================================================'

*========== MULTI-COLLINEARITY =========;

**PROC** **CORR** DATA=SHIM.DIS_1_CVA_S_6 SPEARMAN

VAR CASE PAT_AGE TTX_ANT DDX_HTN DDX_COPD CHA_NEW INSUP;

**RUN**

**PROC** **PHREG** DATA=SHIM.DIS_1_CVA_S_6;

CLASS CASE CHA_NEW(REF='1');

MODEL DIFFMO_2*CVA(**0**)=CASE PAT_AGE TTX_ANT DDX_HTN DDX_COPD INSUP / RISKLIMITS

**RUN**

**PROC** **CORR** DATA=SHIM.DIS_1_IHD_S_6 SPEARMAN

VAR PAT_AGE TTX_CHOL TTX_PLT TTX_ANT DDX_HTN DDX_COPD CITY CHA_NEW INSUP;

**RUN**

**PROC** **PHREG** DATA=SHIM.DIS_1_IHD_S_6;

CLASS CASE CHA_NEW(REF='1');

MODEL DIFFMO_2*IHD(**0**)=PAT_AGE TTX_CHOL TTX_PLT TTX_ANT DDX_HTN DDX_COPD CITY INSUP / RISKLIMITS

**RUN**

**PROC** **CORR** DATA=SHIM.DIS_1_DM_S_6 SPEARMAN

VAR PAT_AGE TTX_PLT DDX_HTN DDX_LIV CHA_NEW INSUP;

**RUN**

**PROC** **PHREG** DATA=SHIM.DIS_1_DM_S_6;

CLASS CASE CHA_NEW(REF='1');

MODEL DIFFMO_2*DM(**0**)=PAT_AGE TTX_PLT DDX_HTN DDX_LIV INSUP / RISKLIMITS

**RUN**

**PROC** **CORR** DATA=SHIM.DIS_1_MI_S_6 SPEARMAN

VAR PAT_AGE CHA_NEW;

**RUN**

**PROC** **PHREG** DATA=SHIM.DIS_1_MI_S_6;

CLASS CASE CHA_NEW(REF='1');

MODEL DIFFMO_2*MI(**0**)=PAT_AGE / RISKLIMITS

**RUN**

**PROC** **CORR** DATA=SHIM.DIS_2_ALZ_S_6 SPEARMAN

VAR CASE PAT_AGE TTX_COA TTX_PLT TTX_ANT DDX_DM CVD DDX_COPD DDX_ASTH INSUP;

**RUN**

**PROC** **PHREG** DATA=SHIM.DIS_2_ALZ_S_6;

CLASS CASE;

MODEL DIFFMO_2*ALZ(**0**)=CASE PAT_AGE TTX_COA TTX_PLT TTX_ANT DDX_DM CVD DDX_COPD DDX_ASTH INSUP / RISKLIMITS

**RUN**

**PROC** **CORR** DATA=SHIM.DIS_2_PKS_S_6 SPEARMAN

VAR PAT_AGE CVD CITY CHA_NEW;

**RUN**

**PROC** **PHREG** DATA=SHIM.DIS_2_PKS_S_6;

CLASS CASE CHA_NEW(REF='1');

MODEL DIFFMO_2*PKS(**0**)=PAT_AGE CVD CITY / RISKLIMITS

**RUN**

/*

DATA SHIM.DIS_NEWADT;

SET SHIM.DIS_1_CVA_S_6;

IF CASE=2 THEN NEW_GROUP=1;

IF CASE=1 AND ADT_NEW_CAT=1 THEN NEW_GROUP=2;

IF CASE=1 AND ADT_NEW_CAT=2 THEN NEW_GROUP=3;

IF CASE=1 AND ADT_NEW_CAT=3 THEN NEW_GROUP=4;

RUN;

PROC PHREG DATA=SHIM.DIS_NEWADT;

CLASS NEW_GROUP(REF='1');

MODEL DIFFMO_2*CVA(0)=NEW_GROUP / RISKLIMITS;

RUN;

PROC PHREG DATA=SHIM.DIS_NEWADT;

CLASS NEW_GROUP(REF='1');

MODEL DIFFMO_2*CVA(0)=NEW_GROUP PAT_AGE TTX_ANT DDX_HTN DDX_COPD INSUP / RISKLIMITS;

RUN;

DATA SHIM.DIS_NEWADT_1;

SET SHIM.DIS_2_ALZ_S_6;

IF CASE=2 THEN NEW_GROUP=1;

IF CASE=1 AND ADT_NEW_CAT=1 THEN NEW_GROUP=2;

IF CASE=1 AND ADT_NEW_CAT=2 THEN NEW_GROUP=3;

IF CASE=1 AND ADT_NEW_CAT=3 THEN NEW_GROUP=4;

RUN;

PROC PHREG DATA=SHIM.DIS_NEWADT_1;

CLASS NEW_GROUP(REF='1');

MODEL DIFFMO_2*ALZ(0)=NEW_GROUP / RISKLIMITS;

RUN;

PROC PHREG DATA=SHIM.DIS_NEWADT_1;

CLASS NEW_GROUP(REF='1');

MODEL DIFFMO_2*ALZ(0)=NEW_GROUP PAT_AGE TTX_COA TTX_PLT TTX_ANT DDX_DM CVD DDX_COPD DDX_ASTH INSUP / RISKLIMITS;

RUN;

*/

* ============================================================================;

*======================SUB-GROUPING : SAGE/ NAGE/ BAGE====================;

*============================================================================';

/*

DATA SHIM.DIS_1_CVA_S_7_S0; SET SHIM.DIS_1_CVA_S_6; IF SAGE=0; RUN;

DATA SHIM.DIS_1_IHD_S_7_S0; SET SHIM.DIS_1_IHD_S_6; IF SAGE=0; RUN;

DATA SHIM.DIS_1_DM_S_7_S0; SET SHIM.DIS_1_DM_S_6; IF SAGE=0; RUN;

DATA SHIM.DIS_1_MI_S_7_S0; SET SHIM.DIS_1_MI_S_6; IF SAGE=0; RUN;

DATA SHIM.DIS_2_ALZ_S_7_S0; SET SHIM.DIS_2_ALZ_S_6; IF SAGE=0; RUN;

DATA SHIM.DIS_2_PKS_S_7_S0; SET SHIM.DIS_2_PKS_S_6; IF SAGE=0; RUN;

DATA SHIM.DIS_1_CVA_S_7_S1; SET SHIM.DIS_1_CVA_S_6; IF SAGE=1; RUN;

DATA SHIM.DIS_1_IHD_S_7_S1; SET SHIM.DIS_1_IHD_S_6; IF SAGE=1; RUN;

DATA SHIM.DIS_1_DM_S_7_S1; SET SHIM.DIS_1_DM_S_6; IF SAGE=1; RUN;

DATA SHIM.DIS_1_MI_S_7_S1; SET SHIM.DIS_1_MI_S_6; IF SAGE=1; RUN;

DATA SHIM.DIS_2_ALZ_S_7_S1; SET SHIM.DIS_2_ALZ_S_6; IF SAGE=1; RUN;

DATA SHIM.DIS_2_PKS_S_7_S1; SET SHIM.DIS_2_PKS_S_6; IF SAGE=1; RUN;

DATA SHIM.DIS_1_CVA_S_7_S2; SET SHIM.DIS_1_CVA_S_6; IF SAGE=2; RUN;

DATA SHIM.DIS_1_IHD_S_7_S2; SET SHIM.DIS_1_IHD_S_6; IF SAGE=2; RUN;

DATA SHIM.DIS_1_DM_S_7_S2; SET SHIM.DIS_1_DM_S_6; IF SAGE=2; RUN;

DATA SHIM.DIS_1_MI_S_7_S2; SET SHIM.DIS_1_MI_S_6; IF SAGE=2; RUN;

DATA SHIM.DIS_2_ALZ_S_7_S2; SET SHIM.DIS_2_ALZ_S_6; IF SAGE=2; RUN;

DATA SHIM.DIS_2_PKS_S_7_S2; SET SHIM.DIS_2_PKS_S_6; IF SAGE=2; RUN;

DATA SHIM.DIS_1_CVA_S_7_S3; SET SHIM.DIS_1_CVA_S_6; IF SAGE=3; RUN;

DATA SHIM.DIS_1_IHD_S_7_S3; SET SHIM.DIS_1_IHD_S_6; IF SAGE=3; RUN;

DATA SHIM.DIS_1_DM_S_7_S3; SET SHIM.DIS_1_DM_S_6; IF SAGE=3; RUN;

DATA SHIM.DIS_1_MI_S_7_S3; SET SHIM.DIS_1_MI_S_6; IF SAGE=3; RUN;

DATA SHIM.DIS_2_ALZ_S_7_S3; SET SHIM.DIS_2_ALZ_S_6; IF SAGE=3; RUN;

DATA SHIM.DIS_2_PKS_S_7_S3; SET SHIM.DIS_2_PKS_S_6; IF SAGE=3; RUN;

DATA SHIM.DIS_1_CVA_S_7_S4; SET SHIM.DIS_1_CVA_S_6; IF SAGE=4; RUN;

DATA SHIM.DIS_1_IHD_S_7_S4; SET SHIM.DIS_1_IHD_S_6; IF SAGE=4; RUN;

DATA SHIM.DIS_1_DM_S_7_S4; SET SHIM.DIS_1_DM_S_6; IF SAGE=4; RUN;

DATA SHIM.DIS_1_MI_S_7_S4; SET SHIM.DIS_1_MI_S_6; IF SAGE=4; RUN;

DATA SHIM.DIS_2_ALZ_S_7_S4; SET SHIM.DIS_2_ALZ_S_6; IF SAGE=4; RUN;

DATA SHIM.DIS_2_PKS_S_7_S4; SET SHIM.DIS_2_PKS_S_6; IF SAGE=4; RUN;

*/

/*

DATA SHIM.DIS_1_CVA_S_7_N0; SET SHIM.DIS_1_CVA_S_6; IF NAGE=0; RUN;

DATA SHIM.DIS_1_IHD_S_7_N0; SET SHIM.DIS_1_IHD_S_6; IF NAGE=0; RUN;

DATA SHIM.DIS_1_DM_S_7_N0; SET SHIM.DIS_1_DM_S_6; IF NAGE=0; RUN;

DATA SHIM.DIS_1_MI_S_7_N0; SET SHIM.DIS_1_MI_S_6; IF NAGE=0; RUN;

DATA SHIM.DIS_2_ALZ_S_7_N0; SET SHIM.DIS_2_ALZ_S_6; IF NAGE=0; RUN;

DATA SHIM.DIS_2_PKS_S_7_N0; SET SHIM.DIS_2_PKS_S_6; IF NAGE=0; RUN;

DATA SHIM.DIS_1_CVA_S_7_N1; SET SHIM.DIS_1_CVA_S_6; IF NAGE=1; RUN;

DATA SHIM.DIS_1_IHD_S_7_N1; SET SHIM.DIS_1_IHD_S_6; IF NAGE=1; RUN;

DATA SHIM.DIS_1_DM_S_7_N1; SET SHIM.DIS_1_DM_S_6; IF NAGE=1; RUN;

DATA SHIM.DIS_1_MI_S_7_N1; SET SHIM.DIS_1_MI_S_6; IF NAGE=1; RUN;

DATA SHIM.DIS_2_ALZ_S_7_N1; SET SHIM.DIS_2_ALZ_S_6; IF NAGE=1; RUN;

DATA SHIM.DIS_2_PKS_S_7_N1; SET SHIM.DIS_2_PKS_S_6; IF NAGE=1; RUN;

DATA SHIM.DIS_1_CVA_S_7_N2; SET SHIM.DIS_1_CVA_S_6; IF NAGE=2; RUN;

DATA SHIM.DIS_1_IHD_S_7_N2; SET SHIM.DIS_1_IHD_S_6; IF NAGE=2; RUN;

DATA SHIM.DIS_1_DM_S_7_N2; SET SHIM.DIS_1_DM_S_6; IF NAGE=2; RUN;

DATA SHIM.DIS_1_MI_S_7_N2; SET SHIM.DIS_1_MI_S_6; IF NAGE=2; RUN;

DATA SHIM.DIS_2_ALZ_S_7_N2; SET SHIM.DIS_2_ALZ_S_6; IF NAGE=2; RUN;

DATA SHIM.DIS_2_PKS_S_7_N2; SET SHIM.DIS_2_PKS_S_6; IF NAGE=2; RUN;

DATA SHIM.DIS_1_CVA_S_7_N3; SET SHIM.DIS_1_CVA_S_6; IF NAGE=3; RUN;

DATA SHIM.DIS_1_IHD_S_7_N3; SET SHIM.DIS_1_IHD_S_6; IF NAGE=3; RUN;

DATA SHIM.DIS_1_DM_S_7_N3; SET SHIM.DIS_1_DM_S_6; IF NAGE=3; RUN;

DATA SHIM.DIS_1_MI_S_7_N3; SET SHIM.DIS_1_MI_S_6; IF NAGE=3; RUN;

DATA SHIM.DIS_2_ALZ_S_7_N3; SET SHIM.DIS_2_ALZ_S_6; IF NAGE=3; RUN;

DATA SHIM.DIS_2_PKS_S_7_N3; SET SHIM.DIS_2_PKS_S_6; IF NAGE=3; RUN;

*/

/*

DATA SHIM.DIS_1_CVA_S_7_B1; SET SHIM.DIS_1_CVA_S_6; IF BAGE=1; RUN;

DATA SHIM.DIS_1_IHD_S_7_B1; SET SHIM.DIS_1_IHD_S_6; IF BAGE=1; RUN;

DATA SHIM.DIS_1_DM_S_7_B1; SET SHIM.DIS_1_DM_S_6; IF BAGE=1; RUN;

DATA SHIM.DIS_1_MI_S_7_B1; SET SHIM.DIS_1_MI_S_6; IF BAGE=1; RUN;

DATA SHIM.DIS_2_ALZ_S_7_B1; SET SHIM.DIS_2_ALZ_S_6; IF BAGE=1; RUN;

DATA SHIM.DIS_2_PKS_S_7_B1; SET SHIM.DIS_2_PKS_S_6; IF BAGE=1; RUN;

DATA SHIM.DIS_1_CVA_S_7_B0; SET SHIM.DIS_1_CVA_S_6; IF BAGE=0; RUN;

DATA SHIM.DIS_1_IHD_S_7_B0; SET SHIM.DIS_1_IHD_S_6; IF BAGE=0; RUN;

DATA SHIM.DIS_1_DM_S_7_B0; SET SHIM.DIS_1_DM_S_6; IF BAGE=0; RUN;

DATA SHIM.DIS_1_MI_S_7_B0; SET SHIM.DIS_1_MI_S_6; IF BAGE=0; RUN;

DATA SHIM.DIS_2_ALZ_S_7_B0; SET SHIM.DIS_2_ALZ_S_6; IF BAGE=0; RUN;

DATA SHIM.DIS_2_PKS_S_7_B0; SET SHIM.DIS_2_PKS_S_6; IF BAGE=0; RUN;

*/

*===============================================;

*===============================================;

*===============================================;

/*

TITLE '=======================================================================

*================SUB-GROUP ANALYSIS : SAGE/ NAGE/ BAGE====================

*============================================================================';

TITLE '*****************************SAGE = 0 ***************************';

TITLE '====================== CVA ========================';

PROC PHREG DATA=SHIM.DIS_1_CVA_S_7_S0; CLASS CASE; MODEL DIFFMO_2*CVA(0)=CASE / RISKLIMITS; RUN;

PROC PHREG DATA=SHIM.DIS_1_CVA_S_7_S0; CLASS CASE; MODEL DIFFMO_2*CVA(0)=TTX_CHOL / RISKLIMITS; RUN;

PROC PHREG DATA=SHIM.DIS_1_CVA_S_7_S0; CLASS CASE; MODEL DIFFMO_2*CVA(0)=TTX_COA / RISKLIMITS; RUN;

PROC PHREG DATA=SHIM.DIS_1_CVA_S_7_S0; CLASS CASE; MODEL DIFFMO_2*CVA(0)=TTX_PLT / RISKLIMITS; RUN;

PROC PHREG DATA=SHIM.DIS_1_CVA_S_7_S0; CLASS CASE; MODEL DIFFMO_2*CVA(0)=TTX_ANT / RISKLIMITS; RUN;

PROC PHREG DATA=SHIM.DIS_1_CVA_S_7_S0; CLASS CASE; MODEL DIFFMO_2*CVA(0)=DDX_HTN / RISKLIMITS; RUN;

PROC PHREG DATA=SHIM.DIS_1_CVA_S_7_S0; CLASS CASE; MODEL DIFFMO_2*CVA(0)=DDX_LIV / RISKLIMITS; RUN;

PROC PHREG DATA=SHIM.DIS_1_CVA_S_7_S0; CLASS CASE; MODEL DIFFMO_2*CVA(0)=DDX_CKD / RISKLIMITS; RUN;

PROC PHREG DATA=SHIM.DIS_1_CVA_S_7_S0; CLASS CASE; MODEL DIFFMO_2*CVA(0)=DDX_COPD / RISKLIMITS; RUN;

PROC PHREG DATA=SHIM.DIS_1_CVA_S_7_S0; CLASS CASE; MODEL DIFFMO_2*CVA(0)=DDX_ASTH / RISKLIMITS; RUN;

PROC PHREG DATA=SHIM.DIS_1_CVA_S_7_S0; CLASS CASE; MODEL DIFFMO_2*CVA(0)=DDX_CAN / RISKLIMITS; RUN;

PROC PHREG DATA=SHIM.DIS_1_CVA_S_7_S0; CLASS CASE; MODEL DIFFMO_2*CVA(0)=CITY / RISKLIMITS; RUN;

PROC PHREG DATA=SHIM.DIS_1_CVA_S_7_S0; CLASS CASE CHA_NEW(REF='1'); MODEL DIFFMO_2*CVA(0)=CHA_NEW / RISKLIMITS; RUN;

PROC PHREG DATA=SHIM.DIS_1_CVA_S_7_S0; CLASS CASE; MODEL DIFFMO_2*CVA(0)=INSUP / RISKLIMITS; RUN;

TITLE '====================== IHD ========================';

PROC PHREG DATA=SHIM.DIS_1_IHD_S_7_S0; CLASS CASE; MODEL DIFFMO_2*IHD(0)=CASE / RISKLIMITS; RUN;

PROC PHREG DATA=SHIM.DIS_1_IHD_S_7_S0; CLASS CASE; MODEL DIFFMO_2*IHD(0)=TTX_CHOL / RISKLIMITS; RUN;

PROC PHREG DATA=SHIM.DIS_1_IHD_S_7_S0; CLASS CASE; MODEL DIFFMO_2*IHD(0)=TTX_COA / RISKLIMITS; RUN;

PROC PHREG DATA=SHIM.DIS_1_IHD_S_7_S0; CLASS CASE; MODEL DIFFMO_2*IHD(0)=TTX_PLT / RISKLIMITS; RUN;

PROC PHREG DATA=SHIM.DIS_1_IHD_S_7_S0; CLASS CASE; MODEL DIFFMO_2*IHD(0)=TTX_ANT / RISKLIMITS; RUN;

PROC PHREG DATA=SHIM.DIS_1_IHD_S_7_S0; CLASS CASE; MODEL DIFFMO_2*IHD(0)=DDX_HTN / RISKLIMITS; RUN;

PROC PHREG DATA=SHIM.DIS_1_IHD_S_7_S0; CLASS CASE; MODEL DIFFMO_2*IHD(0)=DDX_LIV / RISKLIMITS; RUN;

PROC PHREG DATA=SHIM.DIS_1_IHD_S_7_S0; CLASS CASE; MODEL DIFFMO_2*IHD(0)=DDX_CKD / RISKLIMITS; RUN;

PROC PHREG DATA=SHIM.DIS_1_IHD_S_7_S0; CLASS CASE; MODEL DIFFMO_2*IHD(0)=DDX_COPD / RISKLIMITS; RUN;

PROC PHREG DATA=SHIM.DIS_1_IHD_S_7_S0; CLASS CASE; MODEL DIFFMO_2*IHD(0)=DDX_ASTH / RISKLIMITS; RUN;

PROC PHREG DATA=SHIM.DIS_1_IHD_S_7_S0; CLASS CASE; MODEL DIFFMO_2*IHD(0)=DDX_CAN / RISKLIMITS; RUN;

PROC PHREG DATA=SHIM.DIS_1_IHD_S_7_S0; CLASS CASE; MODEL DIFFMO_2*IHD(0)=CITY / RISKLIMITS; RUN;

PROC PHREG DATA=SHIM.DIS_1_IHD_S_7_S0; CLASS CASE CHA_NEW(REF='1'); MODEL DIFFMO_2*IHD(0)=CHA_NEW / RISKLIMITS; RUN;

PROC PHREG DATA=SHIM.DIS_1_IHD_S_7_S0; CLASS CASE; MODEL DIFFMO_2*IHD(0)=INSUP / RISKLIMITS; RUN;

TITLE '====================== DM ========================';

PROC PHREG DATA=SHIM.DIS_1_DM_S_7_S0; CLASS CASE; MODEL DIFFMO_2*DM(0)=CASE / RISKLIMITS; RUN;

PROC PHREG DATA=SHIM.DIS_1_DM_S_7_S0; CLASS CASE; MODEL DIFFMO_2*DM(0)=TTX_CHOL / RISKLIMITS; RUN;

PROC PHREG DATA=SHIM.DIS_1_DM_S_7_S0; CLASS CASE; MODEL DIFFMO_2*DM(0)=TTX_COA / RISKLIMITS; RUN;

PROC PHREG DATA=SHIM.DIS_1_DM_S_7_S0; CLASS CASE; MODEL DIFFMO_2*DM(0)=TTX_PLT / RISKLIMITS; RUN;

PROC PHREG DATA=SHIM.DIS_1_DM_S_7_S0; CLASS CASE; MODEL DIFFMO_2*DM(0)=TTX_ANT / RISKLIMITS; RUN;

PROC PHREG DATA=SHIM.DIS_1_DM_S_7_S0; CLASS CASE; MODEL DIFFMO_2*DM(0)=DDX_HTN / RISKLIMITS; RUN;

PROC PHREG DATA=SHIM.DIS_1_DM_S_7_S0; CLASS CASE; MODEL DIFFMO_2*DM(0)=DDX_LIV / RISKLIMITS; RUN;

PROC PHREG DATA=SHIM.DIS_1_DM_S_7_S0; CLASS CASE; MODEL DIFFMO_2*DM(0)=DDX_CKD / RISKLIMITS; RUN;

PROC PHREG DATA=SHIM.DIS_1_DM_S_7_S0; CLASS CASE; MODEL DIFFMO_2*DM(0)=DDX_COPD / RISKLIMITS; RUN;

PROC PHREG DATA=SHIM.DIS_1_DM_S_7_S0; CLASS CASE; MODEL DIFFMO_2*DM(0)=DDX_ASTH / RISKLIMITS; RUN;

PROC PHREG DATA=SHIM.DIS_1_DM_S_7_S0; CLASS CASE; MODEL DIFFMO_2*DM(0)=DDX_CAN / RISKLIMITS; RUN;

PROC PHREG DATA=SHIM.DIS_1_DM_S_7_S0; CLASS CASE; MODEL DIFFMO_2*DM(0)=CITY / RISKLIMITS; RUN;

PROC PHREG DATA=SHIM.DIS_1_DM_S_7_S0; CLASS CASE CHA_NEW(REF='1'); MODEL DIFFMO_2*DM(0)=CHA_NEW / RISKLIMITS; RUN;

PROC PHREG DATA=SHIM.DIS_1_DM_S_7_S0; CLASS CASE; MODEL DIFFMO_2*DM(0)=INSUP / RISKLIMITS; RUN;

TITLE '====================== MI ========================';

PROC PHREG DATA=SHIM.DIS_1_MI_S_7_S0; CLASS CASE; MODEL DIFFMO_2*MI(0)=CASE / RISKLIMITS; RUN;

PROC PHREG DATA=SHIM.DIS_1_MI_S_7_S0; CLASS CASE; MODEL DIFFMO_2*MI(0)=TTX_CHOL / RISKLIMITS; RUN;

PROC PHREG DATA=SHIM.DIS_1_MI_S_7_S0; CLASS CASE; MODEL DIFFMO_2*MI(0)=TTX_COA / RISKLIMITS; RUN;

PROC PHREG DATA=SHIM.DIS_1_MI_S_7_S0; CLASS CASE; MODEL DIFFMO_2*MI(0)=TTX_PLT / RISKLIMITS; RUN;

PROC PHREG DATA=SHIM.DIS_1_MI_S_7_S0; CLASS CASE; MODEL DIFFMO_2*MI(0)=TTX_ANT / RISKLIMITS; RUN;

PROC PHREG DATA=SHIM.DIS_1_MI_S_7_S0; CLASS CASE; MODEL DIFFMO_2*MI(0)=DDX_HTN / RISKLIMITS; RUN;

PROC PHREG DATA=SHIM.DIS_1_MI_S_7_S0; CLASS CASE; MODEL DIFFMO_2*MI(0)=DDX_LIV / RISKLIMITS; RUN;

PROC PHREG DATA=SHIM.DIS_1_MI_S_7_S0; CLASS CASE; MODEL DIFFMO_2*MI(0)=DDX_CKD / RISKLIMITS; RUN;

PROC PHREG DATA=SHIM.DIS_1_MI_S_7_S0; CLASS CASE; MODEL DIFFMO_2*MI(0)=DDX_COPD / RISKLIMITS; RUN;

PROC PHREG DATA=SHIM.DIS_1_MI_S_7_S0; CLASS CASE; MODEL DIFFMO_2*MI(0)=DDX_ASTH / RISKLIMITS; RUN;

PROC PHREG DATA=SHIM.DIS_1_MI_S_7_S0; CLASS CASE; MODEL DIFFMO_2*MI(0)=DDX_CAN / RISKLIMITS; RUN;

PROC PHREG DATA=SHIM.DIS_1_MI_S_7_S0; CLASS CASE; MODEL DIFFMO_2*MI(0)=CITY / RISKLIMITS; RUN;

PROC PHREG DATA=SHIM.DIS_1_MI_S_7_S0; CLASS CASE CHA_NEW(REF='1'); MODEL DIFFMO_2*MI(0)=CHA_NEW / RISKLIMITS; RUN;

PROC PHREG DATA=SHIM.DIS_1_MI_S_7_S0; CLASS CASE; MODEL DIFFMO_2*MI(0)=INSUP / RISKLIMITS; RUN;

TITLE '====================== ALZ ========================';

PROC PHREG DATA=SHIM.DIS_2_ALZ_S_7_S0; CLASS CASE; MODEL DIFFMO_2*ALZ(0)=CASE / RISKLIMITS; RUN;

PROC PHREG DATA=SHIM.DIS_2_ALZ_S_7_S0; CLASS CASE; MODEL DIFFMO_2*ALZ(0)=TTX_CHOL / RISKLIMITS; RUN;

PROC PHREG DATA=SHIM.DIS_2_ALZ_S_7_S0; CLASS CASE; MODEL DIFFMO_2*ALZ(0)=TTX_COA / RISKLIMITS; RUN;

PROC PHREG DATA=SHIM.DIS_2_ALZ_S_7_S0; CLASS CASE; MODEL DIFFMO_2*ALZ(0)=TTX_PLT / RISKLIMITS; RUN;

PROC PHREG DATA=SHIM.DIS_2_ALZ_S_7_S0; CLASS CASE; MODEL DIFFMO_2*ALZ(0)=TTX_ANT / RISKLIMITS; RUN;

PROC PHREG DATA=SHIM.DIS_2_ALZ_S_7_S0; CLASS CASE; MODEL DIFFMO_2*ALZ(0)=DDX_HTN / RISKLIMITS; RUN;

PROC PHREG DATA=SHIM.DIS_2_ALZ_S_7_S0; CLASS CASE; MODEL DIFFMO_2*ALZ(0)=DDX_DM / RISKLIMITS; RUN;

PROC PHREG DATA=SHIM.DIS_2_ALZ_S_7_S0; CLASS CASE; MODEL DIFFMO_2*ALZ(0)=CVD / RISKLIMITS; RUN;

PROC PHREG DATA=SHIM.DIS_2_ALZ_S_7_S0; CLASS CASE; MODEL DIFFMO_2*ALZ(0)=DDX_LIV / RISKLIMITS; RUN;

PROC PHREG DATA=SHIM.DIS_2_ALZ_S_7_S0; CLASS CASE; MODEL DIFFMO_2*ALZ(0)=DDX_CKD / RISKLIMITS; RUN;

PROC PHREG DATA=SHIM.DIS_2_ALZ_S_7_S0; CLASS CASE; MODEL DIFFMO_2*ALZ(0)=DDX_COPD / RISKLIMITS; RUN;

PROC PHREG DATA=SHIM.DIS_2_ALZ_S_7_S0; CLASS CASE; MODEL DIFFMO_2*ALZ(0)=DDX_ASTH / RISKLIMITS; RUN;

PROC PHREG DATA=SHIM.DIS_2_ALZ_S_7_S0; CLASS CASE; MODEL DIFFMO_2*ALZ(0)=DDX_CAN / RISKLIMITS; RUN;

PROC PHREG DATA=SHIM.DIS_2_ALZ_S_7_S0; CLASS CASE; MODEL DIFFMO_2*ALZ(0)=CITY / RISKLIMITS; RUN;

PROC PHREG DATA=SHIM.DIS_2_ALZ_S_7_S0; CLASS CASE CHA_NEW(REF='1'); MODEL DIFFMO_2*ALZ(0)=CHA_NEW / RISKLIMITS; RUN;

PROC PHREG DATA=SHIM.DIS_2_ALZ_S_7_S0; CLASS CASE; MODEL DIFFMO_2*ALZ(0)=INSUP / RISKLIMITS; RUN;

TITLE '====================== PKS ========================';

PROC PHREG DATA=SHIM.DIS_2_PKS_S_7_S0; CLASS CASE; MODEL DIFFMO_2*PKS(0)=CASE / RISKLIMITS; RUN;

PROC PHREG DATA=SHIM.DIS_2_PKS_S_7_S0; CLASS CASE; MODEL DIFFMO_2*PKS(0)=TTX_CHOL / RISKLIMITS; RUN;

PROC PHREG DATA=SHIM.DIS_2_PKS_S_7_S0; CLASS CASE; MODEL DIFFMO_2*PKS(0)=TTX_COA / RISKLIMITS; RUN;

PROC PHREG DATA=SHIM.DIS_2_PKS_S_7_S0; CLASS CASE; MODEL DIFFMO_2*PKS(0)=TTX_PLT / RISKLIMITS; RUN;

PROC PHREG DATA=SHIM.DIS_2_PKS_S_7_S0; CLASS CASE; MODEL DIFFMO_2*PKS(0)=TTX_ANT / RISKLIMITS; RUN;

PROC PHREG DATA=SHIM.DIS_2_PKS_S_7_S0; CLASS CASE; MODEL DIFFMO_2*PKS(0)=DDX_HTN / RISKLIMITS; RUN;

PROC PHREG DATA=SHIM.DIS_2_PKS_S_7_S0; CLASS CASE; MODEL DIFFMO_2*PKS(0)=DDX_DM / RISKLIMITS; RUN;

PROC PHREG DATA=SHIM.DIS_2_PKS_S_7_S0; CLASS CASE; MODEL DIFFMO_2*PKS(0)=CVD / RISKLIMITS; RUN;

PROC PHREG DATA=SHIM.DIS_2_PKS_S_7_S0; CLASS CASE; MODEL DIFFMO_2*PKS(0)=DDX_LIV / RISKLIMITS; RUN;

PROC PHREG DATA=SHIM.DIS_2_PKS_S_7_S0; CLASS CASE; MODEL DIFFMO_2*PKS(0)=DDX_CKD / RISKLIMITS; RUN;

PROC PHREG DATA=SHIM.DIS_2_PKS_S_7_S0; CLASS CASE; MODEL DIFFMO_2*PKS(0)=DDX_COPD / RISKLIMITS; RUN;

PROC PHREG DATA=SHIM.DIS_2_PKS_S_7_S0; CLASS CASE; MODEL DIFFMO_2*PKS(0)=DDX_ASTH / RISKLIMITS; RUN;

PROC PHREG DATA=SHIM.DIS_2_PKS_S_7_S0; CLASS CASE; MODEL DIFFMO_2*PKS(0)=DDX_CAN / RISKLIMITS; RUN;

PROC PHREG DATA=SHIM.DIS_2_PKS_S_7_S0; CLASS CASE; MODEL DIFFMO_2*PKS(0)=CITY / RISKLIMITS; RUN;

PROC PHREG DATA=SHIM.DIS_2_PKS_S_7_S0; CLASS CASE CHA_NEW(REF='1'); MODEL DIFFMO_2*PKS(0)=CHA_NEW / RISKLIMITS; RUN;

PROC PHREG DATA=SHIM.DIS_2_PKS_S_7_S0; CLASS CASE; MODEL DIFFMO_2*PKS(0)=INSUP / RISKLIMITS; RUN;

TITLE '*****************************SAGE = 1 ***************************';

TITLE '====================== CVA ========================';

PROC PHREG DATA=SHIM.DIS_1_CVA_S_7_S1; CLASS CASE; MODEL DIFFMO_2*CVA(0)=CASE / RISKLIMITS; RUN;

PROC PHREG DATA=SHIM.DIS_1_CVA_S_7_S1; CLASS CASE; MODEL DIFFMO_2*CVA(0)=TTX_CHOL / RISKLIMITS; RUN;

PROC PHREG DATA=SHIM.DIS_1_CVA_S_7_S1; CLASS CASE; MODEL DIFFMO_2*CVA(0)=TTX_COA / RISKLIMITS; RUN;

PROC PHREG DATA=SHIM.DIS_1_CVA_S_7_S1; CLASS CASE; MODEL DIFFMO_2*CVA(0)=TTX_PLT / RISKLIMITS; RUN;

PROC PHREG DATA=SHIM.DIS_1_CVA_S_7_S1; CLASS CASE; MODEL DIFFMO_2*CVA(0)=TTX_ANT / RISKLIMITS; RUN;

PROC PHREG DATA=SHIM.DIS_1_CVA_S_7_S1; CLASS CASE; MODEL DIFFMO_2*CVA(0)=DDX_HTN / RISKLIMITS; RUN;

PROC PHREG DATA=SHIM.DIS_1_CVA_S_7_S1; CLASS CASE; MODEL DIFFMO_2*CVA(0)=DDX_LIV / RISKLIMITS; RUN;

PROC PHREG DATA=SHIM.DIS_1_CVA_S_7_S1; CLASS CASE; MODEL DIFFMO_2*CVA(0)=DDX_CKD / RISKLIMITS; RUN;

PROC PHREG DATA=SHIM.DIS_1_CVA_S_7_S1; CLASS CASE; MODEL DIFFMO_2*CVA(0)=DDX_COPD / RISKLIMITS; RUN;

PROC PHREG DATA=SHIM.DIS_1_CVA_S_7_S1; CLASS CASE; MODEL DIFFMO_2*CVA(0)=DDX_ASTH / RISKLIMITS; RUN;

PROC PHREG DATA=SHIM.DIS_1_CVA_S_7_S1; CLASS CASE; MODEL DIFFMO_2*CVA(0)=DDX_CAN / RISKLIMITS; RUN;

PROC PHREG DATA=SHIM.DIS_1_CVA_S_7_S1; CLASS CASE; MODEL DIFFMO_2*CVA(0)=CITY / RISKLIMITS; RUN;

PROC PHREG DATA=SHIM.DIS_1_CVA_S_7_S1; CLASS CASE CHA_NEW(REF='1') ; MODEL DIFFMO_2*CVA(0)=CHA_NEW / RISKLIMITS; RUN;

PROC PHREG DATA=SHIM.DIS_1_CVA_S_7_S1; CLASS CASE; MODEL DIFFMO_2*CVA(0)=INSUP / RISKLIMITS; RUN;

TITLE '====================== IHD ========================';

PROC PHREG DATA=SHIM.DIS_1_IHD_S_7_S1; CLASS CASE; MODEL DIFFMO_2*IHD(0)=CASE / RISKLIMITS; RUN;

PROC PHREG DATA=SHIM.DIS_1_IHD_S_7_S1; CLASS CASE; MODEL DIFFMO_2*IHD(0)=TTX_CHOL / RISKLIMITS; RUN;

PROC PHREG DATA=SHIM.DIS_1_IHD_S_7_S1; CLASS CASE; MODEL DIFFMO_2*IHD(0)=TTX_COA / RISKLIMITS; RUN;

PROC PHREG DATA=SHIM.DIS_1_IHD_S_7_S1; CLASS CASE; MODEL DIFFMO_2*IHD(0)=TTX_PLT / RISKLIMITS; RUN;

PROC PHREG DATA=SHIM.DIS_1_IHD_S_7_S1; CLASS CASE; MODEL DIFFMO_2*IHD(0)=TTX_ANT / RISKLIMITS; RUN;

PROC PHREG DATA=SHIM.DIS_1_IHD_S_7_S1; CLASS CASE; MODEL DIFFMO_2*IHD(0)=DDX_HTN / RISKLIMITS; RUN;

PROC PHREG DATA=SHIM.DIS_1_IHD_S_7_S1; CLASS CASE; MODEL DIFFMO_2*IHD(0)=DDX_LIV / RISKLIMITS; RUN;

PROC PHREG DATA=SHIM.DIS_1_IHD_S_7_S1; CLASS CASE; MODEL DIFFMO_2*IHD(0)=DDX_CKD / RISKLIMITS; RUN;

PROC PHREG DATA=SHIM.DIS_1_IHD_S_7_S1; CLASS CASE; MODEL DIFFMO_2*IHD(0)=DDX_COPD / RISKLIMITS; RUN;

PROC PHREG DATA=SHIM.DIS_1_IHD_S_7_S1; CLASS CASE; MODEL DIFFMO_2*IHD(0)=DDX_ASTH / RISKLIMITS; RUN;

PROC PHREG DATA=SHIM.DIS_1_IHD_S_7_S1; CLASS CASE; MODEL DIFFMO_2*IHD(0)=DDX_CAN / RISKLIMITS; RUN;

PROC PHREG DATA=SHIM.DIS_1_IHD_S_7_S1; CLASS CASE; MODEL DIFFMO_2*IHD(0)=CITY / RISKLIMITS; RUN;

PROC PHREG DATA=SHIM.DIS_1_IHD_S_7_S1; CLASS CASE CHA_NEW(REF='1'); MODEL DIFFMO_2*IHD(0)=CHA_NEW / RISKLIMITS; RUN;

PROC PHREG DATA=SHIM.DIS_1_IHD_S_7_S1; CLASS CASE; MODEL DIFFMO_2*IHD(0)=INSUP / RISKLIMITS; RUN;

TITLE '====================== DM ========================';

PROC PHREG DATA=SHIM.DIS_1_DM_S_7_S1; CLASS CASE; MODEL DIFFMO_2*DM(0)=CASE / RISKLIMITS; RUN;

PROC PHREG DATA=SHIM.DIS_1_DM_S_7_S1; CLASS CASE; MODEL DIFFMO_2*DM(0)=TTX_CHOL / RISKLIMITS; RUN;

PROC PHREG DATA=SHIM.DIS_1_DM_S_7_S1; CLASS CASE; MODEL DIFFMO_2*DM(0)=TTX_COA / RISKLIMITS; RUN;

PROC PHREG DATA=SHIM.DIS_1_DM_S_7_S1; CLASS CASE; MODEL DIFFMO_2*DM(0)=TTX_PLT / RISKLIMITS; RUN;

PROC PHREG DATA=SHIM.DIS_1_DM_S_7_S1; CLASS CASE; MODEL DIFFMO_2*DM(0)=TTX_ANT / RISKLIMITS; RUN;

PROC PHREG DATA=SHIM.DIS_1_DM_S_7_S1; CLASS CASE; MODEL DIFFMO_2*DM(0)=DDX_HTN / RISKLIMITS; RUN;

PROC PHREG DATA=SHIM.DIS_1_DM_S_7_S1; CLASS CASE; MODEL DIFFMO_2*DM(0)=DDX_LIV / RISKLIMITS; RUN;

PROC PHREG DATA=SHIM.DIS_1_DM_S_7_S1; CLASS CASE; MODEL DIFFMO_2*DM(0)=DDX_CKD / RISKLIMITS; RUN;

PROC PHREG DATA=SHIM.DIS_1_DM_S_7_S1; CLASS CASE; MODEL DIFFMO_2*DM(0)=DDX_COPD / RISKLIMITS; RUN;

PROC PHREG DATA=SHIM.DIS_1_DM_S_7_S1; CLASS CASE; MODEL DIFFMO_2*DM(0)=DDX_ASTH / RISKLIMITS; RUN;

PROC PHREG DATA=SHIM.DIS_1_DM_S_7_S1; CLASS CASE; MODEL DIFFMO_2*DM(0)=DDX_CAN / RISKLIMITS; RUN;

PROC PHREG DATA=SHIM.DIS_1_DM_S_7_S1; CLASS CASE; MODEL DIFFMO_2*DM(0)=CITY / RISKLIMITS; RUN;

PROC PHREG DATA=SHIM.DIS_1_DM_S_7_S1; CLASS CASE CHA_NEW(REF='1'); MODEL DIFFMO_2*DM(0)=CHA_NEW / RISKLIMITS; RUN;

PROC PHREG DATA=SHIM.DIS_1_DM_S_7_S1; CLASS CASE; MODEL DIFFMO_2*DM(0)=INSUP / RISKLIMITS; RUN;

TITLE '====================== MI ========================';

PROC PHREG DATA=SHIM.DIS_1_MI_S_7_S1; CLASS CASE; MODEL DIFFMO_2*MI(0)=CASE / RISKLIMITS; RUN;

PROC PHREG DATA=SHIM.DIS_1_MI_S_7_S1; CLASS CASE; MODEL DIFFMO_2*MI(0)=TTX_CHOL / RISKLIMITS; RUN;

PROC PHREG DATA=SHIM.DIS_1_MI_S_7_S1; CLASS CASE; MODEL DIFFMO_2*MI(0)=TTX_COA / RISKLIMITS; RUN;

PROC PHREG DATA=SHIM.DIS_1_MI_S_7_S1; CLASS CASE; MODEL DIFFMO_2*MI(0)=TTX_PLT / RISKLIMITS; RUN;

PROC PHREG DATA=SHIM.DIS_1_MI_S_7_S1; CLASS CASE; MODEL DIFFMO_2*MI(0)=TTX_ANT / RISKLIMITS; RUN;

PROC PHREG DATA=SHIM.DIS_1_MI_S_7_S1; CLASS CASE; MODEL DIFFMO_2*MI(0)=DDX_HTN / RISKLIMITS; RUN;

PROC PHREG DATA=SHIM.DIS_1_MI_S_7_S1; CLASS CASE; MODEL DIFFMO_2*MI(0)=DDX_LIV / RISKLIMITS; RUN;

PROC PHREG DATA=SHIM.DIS_1_MI_S_7_S1; CLASS CASE; MODEL DIFFMO_2*MI(0)=DDX_CKD / RISKLIMITS; RUN;

PROC PHREG DATA=SHIM.DIS_1_MI_S_7_S1; CLASS CASE; MODEL DIFFMO_2*MI(0)=DDX_COPD / RISKLIMITS; RUN;

PROC PHREG DATA=SHIM.DIS_1_MI_S_7_S1; CLASS CASE; MODEL DIFFMO_2*MI(0)=DDX_ASTH / RISKLIMITS; RUN;

PROC PHREG DATA=SHIM.DIS_1_MI_S_7_S1; CLASS CASE; MODEL DIFFMO_2*MI(0)=DDX_CAN / RISKLIMITS; RUN;

PROC PHREG DATA=SHIM.DIS_1_MI_S_7_S1; CLASS CASE; MODEL DIFFMO_2*MI(0)=CITY / RISKLIMITS; RUN;

PROC PHREG DATA=SHIM.DIS_1_MI_S_7_S1; CLASS CASE CHA_NEW(REF='1'); MODEL DIFFMO_2*MI(0)=CHA_NEW / RISKLIMITS; RUN;

PROC PHREG DATA=SHIM.DIS_1_MI_S_7_S1; CLASS CASE; MODEL DIFFMO_2*MI(0)=INSUP / RISKLIMITS; RUN;

TITLE '====================== ALZ ========================';

PROC PHREG DATA=SHIM.DIS_2_ALZ_S_7_S1; CLASS CASE; MODEL DIFFMO_2*ALZ(0)=CASE / RISKLIMITS; RUN;

PROC PHREG DATA=SHIM.DIS_2_ALZ_S_7_S1; CLASS CASE; MODEL DIFFMO_2*ALZ(0)=TTX_CHOL / RISKLIMITS; RUN;

PROC PHREG DATA=SHIM.DIS_2_ALZ_S_7_S1; CLASS CASE; MODEL DIFFMO_2*ALZ(0)=TTX_COA / RISKLIMITS; RUN;

PROC PHREG DATA=SHIM.DIS_2_ALZ_S_7_S1; CLASS CASE; MODEL DIFFMO_2*ALZ(0)=TTX_PLT / RISKLIMITS; RUN;

PROC PHREG DATA=SHIM.DIS_2_ALZ_S_7_S1; CLASS CASE; MODEL DIFFMO_2*ALZ(0)=TTX_ANT / RISKLIMITS; RUN;

PROC PHREG DATA=SHIM.DIS_2_ALZ_S_7_S1; CLASS CASE; MODEL DIFFMO_2*ALZ(0)=DDX_HTN / RISKLIMITS; RUN;

PROC PHREG DATA=SHIM.DIS_2_ALZ_S_7_S1; CLASS CASE; MODEL DIFFMO_2*ALZ(0)=DDX_DM / RISKLIMITS; RUN;

PROC PHREG DATA=SHIM.DIS_2_ALZ_S_7_S1; CLASS CASE; MODEL DIFFMO_2*ALZ(0)=CVD / RISKLIMITS; RUN;

PROC PHREG DATA=SHIM.DIS_2_ALZ_S_7_S1; CLASS CASE; MODEL DIFFMO_2*ALZ(0)=DDX_LIV / RISKLIMITS; RUN;

PROC PHREG DATA=SHIM.DIS_2_ALZ_S_7_S1; CLASS CASE; MODEL DIFFMO_2*ALZ(0)=DDX_CKD / RISKLIMITS; RUN;

PROC PHREG DATA=SHIM.DIS_2_ALZ_S_7_S1; CLASS CASE; MODEL DIFFMO_2*ALZ(0)=DDX_COPD / RISKLIMITS; RUN;

PROC PHREG DATA=SHIM.DIS_2_ALZ_S_7_S1; CLASS CASE; MODEL DIFFMO_2*ALZ(0)=DDX_ASTH / RISKLIMITS; RUN;

PROC PHREG DATA=SHIM.DIS_2_ALZ_S_7_S1; CLASS CASE; MODEL DIFFMO_2*ALZ(0)=DDX_CAN / RISKLIMITS; RUN;

PROC PHREG DATA=SHIM.DIS_2_ALZ_S_7_S1; CLASS CASE; MODEL DIFFMO_2*ALZ(0)=CITY / RISKLIMITS; RUN;

PROC PHREG DATA=SHIM.DIS_2_ALZ_S_7_S1; CLASS CASE CHA_NEW(REF='1'); MODEL DIFFMO_2*ALZ(0)=CHA_NEW / RISKLIMITS; RUN;

PROC PHREG DATA=SHIM.DIS_2_ALZ_S_7_S1; CLASS CASE; MODEL DIFFMO_2*ALZ(0)=INSUP / RISKLIMITS; RUN;

TITLE '====================== PKS ========================';

PROC PHREG DATA=SHIM.DIS_2_PKS_S_7_S1; CLASS CASE; MODEL DIFFMO_2*PKS(0)=CASE / RISKLIMITS; RUN;

PROC PHREG DATA=SHIM.DIS_2_PKS_S_7_S1; CLASS CASE; MODEL DIFFMO_2*PKS(0)=TTX_CHOL / RISKLIMITS; RUN;

PROC PHREG DATA=SHIM.DIS_2_PKS_S_7_S1; CLASS CASE; MODEL DIFFMO_2*PKS(0)=TTX_COA / RISKLIMITS; RUN;

PROC PHREG DATA=SHIM.DIS_2_PKS_S_7_S1; CLASS CASE; MODEL DIFFMO_2*PKS(0)=TTX_PLT / RISKLIMITS; RUN;

PROC PHREG DATA=SHIM.DIS_2_PKS_S_7_S1; CLASS CASE; MODEL DIFFMO_2*PKS(0)=TTX_ANT / RISKLIMITS; RUN;

PROC PHREG DATA=SHIM.DIS_2_PKS_S_7_S1; CLASS CASE; MODEL DIFFMO_2*PKS(0)=DDX_HTN / RISKLIMITS; RUN;

PROC PHREG DATA=SHIM.DIS_2_PKS_S_7_S1; CLASS CASE; MODEL DIFFMO_2*PKS(0)=DDX_DM / RISKLIMITS; RUN;

PROC PHREG DATA=SHIM.DIS_2_PKS_S_7_S1; CLASS CASE; MODEL DIFFMO_2*PKS(0)=CVD / RISKLIMITS; RUN;

PROC PHREG DATA=SHIM.DIS_2_PKS_S_7_S1; CLASS CASE; MODEL DIFFMO_2*PKS(0)=DDX_LIV / RISKLIMITS; RUN;

PROC PHREG DATA=SHIM.DIS_2_PKS_S_7_S1; CLASS CASE; MODEL DIFFMO_2*PKS(0)=DDX_CKD / RISKLIMITS; RUN;

PROC PHREG DATA=SHIM.DIS_2_PKS_S_7_S1; CLASS CASE; MODEL DIFFMO_2*PKS(0)=DDX_COPD / RISKLIMITS; RUN;

PROC PHREG DATA=SHIM.DIS_2_PKS_S_7_S1; CLASS CASE; MODEL DIFFMO_2*PKS(0)=DDX_ASTH / RISKLIMITS; RUN;

PROC PHREG DATA=SHIM.DIS_2_PKS_S_7_S1; CLASS CASE; MODEL DIFFMO_2*PKS(0)=DDX_CAN / RISKLIMITS; RUN;

PROC PHREG DATA=SHIM.DIS_2_PKS_S_7_S1; CLASS CASE; MODEL DIFFMO_2*PKS(0)=CITY / RISKLIMITS; RUN;

PROC PHREG DATA=SHIM.DIS_2_PKS_S_7_S1; CLASS CASE CHA_NEW(REF='1'); MODEL DIFFMO_2*PKS(0)=CHA_NEW / RISKLIMITS; RUN;

PROC PHREG DATA=SHIM.DIS_2_PKS_S_7_S1; CLASS CASE; MODEL DIFFMO_2*PKS(0)=INSUP / RISKLIMITS; RUN;

TITLE '*****************************SAGE = 2***************************';

TITLE '====================== CVA ========================';

PROC PHREG DATA=SHIM.DIS_1_CVA_S_7_S2; CLASS CASE; MODEL DIFFMO_2*CVA(0)=CASE / RISKLIMITS; RUN;

PROC PHREG DATA=SHIM.DIS_1_CVA_S_7_S2; CLASS CASE; MODEL DIFFMO_2*CVA(0)=TTX_CHOL / RISKLIMITS; RUN;

PROC PHREG DATA=SHIM.DIS_1_CVA_S_7_S2; CLASS CASE; MODEL DIFFMO_2*CVA(0)=TTX_COA / RISKLIMITS; RUN;

PROC PHREG DATA=SHIM.DIS_1_CVA_S_7_S2; CLASS CASE; MODEL DIFFMO_2*CVA(0)=TTX_PLT / RISKLIMITS; RUN;

PROC PHREG DATA=SHIM.DIS_1_CVA_S_7_S2; CLASS CASE; MODEL DIFFMO_2*CVA(0)=TTX_ANT / RISKLIMITS; RUN;

PROC PHREG DATA=SHIM.DIS_1_CVA_S_7_S2; CLASS CASE; MODEL DIFFMO_2*CVA(0)=DDX_HTN / RISKLIMITS; RUN;

PROC PHREG DATA=SHIM.DIS_1_CVA_S_7_S2; CLASS CASE; MODEL DIFFMO_2*CVA(0)=DDX_LIV / RISKLIMITS; RUN;

PROC PHREG DATA=SHIM.DIS_1_CVA_S_7_S2; CLASS CASE; MODEL DIFFMO_2*CVA(0)=DDX_CKD / RISKLIMITS; RUN;

PROC PHREG DATA=SHIM.DIS_1_CVA_S_7_S2; CLASS CASE; MODEL DIFFMO_2*CVA(0)=DDX_COPD / RISKLIMITS; RUN;

PROC PHREG DATA=SHIM.DIS_1_CVA_S_7_S2; CLASS CASE; MODEL DIFFMO_2*CVA(0)=DDX_ASTH / RISKLIMITS; RUN;

PROC PHREG DATA=SHIM.DIS_1_CVA_S_7_S2; CLASS CASE; MODEL DIFFMO_2*CVA(0)=DDX_CAN / RISKLIMITS; RUN;

PROC PHREG DATA=SHIM.DIS_1_CVA_S_7_S2; CLASS CASE; MODEL DIFFMO_2*CVA(0)=CITY / RISKLIMITS; RUN;

PROC PHREG DATA=SHIM.DIS_1_CVA_S_7_S2; CLASS CASE CHA_NEW(REF='1') ; MODEL DIFFMO_2*CVA(0)=CHA_NEW / RISKLIMITS; RUN;

PROC PHREG DATA=SHIM.DIS_1_CVA_S_7_S2; CLASS CASE; MODEL DIFFMO_2*CVA(0)=INSUP / RISKLIMITS; RUN;

TITLE '====================== IHD ========================';

PROC PHREG DATA=SHIM.DIS_1_IHD_S_7_S2; CLASS CASE; MODEL DIFFMO_2*IHD(0)=CASE / RISKLIMITS; RUN;

PROC PHREG DATA=SHIM.DIS_1_IHD_S_7_S2; CLASS CASE; MODEL DIFFMO_2*IHD(0)=TTX_CHOL / RISKLIMITS; RUN;

PROC PHREG DATA=SHIM.DIS_1_IHD_S_7_S2; CLASS CASE; MODEL DIFFMO_2*IHD(0)=TTX_COA / RISKLIMITS; RUN;

PROC PHREG DATA=SHIM.DIS_1_IHD_S_7_S2; CLASS CASE; MODEL DIFFMO_2*IHD(0)=TTX_PLT / RISKLIMITS; RUN;

PROC PHREG DATA=SHIM.DIS_1_IHD_S_7_S2; CLASS CASE; MODEL DIFFMO_2*IHD(0)=TTX_ANT / RISKLIMITS; RUN;

PROC PHREG DATA=SHIM.DIS_1_IHD_S_7_S2; CLASS CASE; MODEL DIFFMO_2*IHD(0)=DDX_HTN / RISKLIMITS; RUN;

PROC PHREG DATA=SHIM.DIS_1_IHD_S_7_S2; CLASS CASE; MODEL DIFFMO_2*IHD(0)=DDX_LIV / RISKLIMITS; RUN;

PROC PHREG DATA=SHIM.DIS_1_IHD_S_7_S2; CLASS CASE; MODEL DIFFMO_2*IHD(0)=DDX_CKD / RISKLIMITS; RUN;

PROC PHREG DATA=SHIM.DIS_1_IHD_S_7_S2; CLASS CASE; MODEL DIFFMO_2*IHD(0)=DDX_COPD / RISKLIMITS; RUN;

PROC PHREG DATA=SHIM.DIS_1_IHD_S_7_S2; CLASS CASE; MODEL DIFFMO_2*IHD(0)=DDX_ASTH / RISKLIMITS; RUN;

PROC PHREG DATA=SHIM.DIS_1_IHD_S_7_S2; CLASS CASE; MODEL DIFFMO_2*IHD(0)=DDX_CAN / RISKLIMITS; RUN;

PROC PHREG DATA=SHIM.DIS_1_IHD_S_7_S2; CLASS CASE; MODEL DIFFMO_2*IHD(0)=CITY / RISKLIMITS; RUN;

PROC PHREG DATA=SHIM.DIS_1_IHD_S_7_S2; CLASS CASE CHA_NEW(REF='1'); MODEL DIFFMO_2*IHD(0)=CHA_NEW / RISKLIMITS; RUN;

PROC PHREG DATA=SHIM.DIS_1_IHD_S_7_S2; CLASS CASE; MODEL DIFFMO_2*IHD(0)=INSUP / RISKLIMITS; RUN;

TITLE '====================== DM ========================';

PROC PHREG DATA=SHIM.DIS_1_DM_S_7_S2; CLASS CASE; MODEL DIFFMO_2*DM(0)=CASE / RISKLIMITS; RUN;

PROC PHREG DATA=SHIM.DIS_1_DM_S_7_S2; CLASS CASE; MODEL DIFFMO_2*DM(0)=TTX_CHOL / RISKLIMITS; RUN;

PROC PHREG DATA=SHIM.DIS_1_DM_S_7_S2; CLASS CASE; MODEL DIFFMO_2*DM(0)=TTX_COA / RISKLIMITS; RUN;

PROC PHREG DATA=SHIM.DIS_1_DM_S_7_S2; CLASS CASE; MODEL DIFFMO_2*DM(0)=TTX_PLT / RISKLIMITS; RUN;

PROC PHREG DATA=SHIM.DIS_1_DM_S_7_S2; CLASS CASE; MODEL DIFFMO_2*DM(0)=TTX_ANT / RISKLIMITS; RUN;

PROC PHREG DATA=SHIM.DIS_1_DM_S_7_S2; CLASS CASE; MODEL DIFFMO_2*DM(0)=DDX_HTN / RISKLIMITS; RUN;

PROC PHREG DATA=SHIM.DIS_1_DM_S_7_S2; CLASS CASE; MODEL DIFFMO_2*DM(0)=DDX_LIV / RISKLIMITS; RUN;

PROC PHREG DATA=SHIM.DIS_1_DM_S_7_S2; CLASS CASE; MODEL DIFFMO_2*DM(0)=DDX_CKD / RISKLIMITS; RUN;

PROC PHREG DATA=SHIM.DIS_1_DM_S_7_S2; CLASS CASE; MODEL DIFFMO_2*DM(0)=DDX_COPD / RISKLIMITS; RUN;

PROC PHREG DATA=SHIM.DIS_1_DM_S_7_S2; CLASS CASE; MODEL DIFFMO_2*DM(0)=DDX_ASTH / RISKLIMITS; RUN;

PROC PHREG DATA=SHIM.DIS_1_DM_S_7_S2; CLASS CASE; MODEL DIFFMO_2*DM(0)=DDX_CAN / RISKLIMITS; RUN;

PROC PHREG DATA=SHIM.DIS_1_DM_S_7_S2; CLASS CASE; MODEL DIFFMO_2*DM(0)=CITY / RISKLIMITS; RUN;

PROC PHREG DATA=SHIM.DIS_1_DM_S_7_S2; CLASS CASE CHA_NEW(REF='1'); MODEL DIFFMO_2*DM(0)=CHA_NEW / RISKLIMITS; RUN;

PROC PHREG DATA=SHIM.DIS_1_DM_S_7_S2; CLASS CASE; MODEL DIFFMO_2*DM(0)=INSUP / RISKLIMITS; RUN;

TITLE '====================== MI ========================';

PROC PHREG DATA=SHIM.DIS_1_MI_S_7_S2; CLASS CASE; MODEL DIFFMO_2*MI(0)=CASE / RISKLIMITS; RUN;

PROC PHREG DATA=SHIM.DIS_1_MI_S_7_S2; CLASS CASE; MODEL DIFFMO_2*MI(0)=TTX_CHOL / RISKLIMITS; RUN;

PROC PHREG DATA=SHIM.DIS_1_MI_S_7_S2; CLASS CASE; MODEL DIFFMO_2*MI(0)=TTX_COA / RISKLIMITS; RUN;

PROC PHREG DATA=SHIM.DIS_1_MI_S_7_S2; CLASS CASE; MODEL DIFFMO_2*MI(0)=TTX_PLT / RISKLIMITS; RUN;

PROC PHREG DATA=SHIM.DIS_1_MI_S_7_S2; CLASS CASE; MODEL DIFFMO_2*MI(0)=TTX_ANT / RISKLIMITS; RUN;

PROC PHREG DATA=SHIM.DIS_1_MI_S_7_S2; CLASS CASE; MODEL DIFFMO_2*MI(0)=DDX_HTN / RISKLIMITS; RUN;

PROC PHREG DATA=SHIM.DIS_1_MI_S_7_S2; CLASS CASE; MODEL DIFFMO_2*MI(0)=DDX_LIV / RISKLIMITS; RUN;

PROC PHREG DATA=SHIM.DIS_1_MI_S_7_S2; CLASS CASE; MODEL DIFFMO_2*MI(0)=DDX_CKD / RISKLIMITS; RUN;

PROC PHREG DATA=SHIM.DIS_1_MI_S_7_S2; CLASS CASE; MODEL DIFFMO_2*MI(0)=DDX_COPD / RISKLIMITS; RUN;

PROC PHREG DATA=SHIM.DIS_1_MI_S_7_S2; CLASS CASE; MODEL DIFFMO_2*MI(0)=DDX_ASTH / RISKLIMITS; RUN;

PROC PHREG DATA=SHIM.DIS_1_MI_S_7_S2; CLASS CASE; MODEL DIFFMO_2*MI(0)=DDX_CAN / RISKLIMITS; RUN;

PROC PHREG DATA=SHIM.DIS_1_MI_S_7_S2; CLASS CASE; MODEL DIFFMO_2*MI(0)=CITY / RISKLIMITS; RUN;

PROC PHREG DATA=SHIM.DIS_1_MI_S_7_S2; CLASS CASE CHA_NEW(REF='1'); MODEL DIFFMO_2*MI(0)=CHA_NEW / RISKLIMITS; RUN;

PROC PHREG DATA=SHIM.DIS_1_MI_S_7_S2; CLASS CASE; MODEL DIFFMO_2*MI(0)=INSUP / RISKLIMITS; RUN;

TITLE '====================== ALZ ========================';

PROC PHREG DATA=SHIM.DIS_2_ALZ_S_7_S2; CLASS CASE; MODEL DIFFMO_2*ALZ(0)=CASE / RISKLIMITS; RUN;

PROC PHREG DATA=SHIM.DIS_2_ALZ_S_7_S2; CLASS CASE; MODEL DIFFMO_2*ALZ(0)=TTX_CHOL / RISKLIMITS; RUN;

PROC PHREG DATA=SHIM.DIS_2_ALZ_S_7_S2; CLASS CASE; MODEL DIFFMO_2*ALZ(0)=TTX_COA / RISKLIMITS; RUN;

PROC PHREG DATA=SHIM.DIS_2_ALZ_S_7_S2; CLASS CASE; MODEL DIFFMO_2*ALZ(0)=TTX_PLT / RISKLIMITS; RUN;

PROC PHREG DATA=SHIM.DIS_2_ALZ_S_7_S2; CLASS CASE; MODEL DIFFMO_2*ALZ(0)=TTX_ANT / RISKLIMITS; RUN;

PROC PHREG DATA=SHIM.DIS_2_ALZ_S_7_S2; CLASS CASE; MODEL DIFFMO_2*ALZ(0)=DDX_HTN / RISKLIMITS; RUN;

PROC PHREG DATA=SHIM.DIS_2_ALZ_S_7_S2; CLASS CASE; MODEL DIFFMO_2*ALZ(0)=DDX_DM / RISKLIMITS; RUN;

PROC PHREG DATA=SHIM.DIS_2_ALZ_S_7_S2; CLASS CASE; MODEL DIFFMO_2*ALZ(0)=CVD / RISKLIMITS; RUN;

PROC PHREG DATA=SHIM.DIS_2_ALZ_S_7_S2; CLASS CASE; MODEL DIFFMO_2*ALZ(0)=DDX_LIV / RISKLIMITS; RUN;

PROC PHREG DATA=SHIM.DIS_2_ALZ_S_7_S2; CLASS CASE; MODEL DIFFMO_2*ALZ(0)=DDX_CKD / RISKLIMITS; RUN;

PROC PHREG DATA=SHIM.DIS_2_ALZ_S_7_S2; CLASS CASE; MODEL DIFFMO_2*ALZ(0)=DDX_COPD / RISKLIMITS; RUN;

PROC PHREG DATA=SHIM.DIS_2_ALZ_S_7_S2; CLASS CASE; MODEL DIFFMO_2*ALZ(0)=DDX_ASTH / RISKLIMITS; RUN;

PROC PHREG DATA=SHIM.DIS_2_ALZ_S_7_S2; CLASS CASE; MODEL DIFFMO_2*ALZ(0)=DDX_CAN / RISKLIMITS; RUN;

PROC PHREG DATA=SHIM.DIS_2_ALZ_S_7_S2; CLASS CASE; MODEL DIFFMO_2*ALZ(0)=CITY / RISKLIMITS; RUN;

PROC PHREG DATA=SHIM.DIS_2_ALZ_S_7_S2; CLASS CASE CHA_NEW(REF='1'); MODEL DIFFMO_2*ALZ(0)=CHA_NEW / RISKLIMITS; RUN;

PROC PHREG DATA=SHIM.DIS_2_ALZ_S_7_S2; CLASS CASE; MODEL DIFFMO_2*ALZ(0)=INSUP / RISKLIMITS; RUN;

TITLE '====================== PKS ========================';

PROC PHREG DATA=SHIM.DIS_2_PKS_S_7_S2; CLASS CASE; MODEL DIFFMO_2*PKS(0)=CASE / RISKLIMITS; RUN;

PROC PHREG DATA=SHIM.DIS_2_PKS_S_7_S2; CLASS CASE; MODEL DIFFMO_2*PKS(0)=TTX_CHOL / RISKLIMITS; RUN;

PROC PHREG DATA=SHIM.DIS_2_PKS_S_7_S2; CLASS CASE; MODEL DIFFMO_2*PKS(0)=TTX_COA / RISKLIMITS; RUN;

PROC PHREG DATA=SHIM.DIS_2_PKS_S_7_S2; CLASS CASE; MODEL DIFFMO_2*PKS(0)=TTX_PLT / RISKLIMITS; RUN;

PROC PHREG DATA=SHIM.DIS_2_PKS_S_7_S2; CLASS CASE; MODEL DIFFMO_2*PKS(0)=TTX_ANT / RISKLIMITS; RUN;

PROC PHREG DATA=SHIM.DIS_2_PKS_S_7_S2; CLASS CASE; MODEL DIFFMO_2*PKS(0)=DDX_HTN / RISKLIMITS; RUN;

PROC PHREG DATA=SHIM.DIS_2_PKS_S_7_S2; CLASS CASE; MODEL DIFFMO_2*PKS(0)=DDX_DM / RISKLIMITS; RUN;

PROC PHREG DATA=SHIM.DIS_2_PKS_S_7_S2; CLASS CASE; MODEL DIFFMO_2*PKS(0)=CVD / RISKLIMITS; RUN;

PROC PHREG DATA=SHIM.DIS_2_PKS_S_7_S2; CLASS CASE; MODEL DIFFMO_2*PKS(0)=DDX_LIV / RISKLIMITS; RUN;

PROC PHREG DATA=SHIM.DIS_2_PKS_S_7_S2; CLASS CASE; MODEL DIFFMO_2*PKS(0)=DDX_CKD / RISKLIMITS; RUN;

PROC PHREG DATA=SHIM.DIS_2_PKS_S_7_S2; CLASS CASE; MODEL DIFFMO_2*PKS(0)=DDX_COPD / RISKLIMITS; RUN;

PROC PHREG DATA=SHIM.DIS_2_PKS_S_7_S2; CLASS CASE; MODEL DIFFMO_2*PKS(0)=DDX_ASTH / RISKLIMITS; RUN;

PROC PHREG DATA=SHIM.DIS_2_PKS_S_7_S2; CLASS CASE; MODEL DIFFMO_2*PKS(0)=DDX_CAN / RISKLIMITS; RUN;

PROC PHREG DATA=SHIM.DIS_2_PKS_S_7_S2; CLASS CASE; MODEL DIFFMO_2*PKS(0)=CITY / RISKLIMITS; RUN;

PROC PHREG DATA=SHIM.DIS_2_PKS_S_7_S2; CLASS CASE CHA_NEW(REF='1'); MODEL DIFFMO_2*PKS(0)=CHA_NEW / RISKLIMITS; RUN;

PROC PHREG DATA=SHIM.DIS_2_PKS_S_7_S2; CLASS CASE; MODEL DIFFMO_2*PKS(0)=INSUP / RISKLIMITS; RUN;

TITLE '*****************************SAGE = 3***************************';

TITLE '====================== CVA ========================';

PROC PHREG DATA=SHIM.DIS_1_CVA_S_7_S3; CLASS CASE; MODEL DIFFMO_2*CVA(0)=CASE / RISKLIMITS; RUN;

PROC PHREG DATA=SHIM.DIS_1_CVA_S_7_S3; CLASS CASE; MODEL DIFFMO_2*CVA(0)=TTX_CHOL / RISKLIMITS; RUN;

PROC PHREG DATA=SHIM.DIS_1_CVA_S_7_S3; CLASS CASE; MODEL DIFFMO_2*CVA(0)=TTX_COA / RISKLIMITS; RUN;

PROC PHREG DATA=SHIM.DIS_1_CVA_S_7_S3; CLASS CASE; MODEL DIFFMO_2*CVA(0)=TTX_PLT / RISKLIMITS; RUN;

PROC PHREG DATA=SHIM.DIS_1_CVA_S_7_S3; CLASS CASE; MODEL DIFFMO_2*CVA(0)=TTX_ANT / RISKLIMITS; RUN;

PROC PHREG DATA=SHIM.DIS_1_CVA_S_7_S3; CLASS CASE; MODEL DIFFMO_2*CVA(0)=DDX_HTN / RISKLIMITS; RUN;

PROC PHREG DATA=SHIM.DIS_1_CVA_S_7_S3; CLASS CASE; MODEL DIFFMO_2*CVA(0)=DDX_LIV / RISKLIMITS; RUN;

PROC PHREG DATA=SHIM.DIS_1_CVA_S_7_S3; CLASS CASE; MODEL DIFFMO_2*CVA(0)=DDX_CKD / RISKLIMITS; RUN;

PROC PHREG DATA=SHIM.DIS_1_CVA_S_7_S3; CLASS CASE; MODEL DIFFMO_2*CVA(0)=DDX_COPD / RISKLIMITS; RUN;

PROC PHREG DATA=SHIM.DIS_1_CVA_S_7_S3; CLASS CASE; MODEL DIFFMO_2*CVA(0)=DDX_ASTH / RISKLIMITS; RUN;

PROC PHREG DATA=SHIM.DIS_1_CVA_S_7_S3; CLASS CASE; MODEL DIFFMO_2*CVA(0)=DDX_CAN / RISKLIMITS; RUN;

PROC PHREG DATA=SHIM.DIS_1_CVA_S_7_S3; CLASS CASE; MODEL DIFFMO_2*CVA(0)=CITY / RISKLIMITS; RUN;

PROC PHREG DATA=SHIM.DIS_1_CVA_S_7_S3; CLASS CASE CHA_NEW(REF='1') ; MODEL DIFFMO_2*CVA(0)=CHA_NEW / RISKLIMITS; RUN;

PROC PHREG DATA=SHIM.DIS_1_CVA_S_7_S3; CLASS CASE; MODEL DIFFMO_2*CVA(0)=INSUP / RISKLIMITS; RUN;

TITLE '====================== IHD ========================';

PROC PHREG DATA=SHIM.DIS_1_IHD_S_7_S3; CLASS CASE; MODEL DIFFMO_2*IHD(0)=CASE / RISKLIMITS; RUN;

PROC PHREG DATA=SHIM.DIS_1_IHD_S_7_S3; CLASS CASE; MODEL DIFFMO_2*IHD(0)=TTX_CHOL / RISKLIMITS; RUN;

PROC PHREG DATA=SHIM.DIS_1_IHD_S_7_S3; CLASS CASE; MODEL DIFFMO_2*IHD(0)=TTX_COA / RISKLIMITS; RUN;

PROC PHREG DATA=SHIM.DIS_1_IHD_S_7_S3; CLASS CASE; MODEL DIFFMO_2*IHD(0)=TTX_PLT / RISKLIMITS; RUN;

PROC PHREG DATA=SHIM.DIS_1_IHD_S_7_S3; CLASS CASE; MODEL DIFFMO_2*IHD(0)=TTX_ANT / RISKLIMITS; RUN;

PROC PHREG DATA=SHIM.DIS_1_IHD_S_7_S3; CLASS CASE; MODEL DIFFMO_2*IHD(0)=DDX_HTN / RISKLIMITS; RUN;

PROC PHREG DATA=SHIM.DIS_1_IHD_S_7_S3; CLASS CASE; MODEL DIFFMO_2*IHD(0)=DDX_LIV / RISKLIMITS; RUN;

PROC PHREG DATA=SHIM.DIS_1_IHD_S_7_S3; CLASS CASE; MODEL DIFFMO_2*IHD(0)=DDX_CKD / RISKLIMITS; RUN;

PROC PHREG DATA=SHIM.DIS_1_IHD_S_7_S3; CLASS CASE; MODEL DIFFMO_2*IHD(0)=DDX_COPD / RISKLIMITS; RUN;

PROC PHREG DATA=SHIM.DIS_1_IHD_S_7_S3; CLASS CASE; MODEL DIFFMO_2*IHD(0)=DDX_ASTH / RISKLIMITS; RUN;

PROC PHREG DATA=SHIM.DIS_1_IHD_S_7_S3; CLASS CASE; MODEL DIFFMO_2*IHD(0)=DDX_CAN / RISKLIMITS; RUN;

PROC PHREG DATA=SHIM.DIS_1_IHD_S_7_S3; CLASS CASE; MODEL DIFFMO_2*IHD(0)=CITY / RISKLIMITS; RUN;

PROC PHREG DATA=SHIM.DIS_1_IHD_S_7_S3; CLASS CASE CHA_NEW(REF='1'); MODEL DIFFMO_2*IHD(0)=CHA_NEW / RISKLIMITS; RUN;

PROC PHREG DATA=SHIM.DIS_1_IHD_S_7_S3; CLASS CASE; MODEL DIFFMO_2*IHD(0)=INSUP / RISKLIMITS; RUN;

TITLE '====================== DM ========================';

PROC PHREG DATA=SHIM.DIS_1_DM_S_7_S3; CLASS CASE; MODEL DIFFMO_2*DM(0)=CASE / RISKLIMITS; RUN;

PROC PHREG DATA=SHIM.DIS_1_DM_S_7_S3; CLASS CASE; MODEL DIFFMO_2*DM(0)=TTX_CHOL / RISKLIMITS; RUN;

PROC PHREG DATA=SHIM.DIS_1_DM_S_7_S3; CLASS CASE; MODEL DIFFMO_2*DM(0)=TTX_COA / RISKLIMITS; RUN;

PROC PHREG DATA=SHIM.DIS_1_DM_S_7_S3; CLASS CASE; MODEL DIFFMO_2*DM(0)=TTX_PLT / RISKLIMITS; RUN;

PROC PHREG DATA=SHIM.DIS_1_DM_S_7_S3; CLASS CASE; MODEL DIFFMO_2*DM(0)=TTX_ANT / RISKLIMITS; RUN;

PROC PHREG DATA=SHIM.DIS_1_DM_S_7_S3; CLASS CASE; MODEL DIFFMO_2*DM(0)=DDX_HTN / RISKLIMITS; RUN;

PROC PHREG DATA=SHIM.DIS_1_DM_S_7_S3; CLASS CASE; MODEL DIFFMO_2*DM(0)=DDX_LIV / RISKLIMITS; RUN;

PROC PHREG DATA=SHIM.DIS_1_DM_S_7_S3; CLASS CASE; MODEL DIFFMO_2*DM(0)=DDX_CKD / RISKLIMITS; RUN;

PROC PHREG DATA=SHIM.DIS_1_DM_S_7_S3; CLASS CASE; MODEL DIFFMO_2*DM(0)=DDX_COPD / RISKLIMITS; RUN;

PROC PHREG DATA=SHIM.DIS_1_DM_S_7_S3; CLASS CASE; MODEL DIFFMO_2*DM(0)=DDX_ASTH / RISKLIMITS; RUN;

PROC PHREG DATA=SHIM.DIS_1_DM_S_7_S3; CLASS CASE; MODEL DIFFMO_2*DM(0)=DDX_CAN / RISKLIMITS; RUN;

PROC PHREG DATA=SHIM.DIS_1_DM_S_7_S3; CLASS CASE; MODEL DIFFMO_2*DM(0)=CITY / RISKLIMITS; RUN;

PROC PHREG DATA=SHIM.DIS_1_DM_S_7_S3; CLASS CASE CHA_NEW(REF='1'); MODEL DIFFMO_2*DM(0)=CHA_NEW / RISKLIMITS; RUN;

PROC PHREG DATA=SHIM.DIS_1_DM_S_7_S3; CLASS CASE; MODEL DIFFMO_2*DM(0)=INSUP / RISKLIMITS; RUN;

TITLE '====================== MI ========================';

PROC PHREG DATA=SHIM.DIS_1_MI_S_7_S3; CLASS CASE; MODEL DIFFMO_2*MI(0)=CASE / RISKLIMITS; RUN;

PROC PHREG DATA=SHIM.DIS_1_MI_S_7_S3; CLASS CASE; MODEL DIFFMO_2*MI(0)=TTX_CHOL / RISKLIMITS; RUN;

PROC PHREG DATA=SHIM.DIS_1_MI_S_7_S3; CLASS CASE; MODEL DIFFMO_2*MI(0)=TTX_COA / RISKLIMITS; RUN;

PROC PHREG DATA=SHIM.DIS_1_MI_S_7_S3; CLASS CASE; MODEL DIFFMO_2*MI(0)=TTX_PLT / RISKLIMITS; RUN;

PROC PHREG DATA=SHIM.DIS_1_MI_S_7_S3; CLASS CASE; MODEL DIFFMO_2*MI(0)=TTX_ANT / RISKLIMITS; RUN;

PROC PHREG DATA=SHIM.DIS_1_MI_S_7_S3; CLASS CASE; MODEL DIFFMO_2*MI(0)=DDX_HTN / RISKLIMITS; RUN;

PROC PHREG DATA=SHIM.DIS_1_MI_S_7_S3; CLASS CASE; MODEL DIFFMO_2*MI(0)=DDX_LIV / RISKLIMITS; RUN;

PROC PHREG DATA=SHIM.DIS_1_MI_S_7_S3; CLASS CASE; MODEL DIFFMO_2*MI(0)=DDX_CKD / RISKLIMITS; RUN;

PROC PHREG DATA=SHIM.DIS_1_MI_S_7_S3; CLASS CASE; MODEL DIFFMO_2*MI(0)=DDX_COPD / RISKLIMITS; RUN;

PROC PHREG DATA=SHIM.DIS_1_MI_S_7_S3; CLASS CASE; MODEL DIFFMO_2*MI(0)=DDX_ASTH / RISKLIMITS; RUN;

PROC PHREG DATA=SHIM.DIS_1_MI_S_7_S3; CLASS CASE; MODEL DIFFMO_2*MI(0)=DDX_CAN / RISKLIMITS; RUN;

PROC PHREG DATA=SHIM.DIS_1_MI_S_7_S3; CLASS CASE; MODEL DIFFMO_2*MI(0)=CITY / RISKLIMITS; RUN;

PROC PHREG DATA=SHIM.DIS_1_MI_S_7_S3; CLASS CASE CHA_NEW(REF='1'); MODEL DIFFMO_2*MI(0)=CHA_NEW / RISKLIMITS; RUN;

PROC PHREG DATA=SHIM.DIS_1_MI_S_7_S3; CLASS CASE; MODEL DIFFMO_2*MI(0)=INSUP / RISKLIMITS; RUN;

TITLE '====================== ALZ ========================';

PROC PHREG DATA=SHIM.DIS_2_ALZ_S_7_S3; CLASS CASE; MODEL DIFFMO_2*ALZ(0)=CASE / RISKLIMITS; RUN;

PROC PHREG DATA=SHIM.DIS_2_ALZ_S_7_S3; CLASS CASE; MODEL DIFFMO_2*ALZ(0)=TTX_CHOL / RISKLIMITS; RUN;

PROC PHREG DATA=SHIM.DIS_2_ALZ_S_7_S3; CLASS CASE; MODEL DIFFMO_2*ALZ(0)=TTX_COA / RISKLIMITS; RUN;

PROC PHREG DATA=SHIM.DIS_2_ALZ_S_7_S3; CLASS CASE; MODEL DIFFMO_2*ALZ(0)=TTX_PLT / RISKLIMITS; RUN;

PROC PHREG DATA=SHIM.DIS_2_ALZ_S_7_S3; CLASS CASE; MODEL DIFFMO_2*ALZ(0)=TTX_ANT / RISKLIMITS; RUN;

PROC PHREG DATA=SHIM.DIS_2_ALZ_S_7_S3; CLASS CASE; MODEL DIFFMO_2*ALZ(0)=DDX_HTN / RISKLIMITS; RUN;

PROC PHREG DATA=SHIM.DIS_2_ALZ_S_7_S3; CLASS CASE; MODEL DIFFMO_2*ALZ(0)=DDX_DM / RISKLIMITS; RUN;

PROC PHREG DATA=SHIM.DIS_2_ALZ_S_7_S3; CLASS CASE; MODEL DIFFMO_2*ALZ(0)=CVD / RISKLIMITS; RUN;

PROC PHREG DATA=SHIM.DIS_2_ALZ_S_7_S3; CLASS CASE; MODEL DIFFMO_2*ALZ(0)=DDX_LIV / RISKLIMITS; RUN;

PROC PHREG DATA=SHIM.DIS_2_ALZ_S_7_S3; CLASS CASE; MODEL DIFFMO_2*ALZ(0)=DDX_CKD / RISKLIMITS; RUN;

PROC PHREG DATA=SHIM.DIS_2_ALZ_S_7_S3; CLASS CASE; MODEL DIFFMO_2*ALZ(0)=DDX_COPD / RISKLIMITS; RUN;

PROC PHREG DATA=SHIM.DIS_2_ALZ_S_7_S3; CLASS CASE; MODEL DIFFMO_2*ALZ(0)=DDX_ASTH / RISKLIMITS; RUN;

PROC PHREG DATA=SHIM.DIS_2_ALZ_S_7_S3; CLASS CASE; MODEL DIFFMO_2*ALZ(0)=DDX_CAN / RISKLIMITS; RUN;

PROC PHREG DATA=SHIM.DIS_2_ALZ_S_7_S3; CLASS CASE; MODEL DIFFMO_2*ALZ(0)=CITY / RISKLIMITS; RUN;

PROC PHREG DATA=SHIM.DIS_2_ALZ_S_7_S3; CLASS CASE CHA_NEW(REF='1'); MODEL DIFFMO_2*ALZ(0)=CHA_NEW / RISKLIMITS; RUN;

PROC PHREG DATA=SHIM.DIS_2_ALZ_S_7_S3; CLASS CASE; MODEL DIFFMO_2*ALZ(0)=INSUP / RISKLIMITS; RUN;

TITLE '====================== PKS ========================';

PROC PHREG DATA=SHIM.DIS_2_PKS_S_7_S3; CLASS CASE; MODEL DIFFMO_2*PKS(0)=CASE / RISKLIMITS; RUN;

PROC PHREG DATA=SHIM.DIS_2_PKS_S_7_S3; CLASS CASE; MODEL DIFFMO_2*PKS(0)=TTX_CHOL / RISKLIMITS; RUN;

PROC PHREG DATA=SHIM.DIS_2_PKS_S_7_S3; CLASS CASE; MODEL DIFFMO_2*PKS(0)=TTX_COA / RISKLIMITS; RUN;

PROC PHREG DATA=SHIM.DIS_2_PKS_S_7_S3; CLASS CASE; MODEL DIFFMO_2*PKS(0)=TTX_PLT / RISKLIMITS; RUN;

PROC PHREG DATA=SHIM.DIS_2_PKS_S_7_S3; CLASS CASE; MODEL DIFFMO_2*PKS(0)=TTX_ANT / RISKLIMITS; RUN;

PROC PHREG DATA=SHIM.DIS_2_PKS_S_7_S3; CLASS CASE; MODEL DIFFMO_2*PKS(0)=DDX_HTN / RISKLIMITS; RUN;

PROC PHREG DATA=SHIM.DIS_2_PKS_S_7_S3; CLASS CASE; MODEL DIFFMO_2*PKS(0)=DDX_DM / RISKLIMITS; RUN;

PROC PHREG DATA=SHIM.DIS_2_PKS_S_7_S3; CLASS CASE; MODEL DIFFMO_2*PKS(0)=CVD / RISKLIMITS; RUN;

PROC PHREG DATA=SHIM.DIS_2_PKS_S_7_S3; CLASS CASE; MODEL DIFFMO_2*PKS(0)=DDX_LIV / RISKLIMITS; RUN;

PROC PHREG DATA=SHIM.DIS_2_PKS_S_7_S3; CLASS CASE; MODEL DIFFMO_2*PKS(0)=DDX_CKD / RISKLIMITS; RUN;

PROC PHREG DATA=SHIM.DIS_2_PKS_S_7_S3; CLASS CASE; MODEL DIFFMO_2*PKS(0)=DDX_COPD / RISKLIMITS; RUN;

PROC PHREG DATA=SHIM.DIS_2_PKS_S_7_S3; CLASS CASE; MODEL DIFFMO_2*PKS(0)=DDX_ASTH / RISKLIMITS; RUN;

PROC PHREG DATA=SHIM.DIS_2_PKS_S_7_S3; CLASS CASE; MODEL DIFFMO_2*PKS(0)=DDX_CAN / RISKLIMITS; RUN;

PROC PHREG DATA=SHIM.DIS_2_PKS_S_7_S3; CLASS CASE; MODEL DIFFMO_2*PKS(0)=CITY / RISKLIMITS; RUN;

PROC PHREG DATA=SHIM.DIS_2_PKS_S_7_S3; CLASS CASE CHA_NEW(REF='1'); MODEL DIFFMO_2*PKS(0)=CHA_NEW / RISKLIMITS; RUN;

PROC PHREG DATA=SHIM.DIS_2_PKS_S_7_S3; CLASS CASE; MODEL DIFFMO_2*PKS(0)=INSUP / RISKLIMITS; RUN;

TITLE '*****************************SAGE = 4 ***************************';

TITLE '====================== CVA ========================';

PROC PHREG DATA=SHIM.DIS_1_CVA_S_7_S4; CLASS CASE; MODEL DIFFMO_2*CVA(0)=CASE / RISKLIMITS; RUN;

PROC PHREG DATA=SHIM.DIS_1_CVA_S_7_S4; CLASS CASE; MODEL DIFFMO_2*CVA(0)=TTX_CHOL / RISKLIMITS; RUN;

PROC PHREG DATA=SHIM.DIS_1_CVA_S_7_S4; CLASS CASE; MODEL DIFFMO_2*CVA(0)=TTX_COA / RISKLIMITS; RUN;

PROC PHREG DATA=SHIM.DIS_1_CVA_S_7_S4; CLASS CASE; MODEL DIFFMO_2*CVA(0)=TTX_PLT / RISKLIMITS; RUN;

PROC PHREG DATA=SHIM.DIS_1_CVA_S_7_S4; CLASS CASE; MODEL DIFFMO_2*CVA(0)=TTX_ANT / RISKLIMITS; RUN;

PROC PHREG DATA=SHIM.DIS_1_CVA_S_7_S4; CLASS CASE; MODEL DIFFMO_2*CVA(0)=DDX_HTN / RISKLIMITS; RUN;

PROC PHREG DATA=SHIM.DIS_1_CVA_S_7_S4; CLASS CASE; MODEL DIFFMO_2*CVA(0)=DDX_LIV / RISKLIMITS; RUN;

PROC PHREG DATA=SHIM.DIS_1_CVA_S_7_S4; CLASS CASE; MODEL DIFFMO_2*CVA(0)=DDX_CKD / RISKLIMITS; RUN;

PROC PHREG DATA=SHIM.DIS_1_CVA_S_7_S4; CLASS CASE; MODEL DIFFMO_2*CVA(0)=DDX_COPD / RISKLIMITS; RUN;

PROC PHREG DATA=SHIM.DIS_1_CVA_S_7_S4; CLASS CASE; MODEL DIFFMO_2*CVA(0)=DDX_ASTH / RISKLIMITS; RUN;

PROC PHREG DATA=SHIM.DIS_1_CVA_S_7_S4; CLASS CASE; MODEL DIFFMO_2*CVA(0)=DDX_CAN / RISKLIMITS; RUN;

PROC PHREG DATA=SHIM.DIS_1_CVA_S_7_S4; CLASS CASE; MODEL DIFFMO_2*CVA(0)=CITY / RISKLIMITS; RUN;

PROC PHREG DATA=SHIM.DIS_1_CVA_S_7_S4; CLASS CASE CHA_NEW(REF='1') ; MODEL DIFFMO_2*CVA(0)=CHA_NEW / RISKLIMITS; RUN;

PROC PHREG DATA=SHIM.DIS_1_CVA_S_7_S4; CLASS CASE; MODEL DIFFMO_2*CVA(0)=INSUP / RISKLIMITS; RUN;

TITLE '====================== IHD ========================';

PROC PHREG DATA=SHIM.DIS_1_IHD_S_7_S4; CLASS CASE; MODEL DIFFMO_2*IHD(0)=CASE / RISKLIMITS; RUN;

PROC PHREG DATA=SHIM.DIS_1_IHD_S_7_S4; CLASS CASE; MODEL DIFFMO_2*IHD(0)=TTX_CHOL / RISKLIMITS; RUN;

PROC PHREG DATA=SHIM.DIS_1_IHD_S_7_S4; CLASS CASE; MODEL DIFFMO_2*IHD(0)=TTX_COA / RISKLIMITS; RUN;

PROC PHREG DATA=SHIM.DIS_1_IHD_S_7_S4; CLASS CASE; MODEL DIFFMO_2*IHD(0)=TTX_PLT / RISKLIMITS; RUN;

PROC PHREG DATA=SHIM.DIS_1_IHD_S_7_S4; CLASS CASE; MODEL DIFFMO_2*IHD(0)=TTX_ANT / RISKLIMITS; RUN;

PROC PHREG DATA=SHIM.DIS_1_IHD_S_7_S4; CLASS CASE; MODEL DIFFMO_2*IHD(0)=DDX_HTN / RISKLIMITS; RUN;

PROC PHREG DATA=SHIM.DIS_1_IHD_S_7_S4; CLASS CASE; MODEL DIFFMO_2*IHD(0)=DDX_LIV / RISKLIMITS; RUN;

PROC PHREG DATA=SHIM.DIS_1_IHD_S_7_S4; CLASS CASE; MODEL DIFFMO_2*IHD(0)=DDX_CKD / RISKLIMITS; RUN;

PROC PHREG DATA=SHIM.DIS_1_IHD_S_7_S4; CLASS CASE; MODEL DIFFMO_2*IHD(0)=DDX_COPD / RISKLIMITS; RUN;

PROC PHREG DATA=SHIM.DIS_1_IHD_S_7_S4; CLASS CASE; MODEL DIFFMO_2*IHD(0)=DDX_ASTH / RISKLIMITS; RUN;

PROC PHREG DATA=SHIM.DIS_1_IHD_S_7_S4; CLASS CASE; MODEL DIFFMO_2*IHD(0)=DDX_CAN / RISKLIMITS; RUN;

PROC PHREG DATA=SHIM.DIS_1_IHD_S_7_S4; CLASS CASE; MODEL DIFFMO_2*IHD(0)=CITY / RISKLIMITS; RUN;

PROC PHREG DATA=SHIM.DIS_1_IHD_S_7_S4; CLASS CASE CHA_NEW(REF='1'); MODEL DIFFMO_2*IHD(0)=CHA_NEW / RISKLIMITS; RUN;

PROC PHREG DATA=SHIM.DIS_1_IHD_S_7_S4; CLASS CASE; MODEL DIFFMO_2*IHD(0)=INSUP / RISKLIMITS; RUN;

TITLE '====================== DM ========================';

PROC PHREG DATA=SHIM.DIS_1_DM_S_7_S4; CLASS CASE; MODEL DIFFMO_2*DM(0)=CASE / RISKLIMITS; RUN;

PROC PHREG DATA=SHIM.DIS_1_DM_S_7_S4; CLASS CASE; MODEL DIFFMO_2*DM(0)=TTX_CHOL / RISKLIMITS; RUN;

PROC PHREG DATA=SHIM.DIS_1_DM_S_7_S4; CLASS CASE; MODEL DIFFMO_2*DM(0)=TTX_COA / RISKLIMITS; RUN;

PROC PHREG DATA=SHIM.DIS_1_DM_S_7_S4; CLASS CASE; MODEL DIFFMO_2*DM(0)=TTX_PLT / RISKLIMITS; RUN;

PROC PHREG DATA=SHIM.DIS_1_DM_S_7_S4; CLASS CASE; MODEL DIFFMO_2*DM(0)=TTX_ANT / RISKLIMITS; RUN;

PROC PHREG DATA=SHIM.DIS_1_DM_S_7_S4; CLASS CASE; MODEL DIFFMO_2*DM(0)=DDX_HTN / RISKLIMITS; RUN;

PROC PHREG DATA=SHIM.DIS_1_DM_S_7_S4; CLASS CASE; MODEL DIFFMO_2*DM(0)=DDX_LIV / RISKLIMITS; RUN;

PROC PHREG DATA=SHIM.DIS_1_DM_S_7_S4; CLASS CASE; MODEL DIFFMO_2*DM(0)=DDX_CKD / RISKLIMITS; RUN;

PROC PHREG DATA=SHIM.DIS_1_DM_S_7_S4; CLASS CASE; MODEL DIFFMO_2*DM(0)=DDX_COPD / RISKLIMITS; RUN;

PROC PHREG DATA=SHIM.DIS_1_DM_S_7_S4; CLASS CASE; MODEL DIFFMO_2*DM(0)=DDX_ASTH / RISKLIMITS; RUN;

PROC PHREG DATA=SHIM.DIS_1_DM_S_7_S4; CLASS CASE; MODEL DIFFMO_2*DM(0)=DDX_CAN / RISKLIMITS; RUN;

PROC PHREG DATA=SHIM.DIS_1_DM_S_7_S4; CLASS CASE; MODEL DIFFMO_2*DM(0)=CITY / RISKLIMITS; RUN;

PROC PHREG DATA=SHIM.DIS_1_DM_S_7_S4; CLASS CASE CHA_NEW(REF='1'); MODEL DIFFMO_2*DM(0)=CHA_NEW / RISKLIMITS; RUN;

PROC PHREG DATA=SHIM.DIS_1_DM_S_7_S4; CLASS CASE; MODEL DIFFMO_2*DM(0)=INSUP / RISKLIMITS; RUN;

TITLE '====================== MI ========================';

PROC PHREG DATA=SHIM.DIS_1_MI_S_7_S4; CLASS CASE; MODEL DIFFMO_2*MI(0)=CASE / RISKLIMITS; RUN;

PROC PHREG DATA=SHIM.DIS_1_MI_S_7_S4; CLASS CASE; MODEL DIFFMO_2*MI(0)=TTX_CHOL / RISKLIMITS; RUN;

PROC PHREG DATA=SHIM.DIS_1_MI_S_7_S4; CLASS CASE; MODEL DIFFMO_2*MI(0)=TTX_COA / RISKLIMITS; RUN;

PROC PHREG DATA=SHIM.DIS_1_MI_S_7_S4; CLASS CASE; MODEL DIFFMO_2*MI(0)=TTX_PLT / RISKLIMITS; RUN;

PROC PHREG DATA=SHIM.DIS_1_MI_S_7_S4; CLASS CASE; MODEL DIFFMO_2*MI(0)=TTX_ANT / RISKLIMITS; RUN;

PROC PHREG DATA=SHIM.DIS_1_MI_S_7_S4; CLASS CASE; MODEL DIFFMO_2*MI(0)=DDX_HTN / RISKLIMITS; RUN;

PROC PHREG DATA=SHIM.DIS_1_MI_S_7_S4; CLASS CASE; MODEL DIFFMO_2*MI(0)=DDX_LIV / RISKLIMITS; RUN;

PROC PHREG DATA=SHIM.DIS_1_MI_S_7_S4; CLASS CASE; MODEL DIFFMO_2*MI(0)=DDX_CKD / RISKLIMITS; RUN;

PROC PHREG DATA=SHIM.DIS_1_MI_S_7_S4; CLASS CASE; MODEL DIFFMO_2*MI(0)=DDX_COPD / RISKLIMITS; RUN;

PROC PHREG DATA=SHIM.DIS_1_MI_S_7_S4; CLASS CASE; MODEL DIFFMO_2*MI(0)=DDX_ASTH / RISKLIMITS; RUN;

PROC PHREG DATA=SHIM.DIS_1_MI_S_7_S4; CLASS CASE; MODEL DIFFMO_2*MI(0)=DDX_CAN / RISKLIMITS; RUN;

PROC PHREG DATA=SHIM.DIS_1_MI_S_7_S4; CLASS CASE; MODEL DIFFMO_2*MI(0)=CITY / RISKLIMITS; RUN;

PROC PHREG DATA=SHIM.DIS_1_MI_S_7_S4; CLASS CASE CHA_NEW(REF='1'); MODEL DIFFMO_2*MI(0)=CHA_NEW / RISKLIMITS; RUN;

PROC PHREG DATA=SHIM.DIS_1_MI_S_7_S4; CLASS CASE; MODEL DIFFMO_2*MI(0)=INSUP / RISKLIMITS; RUN;

TITLE '====================== ALZ ========================';

PROC PHREG DATA=SHIM.DIS_2_ALZ_S_7_S4; CLASS CASE; MODEL DIFFMO_2*ALZ(0)=CASE / RISKLIMITS; RUN;

PROC PHREG DATA=SHIM.DIS_2_ALZ_S_7_S4; CLASS CASE; MODEL DIFFMO_2*ALZ(0)=TTX_CHOL / RISKLIMITS; RUN;

PROC PHREG DATA=SHIM.DIS_2_ALZ_S_7_S4; CLASS CASE; MODEL DIFFMO_2*ALZ(0)=TTX_COA / RISKLIMITS; RUN;

PROC PHREG DATA=SHIM.DIS_2_ALZ_S_7_S4; CLASS CASE; MODEL DIFFMO_2*ALZ(0)=TTX_PLT / RISKLIMITS; RUN;

PROC PHREG DATA=SHIM.DIS_2_ALZ_S_7_S4; CLASS CASE; MODEL DIFFMO_2*ALZ(0)=TTX_ANT / RISKLIMITS; RUN;

PROC PHREG DATA=SHIM.DIS_2_ALZ_S_7_S4; CLASS CASE; MODEL DIFFMO_2*ALZ(0)=DDX_HTN / RISKLIMITS; RUN;

PROC PHREG DATA=SHIM.DIS_2_ALZ_S_7_S4; CLASS CASE; MODEL DIFFMO_2*ALZ(0)=DDX_DM / RISKLIMITS; RUN;

PROC PHREG DATA=SHIM.DIS_2_ALZ_S_7_S4; CLASS CASE; MODEL DIFFMO_2*ALZ(0)=CVD / RISKLIMITS; RUN;

PROC PHREG DATA=SHIM.DIS_2_ALZ_S_7_S4; CLASS CASE; MODEL DIFFMO_2*ALZ(0)=DDX_LIV / RISKLIMITS; RUN;

PROC PHREG DATA=SHIM.DIS_2_ALZ_S_7_S4; CLASS CASE; MODEL DIFFMO_2*ALZ(0)=DDX_CKD / RISKLIMITS; RUN;

PROC PHREG DATA=SHIM.DIS_2_ALZ_S_7_S4; CLASS CASE; MODEL DIFFMO_2*ALZ(0)=DDX_COPD / RISKLIMITS; RUN;

PROC PHREG DATA=SHIM.DIS_2_ALZ_S_7_S4; CLASS CASE; MODEL DIFFMO_2*ALZ(0)=DDX_ASTH / RISKLIMITS; RUN;

PROC PHREG DATA=SHIM.DIS_2_ALZ_S_7_S4; CLASS CASE; MODEL DIFFMO_2*ALZ(0)=DDX_CAN / RISKLIMITS; RUN;

PROC PHREG DATA=SHIM.DIS_2_ALZ_S_7_S4; CLASS CASE; MODEL DIFFMO_2*ALZ(0)=CITY / RISKLIMITS; RUN;

PROC PHREG DATA=SHIM.DIS_2_ALZ_S_7_S4; CLASS CASE CHA_NEW(REF='1'); MODEL DIFFMO_2*ALZ(0)=CHA_NEW / RISKLIMITS; RUN;

PROC PHREG DATA=SHIM.DIS_2_ALZ_S_7_S4; CLASS CASE; MODEL DIFFMO_2*ALZ(0)=INSUP / RISKLIMITS; RUN;

TITLE '====================== PKS ========================';

PROC PHREG DATA=SHIM.DIS_2_PKS_S_7_S4; CLASS CASE; MODEL DIFFMO_2*PKS(0)=CASE / RISKLIMITS; RUN;

PROC PHREG DATA=SHIM.DIS_2_PKS_S_7_S4; CLASS CASE; MODEL DIFFMO_2*PKS(0)=TTX_CHOL / RISKLIMITS; RUN;

PROC PHREG DATA=SHIM.DIS_2_PKS_S_7_S4; CLASS CASE; MODEL DIFFMO_2*PKS(0)=TTX_COA / RISKLIMITS; RUN;

PROC PHREG DATA=SHIM.DIS_2_PKS_S_7_S4; CLASS CASE; MODEL DIFFMO_2*PKS(0)=TTX_PLT / RISKLIMITS; RUN;

PROC PHREG DATA=SHIM.DIS_2_PKS_S_7_S4; CLASS CASE; MODEL DIFFMO_2*PKS(0)=TTX_ANT / RISKLIMITS; RUN;

PROC PHREG DATA=SHIM.DIS_2_PKS_S_7_S4; CLASS CASE; MODEL DIFFMO_2*PKS(0)=DDX_HTN / RISKLIMITS; RUN;

PROC PHREG DATA=SHIM.DIS_2_PKS_S_7_S4; CLASS CASE; MODEL DIFFMO_2*PKS(0)=DDX_DM / RISKLIMITS; RUN;

PROC PHREG DATA=SHIM.DIS_2_PKS_S_7_S4; CLASS CASE; MODEL DIFFMO_2*PKS(0)=CVD / RISKLIMITS; RUN;

PROC PHREG DATA=SHIM.DIS_2_PKS_S_7_S4; CLASS CASE; MODEL DIFFMO_2*PKS(0)=DDX_LIV / RISKLIMITS; RUN;

PROC PHREG DATA=SHIM.DIS_2_PKS_S_7_S4; CLASS CASE; MODEL DIFFMO_2*PKS(0)=DDX_CKD / RISKLIMITS; RUN;

PROC PHREG DATA=SHIM.DIS_2_PKS_S_7_S4; CLASS CASE; MODEL DIFFMO_2*PKS(0)=DDX_COPD / RISKLIMITS; RUN;

PROC PHREG DATA=SHIM.DIS_2_PKS_S_7_S4; CLASS CASE; MODEL DIFFMO_2*PKS(0)=DDX_ASTH / RISKLIMITS; RUN;

PROC PHREG DATA=SHIM.DIS_2_PKS_S_7_S4; CLASS CASE; MODEL DIFFMO_2*PKS(0)=DDX_CAN / RISKLIMITS; RUN;

PROC PHREG DATA=SHIM.DIS_2_PKS_S_7_S4; CLASS CASE; MODEL DIFFMO_2*PKS(0)=CITY / RISKLIMITS; RUN;

PROC PHREG DATA=SHIM.DIS_2_PKS_S_7_S4; CLASS CASE CHA_NEW(REF='1'); MODEL DIFFMO_2*PKS(0)=CHA_NEW / RISKLIMITS; RUN;

PROC PHREG DATA=SHIM.DIS_2_PKS_S_7_S4; CLASS CASE; MODEL DIFFMO_2*PKS(0)=INSUP / RISKLIMITS; RUN;

TITLE '*****************************NAGE = 0 ***************************';

TITLE '====================== CVA ========================';

PROC PHREG DATA=SHIM.DIS_1_CVA_S_7_N0; CLASS CASE; MODEL DIFFMO_2*CVA(0)=CASE / RISKLIMITS; RUN;

PROC PHREG DATA=SHIM.DIS_1_CVA_S_7_N0; CLASS CASE; MODEL DIFFMO_2*CVA(0)=TTX_CHOL / RISKLIMITS; RUN;

PROC PHREG DATA=SHIM.DIS_1_CVA_S_7_N0; CLASS CASE; MODEL DIFFMO_2*CVA(0)=TTX_COA / RISKLIMITS; RUN;

PROC PHREG DATA=SHIM.DIS_1_CVA_S_7_N0; CLASS CASE; MODEL DIFFMO_2*CVA(0)=TTX_PLT / RISKLIMITS; RUN;

PROC PHREG DATA=SHIM.DIS_1_CVA_S_7_N0; CLASS CASE; MODEL DIFFMO_2*CVA(0)=TTX_ANT / RISKLIMITS; RUN;

PROC PHREG DATA=SHIM.DIS_1_CVA_S_7_N0; CLASS CASE; MODEL DIFFMO_2*CVA(0)=DDX_HTN / RISKLIMITS; RUN;

PROC PHREG DATA=SHIM.DIS_1_CVA_S_7_N0; CLASS CASE; MODEL DIFFMO_2*CVA(0)=DDX_LIV / RISKLIMITS; RUN;

PROC PHREG DATA=SHIM.DIS_1_CVA_S_7_N0; CLASS CASE; MODEL DIFFMO_2*CVA(0)=DDX_CKD / RISKLIMITS; RUN;

PROC PHREG DATA=SHIM.DIS_1_CVA_S_7_N0; CLASS CASE; MODEL DIFFMO_2*CVA(0)=DDX_COPD / RISKLIMITS; RUN;

PROC PHREG DATA=SHIM.DIS_1_CVA_S_7_N0; CLASS CASE; MODEL DIFFMO_2*CVA(0)=DDX_ASTH / RISKLIMITS; RUN;

PROC PHREG DATA=SHIM.DIS_1_CVA_S_7_N0; CLASS CASE; MODEL DIFFMO_2*CVA(0)=DDX_CAN / RISKLIMITS; RUN;

PROC PHREG DATA=SHIM.DIS_1_CVA_S_7_N0; CLASS CASE; MODEL DIFFMO_2*CVA(0)=CITY / RISKLIMITS; RUN;

PROC PHREG DATA=SHIM.DIS_1_CVA_S_7_N0; CLASS CASE CHA_NEW(REF='1') ; MODEL DIFFMO_2*CVA(0)=CHA_NEW / RISKLIMITS; RUN;

PROC PHREG DATA=SHIM.DIS_1_CVA_S_7_N0; CLASS CASE; MODEL DIFFMO_2*CVA(0)=INSUP / RISKLIMITS; RUN;

TITLE '====================== IHD ========================';

PROC PHREG DATA=SHIM.DIS_1_IHD_S_7_N0; CLASS CASE; MODEL DIFFMO_2*IHD(0)=CASE / RISKLIMITS; RUN;

PROC PHREG DATA=SHIM.DIS_1_IHD_S_7_N0; CLASS CASE; MODEL DIFFMO_2*IHD(0)=TTX_CHOL / RISKLIMITS; RUN;

PROC PHREG DATA=SHIM.DIS_1_IHD_S_7_N0; CLASS CASE; MODEL DIFFMO_2*IHD(0)=TTX_COA / RISKLIMITS; RUN;

PROC PHREG DATA=SHIM.DIS_1_IHD_S_7_N0; CLASS CASE; MODEL DIFFMO_2*IHD(0)=TTX_PLT / RISKLIMITS; RUN;

PROC PHREG DATA=SHIM.DIS_1_IHD_S_7_N0; CLASS CASE; MODEL DIFFMO_2*IHD(0)=TTX_ANT / RISKLIMITS; RUN;

PROC PHREG DATA=SHIM.DIS_1_IHD_S_7_N0; CLASS CASE; MODEL DIFFMO_2*IHD(0)=DDX_HTN / RISKLIMITS; RUN;

PROC PHREG DATA=SHIM.DIS_1_IHD_S_7_N0; CLASS CASE; MODEL DIFFMO_2*IHD(0)=DDX_LIV / RISKLIMITS; RUN;

PROC PHREG DATA=SHIM.DIS_1_IHD_S_7_N0; CLASS CASE; MODEL DIFFMO_2*IHD(0)=DDX_CKD / RISKLIMITS; RUN;

PROC PHREG DATA=SHIM.DIS_1_IHD_S_7_N0; CLASS CASE; MODEL DIFFMO_2*IHD(0)=DDX_COPD / RISKLIMITS; RUN;

PROC PHREG DATA=SHIM.DIS_1_IHD_S_7_N0; CLASS CASE; MODEL DIFFMO_2*IHD(0)=DDX_ASTH / RISKLIMITS; RUN;

PROC PHREG DATA=SHIM.DIS_1_IHD_S_7_N0; CLASS CASE; MODEL DIFFMO_2*IHD(0)=DDX_CAN / RISKLIMITS; RUN;

PROC PHREG DATA=SHIM.DIS_1_IHD_S_7_N0; CLASS CASE; MODEL DIFFMO_2*IHD(0)=CITY / RISKLIMITS; RUN;

PROC PHREG DATA=SHIM.DIS_1_IHD_S_7_N0; CLASS CASE CHA_NEW(REF='1'); MODEL DIFFMO_2*IHD(0)=CHA_NEW / RISKLIMITS; RUN;

PROC PHREG DATA=SHIM.DIS_1_IHD_S_7_N0; CLASS CASE; MODEL DIFFMO_2*IHD(0)=INSUP / RISKLIMITS; RUN;

TITLE '====================== DM =======================';

PROC PHREG DATA=SHIM.DIS_1_DM_S_7_N0; CLASS CASE; MODEL DIFFMO_2*DM(0)=CASE / RISKLIMITS; RUN;

PROC PHREG DATA=SHIM.DIS_1_DM_S_7_N0; CLASS CASE; MODEL DIFFMO_2*DM(0)=TTX_CHOL / RISKLIMITS; RUN;

PROC PHREG DATA=SHIM.DIS_1_DM_S_7_N0; CLASS CASE; MODEL DIFFMO_2*DM(0)=TTX_COA / RISKLIMITS; RUN;

PROC PHREG DATA=SHIM.DIS_1_DM_S_7_N0; CLASS CASE; MODEL DIFFMO_2*DM(0)=TTX_PLT / RISKLIMITS; RUN;

PROC PHREG DATA=SHIM.DIS_1_DM_S_7_N0; CLASS CASE; MODEL DIFFMO_2*DM(0)=TTX_ANT / RISKLIMITS; RUN;

PROC PHREG DATA=SHIM.DIS_1_DM_S_7_N0; CLASS CASE; MODEL DIFFMO_2*DM(0)=DDX_HTN / RISKLIMITS; RUN;

PROC PHREG DATA=SHIM.DIS_1_DM_S_7_N0; CLASS CASE; MODEL DIFFMO_2*DM(0)=DDX_LIV / RISKLIMITS; RUN;

PROC PHREG DATA=SHIM.DIS_1_DM_S_7_N0; CLASS CASE; MODEL DIFFMO_2*DM(0)=DDX_CKD / RISKLIMITS; RUN;

PROC PHREG DATA=SHIM.DIS_1_DM_S_7_N0; CLASS CASE; MODEL DIFFMO_2*DM(0)=DDX_COPD / RISKLIMITS; RUN;

PROC PHREG DATA=SHIM.DIS_1_DM_S_7_N0; CLASS CASE; MODEL DIFFMO_2*DM(0)=DDX_ASTH / RISKLIMITS; RUN;

PROC PHREG DATA=SHIM.DIS_1_DM_S_7_N0; CLASS CASE; MODEL DIFFMO_2*DM(0)=DDX_CAN / RISKLIMITS; RUN;

PROC PHREG DATA=SHIM.DIS_1_DM_S_7_N0; CLASS CASE; MODEL DIFFMO_2*DM(0)=CITY / RISKLIMITS; RUN;

PROC PHREG DATA=SHIM.DIS_1_DM_S_7_N0; CLASS CASE CHA_NEW(REF='1'); MODEL DIFFMO_2*DM(0)=CHA_NEW / RISKLIMITS; RUN;

PROC PHREG DATA=SHIM.DIS_1_DM_S_7_N0; CLASS CASE; MODEL DIFFMO_2*DM(0)=INSUP / RISKLIMITS; RUN;

TITLE '====================== MI ========================';

PROC PHREG DATA=SHIM.DIS_1_MI_S_7_N0; CLASS CASE; MODEL DIFFMO_2*MI(0)=CASE / RISKLIMITS; RUN;

PROC PHREG DATA=SHIM.DIS_1_MI_S_7_N0; CLASS CASE; MODEL DIFFMO_2*MI(0)=TTX_CHOL / RISKLIMITS; RUN;

PROC PHREG DATA=SHIM.DIS_1_MI_S_7_N0; CLASS CASE; MODEL DIFFMO_2*MI(0)=TTX_COA / RISKLIMITS; RUN;

PROC PHREG DATA=SHIM.DIS_1_MI_S_7_N0; CLASS CASE; MODEL DIFFMO_2*MI(0)=TTX_PLT / RISKLIMITS; RUN;

PROC PHREG DATA=SHIM.DIS_1_MI_S_7_N0; CLASS CASE; MODEL DIFFMO_2*MI(0)=TTX_ANT / RISKLIMITS; RUN;

PROC PHREG DATA=SHIM.DIS_1_MI_S_7_N0; CLASS CASE; MODEL DIFFMO_2*MI(0)=DDX_HTN / RISKLIMITS; RUN;

PROC PHREG DATA=SHIM.DIS_1_MI_S_7_N0; CLASS CASE; MODEL DIFFMO_2*MI(0)=DDX_LIV / RISKLIMITS; RUN;

PROC PHREG DATA=SHIM.DIS_1_MI_S_7_N0; CLASS CASE; MODEL DIFFMO_2*MI(0)=DDX_CKD / RISKLIMITS; RUN;

PROC PHREG DATA=SHIM.DIS_1_MI_S_7_N0; CLASS CASE; MODEL DIFFMO_2*MI(0)=DDX_COPD / RISKLIMITS; RUN;

PROC PHREG DATA=SHIM.DIS_1_MI_S_7_N0; CLASS CASE; MODEL DIFFMO_2*MI(0)=DDX_ASTH / RISKLIMITS; RUN;

PROC PHREG DATA=SHIM.DIS_1_MI_S_7_N0; CLASS CASE; MODEL DIFFMO_2*MI(0)=DDX_CAN / RISKLIMITS; RUN;

PROC PHREG DATA=SHIM.DIS_1_MI_S_7_N0; CLASS CASE; MODEL DIFFMO_2*MI(0)=CITY / RISKLIMITS; RUN;

PROC PHREG DATA=SHIM.DIS_1_MI_S_7_N0; CLASS CASE CHA_NEW(REF='1'); MODEL DIFFMO_2*MI(0)=CHA_NEW / RISKLIMITS; RUN;

PROC PHREG DATA=SHIM.DIS_1_MI_S_7_N0; CLASS CASE; MODEL DIFFMO_2*MI(0)=INSUP / RISKLIMITS; RUN;

TITLE '====================== ALZ ========================';

PROC PHREG DATA=SHIM.DIS_2_ALZ_S_7_N0; CLASS CASE; MODEL DIFFMO_2*ALZ(0)=CASE / RISKLIMITS; RUN;

PROC PHREG DATA=SHIM.DIS_2_ALZ_S_7_N0; CLASS CASE; MODEL DIFFMO_2*ALZ(0)=TTX_CHOL / RISKLIMITS; RUN;

PROC PHREG DATA=SHIM.DIS_2_ALZ_S_7_N0; CLASS CASE; MODEL DIFFMO_2*ALZ(0)=TTX_COA / RISKLIMITS; RUN;

PROC PHREG DATA=SHIM.DIS_2_ALZ_S_7_N0; CLASS CASE; MODEL DIFFMO_2*ALZ(0)=TTX_PLT / RISKLIMITS; RUN;

PROC PHREG DATA=SHIM.DIS_2_ALZ_S_7_N0; CLASS CASE; MODEL DIFFMO_2*ALZ(0)=TTX_ANT / RISKLIMITS; RUN;

PROC PHREG DATA=SHIM.DIS_2_ALZ_S_7_N0; CLASS CASE; MODEL DIFFMO_2*ALZ(0)=DDX_HTN / RISKLIMITS; RUN;

PROC PHREG DATA=SHIM.DIS_2_ALZ_S_7_N0; CLASS CASE; MODEL DIFFMO_2*ALZ(0)=DDX_DM / RISKLIMITS; RUN;

PROC PHREG DATA=SHIM.DIS_2_ALZ_S_7_N0; CLASS CASE; MODEL DIFFMO_2*ALZ(0)=CVD / RISKLIMITS; RUN;

PROC PHREG DATA=SHIM.DIS_2_ALZ_S_7_N0; CLASS CASE; MODEL DIFFMO_2*ALZ(0)=DDX_LIV / RISKLIMITS; RUN;

PROC PHREG DATA=SHIM.DIS_2_ALZ_S_7_N0; CLASS CASE; MODEL DIFFMO_2*ALZ(0)=DDX_CKD / RISKLIMITS; RUN;

PROC PHREG DATA=SHIM.DIS_2_ALZ_S_7_N0; CLASS CASE; MODEL DIFFMO_2*ALZ(0)=DDX_COPD / RISKLIMITS; RUN;

PROC PHREG DATA=SHIM.DIS_2_ALZ_S_7_N0; CLASS CASE; MODEL DIFFMO_2*ALZ(0)=DDX_ASTH / RISKLIMITS; RUN;

PROC PHREG DATA=SHIM.DIS_2_ALZ_S_7_N0; CLASS CASE; MODEL DIFFMO_2*ALZ(0)=DDX_CAN / RISKLIMITS; RUN;

PROC PHREG DATA=SHIM.DIS_2_ALZ_S_7_N0; CLASS CASE; MODEL DIFFMO_2*ALZ(0)=CITY / RISKLIMITS; RUN;

PROC PHREG DATA=SHIM.DIS_2_ALZ_S_7_N0; CLASS CASE CHA_NEW(REF='1'); MODEL DIFFMO_2*ALZ(0)=CHA_NEW / RISKLIMITS; RUN;

PROC PHREG DATA=SHIM.DIS_2_ALZ_S_7_N0; CLASS CASE; MODEL DIFFMO_2*ALZ(0)=INSUP / RISKLIMITS; RUN;

TITLE '====================== PKS ========================';

PROC PHREG DATA=SHIM.DIS_2_PKS_S_7_N0; CLASS CASE; MODEL DIFFMO_2*PKS(0)=CASE / RISKLIMITS; RUN;

PROC PHREG DATA=SHIM.DIS_2_PKS_S_7_N0; CLASS CASE; MODEL DIFFMO_2*PKS(0)=TTX_CHOL / RISKLIMITS; RUN;

PROC PHREG DATA=SHIM.DIS_2_PKS_S_7_N0; CLASS CASE; MODEL DIFFMO_2*PKS(0)=TTX_COA / RISKLIMITS; RUN;

PROC PHREG DATA=SHIM.DIS_2_PKS_S_7_N0; CLASS CASE; MODEL DIFFMO_2*PKS(0)=TTX_PLT / RISKLIMITS; RUN;

PROC PHREG DATA=SHIM.DIS_2_PKS_S_7_N0; CLASS CASE; MODEL DIFFMO_2*PKS(0)=TTX_ANT / RISKLIMITS; RUN;

PROC PHREG DATA=SHIM.DIS_2_PKS_S_7_N0; CLASS CASE; MODEL DIFFMO_2*PKS(0)=DDX_HTN / RISKLIMITS; RUN;

PROC PHREG DATA=SHIM.DIS_2_PKS_S_7_N0; CLASS CASE; MODEL DIFFMO_2*PKS(0)=DDX_DM / RISKLIMITS; RUN;

PROC PHREG DATA=SHIM.DIS_2_PKS_S_7_N0; CLASS CASE; MODEL DIFFMO_2*PKS(0)=CVD / RISKLIMITS; RUN;

PROC PHREG DATA=SHIM.DIS_2_PKS_S_7_N0; CLASS CASE; MODEL DIFFMO_2*PKS(0)=DDX_LIV / RISKLIMITS; RUN;

PROC PHREG DATA=SHIM.DIS_2_PKS_S_7_N0; CLASS CASE; MODEL DIFFMO_2*PKS(0)=DDX_CKD / RISKLIMITS; RUN;

PROC PHREG DATA=SHIM.DIS_2_PKS_S_7_N0; CLASS CASE; MODEL DIFFMO_2*PKS(0)=DDX_COPD / RISKLIMITS; RUN;

PROC PHREG DATA=SHIM.DIS_2_PKS_S_7_N0; CLASS CASE; MODEL DIFFMO_2*PKS(0)=DDX_ASTH / RISKLIMITS; RUN;

PROC PHREG DATA=SHIM.DIS_2_PKS_S_7_N0; CLASS CASE; MODEL DIFFMO_2*PKS(0)=DDX_CAN / RISKLIMITS; RUN;

PROC PHREG DATA=SHIM.DIS_2_PKS_S_7_N0; CLASS CASE; MODEL DIFFMO_2*PKS(0)=CITY / RISKLIMITS; RUN;

PROC PHREG DATA=SHIM.DIS_2_PKS_S_7_N0; CLASS CASE CHA_NEW(REF='1'); MODEL DIFFMO_2*PKS(0)=CHA_NEW / RISKLIMITS; RUN;

PROC PHREG DATA=SHIM.DIS_2_PKS_S_7_N0; CLASS CASE; MODEL DIFFMO_2*PKS(0)=INSUP / RISKLIMITS; RUN;

TITLE '*****************************NAGE = 1 ***************************';

TITLE '====================== CVA ========================';

PROC PHREG DATA=SHIM.DIS_1_CVA_S_7_N1; CLASS CASE; MODEL DIFFMO_2*CVA(0)=CASE / RISKLIMITS; RUN;

PROC PHREG DATA=SHIM.DIS_1_CVA_S_7_N1; CLASS CASE; MODEL DIFFMO_2*CVA(0)=TTX_CHOL / RISKLIMITS; RUN;

PROC PHREG DATA=SHIM.DIS_1_CVA_S_7_N1; CLASS CASE; MODEL DIFFMO_2*CVA(0)=TTX_COA / RISKLIMITS; RUN;

PROC PHREG DATA=SHIM.DIS_1_CVA_S_7_N1; CLASS CASE; MODEL DIFFMO_2*CVA(0)=TTX_PLT / RISKLIMITS; RUN;

PROC PHREG DATA=SHIM.DIS_1_CVA_S_7_N1; CLASS CASE; MODEL DIFFMO_2*CVA(0)=TTX_ANT / RISKLIMITS; RUN;

PROC PHREG DATA=SHIM.DIS_1_CVA_S_7_N1; CLASS CASE; MODEL DIFFMO_2*CVA(0)=DDX_HTN / RISKLIMITS; RUN;

PROC PHREG DATA=SHIM.DIS_1_CVA_S_7_N1; CLASS CASE; MODEL DIFFMO_2*CVA(0)=DDX_LIV / RISKLIMITS; RUN;

PROC PHREG DATA=SHIM.DIS_1_CVA_S_7_N1; CLASS CASE; MODEL DIFFMO_2*CVA(0)=DDX_CKD / RISKLIMITS; RUN;

PROC PHREG DATA=SHIM.DIS_1_CVA_S_7_N1; CLASS CASE; MODEL DIFFMO_2*CVA(0)=DDX_COPD / RISKLIMITS; RUN;

PROC PHREG DATA=SHIM.DIS_1_CVA_S_7_N1; CLASS CASE; MODEL DIFFMO_2*CVA(0)=DDX_ASTH / RISKLIMITS; RUN;

PROC PHREG DATA=SHIM.DIS_1_CVA_S_7_N1; CLASS CASE; MODEL DIFFMO_2*CVA(0)=DDX_CAN / RISKLIMITS; RUN;

PROC PHREG DATA=SHIM.DIS_1_CVA_S_7_N1; CLASS CASE; MODEL DIFFMO_2*CVA(0)=CITY / RISKLIMITS; RUN;

PROC PHREG DATA=SHIM.DIS_1_CVA_S_7_N1; CLASS CASE CHA_NEW(REF='1') ; MODEL DIFFMO_2*CVA(0)=CHA_NEW / RISKLIMITS; RUN;

PROC PHREG DATA=SHIM.DIS_1_CVA_S_7_N1; CLASS CASE; MODEL DIFFMO_2*CVA(0)=INSUP / RISKLIMITS; RUN;

TITLE '====================== IHD ========================';

PROC PHREG DATA=SHIM.DIS_1_IHD_S_7_N1; CLASS CASE; MODEL DIFFMO_2*IHD(0)=CASE / RISKLIMITS; RUN;

PROC PHREG DATA=SHIM.DIS_1_IHD_S_7_N1; CLASS CASE; MODEL DIFFMO_2*IHD(0)=TTX_CHOL / RISKLIMITS; RUN;

PROC PHREG DATA=SHIM.DIS_1_IHD_S_7_N1; CLASS CASE; MODEL DIFFMO_2*IHD(0)=TTX_COA / RISKLIMITS; RUN;

PROC PHREG DATA=SHIM.DIS_1_IHD_S_7_N1; CLASS CASE; MODEL DIFFMO_2*IHD(0)=TTX_PLT / RISKLIMITS; RUN;

PROC PHREG DATA=SHIM.DIS_1_IHD_S_7_N1; CLASS CASE; MODEL DIFFMO_2*IHD(0)=TTX_ANT / RISKLIMITS; RUN;

PROC PHREG DATA=SHIM.DIS_1_IHD_S_7_N1; CLASS CASE; MODEL DIFFMO_2*IHD(0)=DDX_HTN / RISKLIMITS; RUN;

PROC PHREG DATA=SHIM.DIS_1_IHD_S_7_N1; CLASS CASE; MODEL DIFFMO_2*IHD(0)=DDX_LIV / RISKLIMITS; RUN;

PROC PHREG DATA=SHIM.DIS_1_IHD_S_7_N1; CLASS CASE; MODEL DIFFMO_2*IHD(0)=DDX_CKD / RISKLIMITS; RUN;

PROC PHREG DATA=SHIM.DIS_1_IHD_S_7_N1; CLASS CASE; MODEL DIFFMO_2*IHD(0)=DDX_COPD / RISKLIMITS; RUN;

PROC PHREG DATA=SHIM.DIS_1_IHD_S_7_N1; CLASS CASE; MODEL DIFFMO_2*IHD(0)=DDX_ASTH / RISKLIMITS; RUN;

PROC PHREG DATA=SHIM.DIS_1_IHD_S_7_N1; CLASS CASE; MODEL DIFFMO_2*IHD(0)=DDX_CAN / RISKLIMITS; RUN;

PROC PHREG DATA=SHIM.DIS_1_IHD_S_7_N1; CLASS CASE; MODEL DIFFMO_2*IHD(0)=CITY / RISKLIMITS; RUN;

PROC PHREG DATA=SHIM.DIS_1_IHD_S_7_N1; CLASS CASE CHA_NEW(REF='1'); MODEL DIFFMO_2*IHD(0)=CHA_NEW / RISKLIMITS; RUN;

PROC PHREG DATA=SHIM.DIS_1_IHD_S_7_N1; CLASS CASE; MODEL DIFFMO_2*IHD(0)=INSUP / RISKLIMITS; RUN;

TITLE '====================== DM ========================';

PROC PHREG DATA=SHIM.DIS_1_DM_S_7_N1; CLASS CASE; MODEL DIFFMO_2*DM(0)=CASE / RISKLIMITS; RUN;

PROC PHREG DATA=SHIM.DIS_1_DM_S_7_N1; CLASS CASE; MODEL DIFFMO_2*DM(0)=TTX_CHOL / RISKLIMITS; RUN;

PROC PHREG DATA=SHIM.DIS_1_DM_S_7_N1; CLASS CASE; MODEL DIFFMO_2*DM(0)=TTX_COA / RISKLIMITS; RUN;

PROC PHREG DATA=SHIM.DIS_1_DM_S_7_N1; CLASS CASE; MODEL DIFFMO_2*DM(0)=TTX_PLT / RISKLIMITS; RUN;

PROC PHREG DATA=SHIM.DIS_1_DM_S_7_N1; CLASS CASE; MODEL DIFFMO_2*DM(0)=TTX_ANT / RISKLIMITS; RUN;

PROC PHREG DATA=SHIM.DIS_1_DM_S_7_N1; CLASS CASE; MODEL DIFFMO_2*DM(0)=DDX_HTN / RISKLIMITS; RUN;

PROC PHREG DATA=SHIM.DIS_1_DM_S_7_N1; CLASS CASE; MODEL DIFFMO_2*DM(0)=DDX_LIV / RISKLIMITS; RUN;

PROC PHREG DATA=SHIM.DIS_1_DM_S_7_N1; CLASS CASE; MODEL DIFFMO_2*DM(0)=DDX_CKD / RISKLIMITS; RUN;

PROC PHREG DATA=SHIM.DIS_1_DM_S_7_N1; CLASS CASE; MODEL DIFFMO_2*DM(0)=DDX_COPD / RISKLIMITS; RUN;

PROC PHREG DATA=SHIM.DIS_1_DM_S_7_N1; CLASS CASE; MODEL DIFFMO_2*DM(0)=DDX_ASTH / RISKLIMITS; RUN;

PROC PHREG DATA=SHIM.DIS_1_DM_S_7_N1; CLASS CASE; MODEL DIFFMO_2*DM(0)=DDX_CAN / RISKLIMITS; RUN;

PROC PHREG DATA=SHIM.DIS_1_DM_S_7_N1; CLASS CASE; MODEL DIFFMO_2*DM(0)=CITY / RISKLIMITS; RUN;

PROC PHREG DATA=SHIM.DIS_1_DM_S_7_N1; CLASS CASE CHA_NEW(REF='1'); MODEL DIFFMO_2*DM(0)=CHA_NEW / RISKLIMITS; RUN;

PROC PHREG DATA=SHIM.DIS_1_DM_S_7_N1; CLASS CASE; MODEL DIFFMO_2*DM(0)=INSUP / RISKLIMITS; RUN;

TITLE '====================== MI ========================';

PROC PHREG DATA=SHIM.DIS_1_MI_S_7_N1; CLASS CASE; MODEL DIFFMO_2*MI(0)=CASE / RISKLIMITS; RUN;

PROC PHREG DATA=SHIM.DIS_1_MI_S_7_N1; CLASS CASE; MODEL DIFFMO_2*MI(0)=TTX_CHOL / RISKLIMITS; RUN;

PROC PHREG DATA=SHIM.DIS_1_MI_S_7_N1; CLASS CASE; MODEL DIFFMO_2*MI(0)=TTX_COA / RISKLIMITS; RUN;

PROC PHREG DATA=SHIM.DIS_1_MI_S_7_N1; CLASS CASE; MODEL DIFFMO_2*MI(0)=TTX_PLT / RISKLIMITS; RUN;

PROC PHREG DATA=SHIM.DIS_1_MI_S_7_N1; CLASS CASE; MODEL DIFFMO_2*MI(0)=TTX_ANT / RISKLIMITS; RUN;

PROC PHREG DATA=SHIM.DIS_1_MI_S_7_N1; CLASS CASE; MODEL DIFFMO_2*MI(0)=DDX_HTN / RISKLIMITS; RUN;

PROC PHREG DATA=SHIM.DIS_1_MI_S_7_N1; CLASS CASE; MODEL DIFFMO_2*MI(0)=DDX_LIV / RISKLIMITS; RUN;

PROC PHREG DATA=SHIM.DIS_1_MI_S_7_N1; CLASS CASE; MODEL DIFFMO_2*MI(0)=DDX_CKD / RISKLIMITS; RUN;

PROC PHREG DATA=SHIM.DIS_1_MI_S_7_N1; CLASS CASE; MODEL DIFFMO_2*MI(0)=DDX_COPD / RISKLIMITS; RUN;

PROC PHREG DATA=SHIM.DIS_1_MI_S_7_N1; CLASS CASE; MODEL DIFFMO_2*MI(0)=DDX_ASTH / RISKLIMITS; RUN;

PROC PHREG DATA=SHIM.DIS_1_MI_S_7_N1; CLASS CASE; MODEL DIFFMO_2*MI(0)=DDX_CAN / RISKLIMITS; RUN;

PROC PHREG DATA=SHIM.DIS_1_MI_S_7_N1; CLASS CASE; MODEL DIFFMO_2*MI(0)=CITY / RISKLIMITS; RUN;

PROC PHREG DATA=SHIM.DIS_1_MI_S_7_N1; CLASS CASE CHA_NEW(REF='1'); MODEL DIFFMO_2*MI(0)=CHA_NEW / RISKLIMITS; RUN;

PROC PHREG DATA=SHIM.DIS_1_MI_S_7_N1; CLASS CASE; MODEL DIFFMO_2*MI(0)=INSUP / RISKLIMITS; RUN;

TITLE '====================== ALZ ========================';

PROC PHREG DATA=SHIM.DIS_2_ALZ_S_7_N1; CLASS CASE; MODEL DIFFMO_2*ALZ(0)=CASE / RISKLIMITS; RUN;

PROC PHREG DATA=SHIM.DIS_2_ALZ_S_7_N1; CLASS CASE; MODEL DIFFMO_2*ALZ(0)=TTX_CHOL / RISKLIMITS; RUN;

PROC PHREG DATA=SHIM.DIS_2_ALZ_S_7_N1; CLASS CASE; MODEL DIFFMO_2*ALZ(0)=TTX_COA / RISKLIMITS; RUN;

PROC PHREG DATA=SHIM.DIS_2_ALZ_S_7_N1; CLASS CASE; MODEL DIFFMO_2*ALZ(0)=TTX_PLT / RISKLIMITS; RUN;

PROC PHREG DATA=SHIM.DIS_2_ALZ_S_7_N1; CLASS CASE; MODEL DIFFMO_2*ALZ(0)=TTX_ANT / RISKLIMITS; RUN;

PROC PHREG DATA=SHIM.DIS_2_ALZ_S_7_N1; CLASS CASE; MODEL DIFFMO_2*ALZ(0)=DDX_HTN / RISKLIMITS; RUN;

PROC PHREG DATA=SHIM.DIS_2_ALZ_S_7_N1; CLASS CASE; MODEL DIFFMO_2*ALZ(0)=DDX_DM / RISKLIMITS; RUN;

PROC PHREG DATA=SHIM.DIS_2_ALZ_S_7_N1; CLASS CASE; MODEL DIFFMO_2*ALZ(0)=CVD / RISKLIMITS; RUN;

PROC PHREG DATA=SHIM.DIS_2_ALZ_S_7_N1; CLASS CASE; MODEL DIFFMO_2*ALZ(0)=DDX_LIV / RISKLIMITS; RUN;

PROC PHREG DATA=SHIM.DIS_2_ALZ_S_7_N1; CLASS CASE; MODEL DIFFMO_2*ALZ(0)=DDX_CKD / RISKLIMITS; RUN;

PROC PHREG DATA=SHIM.DIS_2_ALZ_S_7_N1; CLASS CASE; MODEL DIFFMO_2*ALZ(0)=DDX_COPD / RISKLIMITS; RUN;

PROC PHREG DATA=SHIM.DIS_2_ALZ_S_7_N1; CLASS CASE; MODEL DIFFMO_2*ALZ(0)=DDX_ASTH / RISKLIMITS; RUN;

PROC PHREG DATA=SHIM.DIS_2_ALZ_S_7_N1; CLASS CASE; MODEL DIFFMO_2*ALZ(0)=DDX_CAN / RISKLIMITS; RUN;

PROC PHREG DATA=SHIM.DIS_2_ALZ_S_7_N1; CLASS CASE; MODEL DIFFMO_2*ALZ(0)=CITY / RISKLIMITS; RUN;

PROC PHREG DATA=SHIM.DIS_2_ALZ_S_7_N1; CLASS CASE CHA_NEW(REF='1'); MODEL DIFFMO_2*ALZ(0)=CHA_NEW / RISKLIMITS; RUN;

PROC PHREG DATA=SHIM.DIS_2_ALZ_S_7_N1; CLASS CASE; MODEL DIFFMO_2*ALZ(0)=INSUP / RISKLIMITS; RUN;

TITLE '====================== PKS ========================';

PROC PHREG DATA=SHIM.DIS_2_PKS_S_7_N1; CLASS CASE; MODEL DIFFMO_2*PKS(0)=CASE / RISKLIMITS; RUN;

PROC PHREG DATA=SHIM.DIS_2_PKS_S_7_N1; CLASS CASE; MODEL DIFFMO_2*PKS(0)=TTX_CHOL / RISKLIMITS; RUN;

PROC PHREG DATA=SHIM.DIS_2_PKS_S_7_N1; CLASS CASE; MODEL DIFFMO_2*PKS(0)=TTX_COA / RISKLIMITS; RUN;

PROC PHREG DATA=SHIM.DIS_2_PKS_S_7_N1; CLASS CASE; MODEL DIFFMO_2*PKS(0)=TTX_PLT / RISKLIMITS; RUN;

PROC PHREG DATA=SHIM.DIS_2_PKS_S_7_N1; CLASS CASE; MODEL DIFFMO_2*PKS(0)=TTX_ANT / RISKLIMITS; RUN;

PROC PHREG DATA=SHIM.DIS_2_PKS_S_7_N1; CLASS CASE; MODEL DIFFMO_2*PKS(0)=DDX_HTN / RISKLIMITS; RUN;

PROC PHREG DATA=SHIM.DIS_2_PKS_S_7_N1; CLASS CASE; MODEL DIFFMO_2*PKS(0)=DDX_DM / RISKLIMITS; RUN;

PROC PHREG DATA=SHIM.DIS_2_PKS_S_7_N1; CLASS CASE; MODEL DIFFMO_2*PKS(0)=CVD / RISKLIMITS; RUN;

PROC PHREG DATA=SHIM.DIS_2_PKS_S_7_N1; CLASS CASE; MODEL DIFFMO_2*PKS(0)=DDX_LIV / RISKLIMITS; RUN;

PROC PHREG DATA=SHIM.DIS_2_PKS_S_7_N1; CLASS CASE; MODEL DIFFMO_2*PKS(0)=DDX_CKD / RISKLIMITS; RUN;

PROC PHREG DATA=SHIM.DIS_2_PKS_S_7_N1; CLASS CASE; MODEL DIFFMO_2*PKS(0)=DDX_COPD / RISKLIMITS; RUN;

PROC PHREG DATA=SHIM.DIS_2_PKS_S_7_N1; CLASS CASE; MODEL DIFFMO_2*PKS(0)=DDX_ASTH / RISKLIMITS; RUN;

PROC PHREG DATA=SHIM.DIS_2_PKS_S_7_N1; CLASS CASE; MODEL DIFFMO_2*PKS(0)=DDX_CAN / RISKLIMITS; RUN;

PROC PHREG DATA=SHIM.DIS_2_PKS_S_7_N1; CLASS CASE; MODEL DIFFMO_2*PKS(0)=CITY / RISKLIMITS; RUN;

PROC PHREG DATA=SHIM.DIS_2_PKS_S_7_N1; CLASS CASE CHA_NEW(REF='1'); MODEL DIFFMO_2*PKS(0)=CHA_NEW / RISKLIMITS; RUN;

PROC PHREG DATA=SHIM.DIS_2_PKS_S_7_N1; CLASS CASE; MODEL DIFFMO_2*PKS(0)=INSUP / RISKLIMITS; RUN;

TITLE '*****************************NAGE = 2***************************';

TITLE '====================== CVA ========================';

PROC PHREG DATA=SHIM.DIS_1_CVA_S_7_N2; CLASS CASE; MODEL DIFFMO_2*CVA(0)=CASE / RISKLIMITS; RUN;

PROC PHREG DATA=SHIM.DIS_1_CVA_S_7_N2; CLASS CASE; MODEL DIFFMO_2*CVA(0)=TTX_CHOL / RISKLIMITS; RUN;

PROC PHREG DATA=SHIM.DIS_1_CVA_S_7_N2; CLASS CASE; MODEL DIFFMO_2*CVA(0)=TTX_COA / RISKLIMITS; RUN;

PROC PHREG DATA=SHIM.DIS_1_CVA_S_7_N2; CLASS CASE; MODEL DIFFMO_2*CVA(0)=TTX_PLT / RISKLIMITS; RUN;

PROC PHREG DATA=SHIM.DIS_1_CVA_S_7_N2; CLASS CASE; MODEL DIFFMO_2*CVA(0)=TTX_ANT / RISKLIMITS; RUN;

PROC PHREG DATA=SHIM.DIS_1_CVA_S_7_N2; CLASS CASE; MODEL DIFFMO_2*CVA(0)=DDX_HTN / RISKLIMITS; RUN;

PROC PHREG DATA=SHIM.DIS_1_CVA_S_7_N2; CLASS CASE; MODEL DIFFMO_2*CVA(0)=DDX_LIV / RISKLIMITS; RUN;

PROC PHREG DATA=SHIM.DIS_1_CVA_S_7_N2; CLASS CASE; MODEL DIFFMO_2*CVA(0)=DDX_CKD / RISKLIMITS; RUN;

PROC PHREG DATA=SHIM.DIS_1_CVA_S_7_N2; CLASS CASE; MODEL DIFFMO_2*CVA(0)=DDX_COPD / RISKLIMITS; RUN;

PROC PHREG DATA=SHIM.DIS_1_CVA_S_7_N2; CLASS CASE; MODEL DIFFMO_2*CVA(0)=DDX_ASTH / RISKLIMITS; RUN;

PROC PHREG DATA=SHIM.DIS_1_CVA_S_7_N2; CLASS CASE; MODEL DIFFMO_2*CVA(0)=DDX_CAN / RISKLIMITS; RUN;

PROC PHREG DATA=SHIM.DIS_1_CVA_S_7_N2; CLASS CASE; MODEL DIFFMO_2*CVA(0)=CITY / RISKLIMITS; RUN;

PROC PHREG DATA=SHIM.DIS_1_CVA_S_7_N2; CLASS CASE CHA_NEW(REF='1'); MODEL DIFFMO_2*CVA(0)=CHA_NEW / RISKLIMITS; RUN;

PROC PHREG DATA=SHIM.DIS_1_CVA_S_7_N2; CLASS CASE; MODEL DIFFMO_2*CVA(0)=INSUP / RISKLIMITS; RUN;

TITLE '====================== IHD ========================';

PROC PHREG DATA=SHIM.DIS_1_IHD_S_7_N2; CLASS CASE; MODEL DIFFMO_2*IHD(0)=CASE / RISKLIMITS; RUN;

PROC PHREG DATA=SHIM.DIS_1_IHD_S_7_N2; CLASS CASE; MODEL DIFFMO_2*IHD(0)=TTX_CHOL / RISKLIMITS; RUN;

PROC PHREG DATA=SHIM.DIS_1_IHD_S_7_N2; CLASS CASE; MODEL DIFFMO_2*IHD(0)=TTX_COA / RISKLIMITS; RUN;

PROC PHREG DATA=SHIM.DIS_1_IHD_S_7_N2; CLASS CASE; MODEL DIFFMO_2*IHD(0)=TTX_PLT / RISKLIMITS; RUN;

PROC PHREG DATA=SHIM.DIS_1_IHD_S_7_N2; CLASS CASE; MODEL DIFFMO_2*IHD(0)=TTX_ANT / RISKLIMITS; RUN;

PROC PHREG DATA=SHIM.DIS_1_IHD_S_7_N2; CLASS CASE; MODEL DIFFMO_2*IHD(0)=DDX_HTN / RISKLIMITS; RUN;

PROC PHREG DATA=SHIM.DIS_1_IHD_S_7_N2; CLASS CASE; MODEL DIFFMO_2*IHD(0)=DDX_LIV / RISKLIMITS; RUN;

PROC PHREG DATA=SHIM.DIS_1_IHD_S_7_N2; CLASS CASE; MODEL DIFFMO_2*IHD(0)=DDX_CKD / RISKLIMITS; RUN;

PROC PHREG DATA=SHIM.DIS_1_IHD_S_7_N2; CLASS CASE; MODEL DIFFMO_2*IHD(0)=DDX_COPD / RISKLIMITS; RUN;

PROC PHREG DATA=SHIM.DIS_1_IHD_S_7_N2; CLASS CASE; MODEL DIFFMO_2*IHD(0)=DDX_ASTH / RISKLIMITS; RUN;

PROC PHREG DATA=SHIM.DIS_1_IHD_S_7_N2; CLASS CASE; MODEL DIFFMO_2*IHD(0)=DDX_CAN / RISKLIMITS; RUN;

PROC PHREG DATA=SHIM.DIS_1_IHD_S_7_N2; CLASS CASE; MODEL DIFFMO_2*IHD(0)=CITY / RISKLIMITS; RUN;

PROC PHREG DATA=SHIM.DIS_1_IHD_S_7_N2; CLASS CASE CHA_NEW(REF='1'); MODEL DIFFMO_2*IHD(0)=CHA_NEW / RISKLIMITS; RUN;

PROC PHREG DATA=SHIM.DIS_1_IHD_S_7_N2; CLASS CASE; MODEL DIFFMO_2*IHD(0)=INSUP / RISKLIMITS; RUN;

TITLE '====================== DM ========================';

PROC PHREG DATA=SHIM.DIS_1_DM_S_7_N2; CLASS CASE; MODEL DIFFMO_2*DM(0)=CASE / RISKLIMITS; RUN;

PROC PHREG DATA=SHIM.DIS_1_DM_S_7_N2; CLASS CASE; MODEL DIFFMO_2*DM(0)=TTX_CHOL / RISKLIMITS; RUN;

PROC PHREG DATA=SHIM.DIS_1_DM_S_7_N2; CLASS CASE; MODEL DIFFMO_2*DM(0)=TTX_COA / RISKLIMITS; RUN;

PROC PHREG DATA=SHIM.DIS_1_DM_S_7_N2; CLASS CASE; MODEL DIFFMO_2*DM(0)=TTX_PLT / RISKLIMITS; RUN;

PROC PHREG DATA=SHIM.DIS_1_DM_S_7_N2; CLASS CASE; MODEL DIFFMO_2*DM(0)=TTX_ANT / RISKLIMITS; RUN;

PROC PHREG DATA=SHIM.DIS_1_DM_S_7_N2; CLASS CASE; MODEL DIFFMO_2*DM(0)=DDX_HTN / RISKLIMITS; RUN;

PROC PHREG DATA=SHIM.DIS_1_DM_S_7_N2; CLASS CASE; MODEL DIFFMO_2*DM(0)=DDX_LIV / RISKLIMITS; RUN;

PROC PHREG DATA=SHIM.DIS_1_DM_S_7_N2; CLASS CASE; MODEL DIFFMO_2*DM(0)=DDX_CKD / RISKLIMITS; RUN;

PROC PHREG DATA=SHIM.DIS_1_DM_S_7_N2; CLASS CASE; MODEL DIFFMO_2*DM(0)=DDX_COPD / RISKLIMITS; RUN;

PROC PHREG DATA=SHIM.DIS_1_DM_S_7_N2; CLASS CASE; MODEL DIFFMO_2*DM(0)=DDX_ASTH / RISKLIMITS; RUN;

PROC PHREG DATA=SHIM.DIS_1_DM_S_7_N2; CLASS CASE; MODEL DIFFMO_2*DM(0)=DDX_CAN / RISKLIMITS; RUN;

PROC PHREG DATA=SHIM.DIS_1_DM_S_7_N2; CLASS CASE; MODEL DIFFMO_2*DM(0)=CITY / RISKLIMITS; RUN;

PROC PHREG DATA=SHIM.DIS_1_DM_S_7_N2; CLASS CASE CHA_NEW(REF='1'); MODEL DIFFMO_2*DM(0)=CHA_NEW / RISKLIMITS; RUN;

PROC PHREG DATA=SHIM.DIS_1_DM_S_7_N2; CLASS CASE; MODEL DIFFMO_2*DM(0)=INSUP / RISKLIMITS; RUN;

TITLE '====================== MI ========================';

PROC PHREG DATA=SHIM.DIS_1_MI_S_7_N2; CLASS CASE; MODEL DIFFMO_2*MI(0)=CASE / RISKLIMITS; RUN;

PROC PHREG DATA=SHIM.DIS_1_MI_S_7_N2; CLASS CASE; MODEL DIFFMO_2*MI(0)=TTX_CHOL / RISKLIMITS; RUN;

PROC PHREG DATA=SHIM.DIS_1_MI_S_7_N2; CLASS CASE; MODEL DIFFMO_2*MI(0)=TTX_COA / RISKLIMITS; RUN;

PROC PHREG DATA=SHIM.DIS_1_MI_S_7_N2; CLASS CASE; MODEL DIFFMO_2*MI(0)=TTX_PLT / RISKLIMITS; RUN;

PROC PHREG DATA=SHIM.DIS_1_MI_S_7_N2; CLASS CASE; MODEL DIFFMO_2*MI(0)=TTX_ANT / RISKLIMITS; RUN;

PROC PHREG DATA=SHIM.DIS_1_MI_S_7_N2; CLASS CASE; MODEL DIFFMO_2*MI(0)=DDX_HTN / RISKLIMITS; RUN;

PROC PHREG DATA=SHIM.DIS_1_MI_S_7_N2; CLASS CASE; MODEL DIFFMO_2*MI(0)=DDX_LIV / RISKLIMITS; RUN;

PROC PHREG DATA=SHIM.DIS_1_MI_S_7_N2; CLASS CASE; MODEL DIFFMO_2*MI(0)=DDX_CKD / RISKLIMITS; RUN;

PROC PHREG DATA=SHIM.DIS_1_MI_S_7_N2; CLASS CASE; MODEL DIFFMO_2*MI(0)=DDX_COPD / RISKLIMITS; RUN;

PROC PHREG DATA=SHIM.DIS_1_MI_S_7_N2; CLASS CASE; MODEL DIFFMO_2*MI(0)=DDX_ASTH / RISKLIMITS; RUN;

PROC PHREG DATA=SHIM.DIS_1_MI_S_7_N2; CLASS CASE; MODEL DIFFMO_2*MI(0)=DDX_CAN / RISKLIMITS; RUN;

PROC PHREG DATA=SHIM.DIS_1_MI_S_7_N2; CLASS CASE; MODEL DIFFMO_2*MI(0)=CITY / RISKLIMITS; RUN;

PROC PHREG DATA=SHIM.DIS_1_MI_S_7_N2; CLASS CASE CHA_NEW(REF='1'); MODEL DIFFMO_2*MI(0)=CHA_NEW / RISKLIMITS; RUN;

PROC PHREG DATA=SHIM.DIS_1_MI_S_7_N2; CLASS CASE; MODEL DIFFMO_2*MI(0)=INSUP / RISKLIMITS; RUN;

TITLE '====================== ALZ ========================';

PROC PHREG DATA=SHIM.DIS_2_ALZ_S_7_N2; CLASS CASE; MODEL DIFFMO_2*ALZ(0)=CASE / RISKLIMITS; RUN;

PROC PHREG DATA=SHIM.DIS_2_ALZ_S_7_N2; CLASS CASE; MODEL DIFFMO_2*ALZ(0)=TTX_CHOL / RISKLIMITS; RUN;

PROC PHREG DATA=SHIM.DIS_2_ALZ_S_7_N2; CLASS CASE; MODEL DIFFMO_2*ALZ(0)=TTX_COA / RISKLIMITS; RUN;

PROC PHREG DATA=SHIM.DIS_2_ALZ_S_7_N2; CLASS CASE; MODEL DIFFMO_2*ALZ(0)=TTX_PLT / RISKLIMITS; RUN;

PROC PHREG DATA=SHIM.DIS_2_ALZ_S_7_N2; CLASS CASE; MODEL DIFFMO_2*ALZ(0)=TTX_ANT / RISKLIMITS; RUN;

PROC PHREG DATA=SHIM.DIS_2_ALZ_S_7_N2; CLASS CASE; MODEL DIFFMO_2*ALZ(0)=DDX_HTN / RISKLIMITS; RUN;

PROC PHREG DATA=SHIM.DIS_2_ALZ_S_7_N2; CLASS CASE; MODEL DIFFMO_2*ALZ(0)=DDX_DM / RISKLIMITS; RUN;

PROC PHREG DATA=SHIM.DIS_2_ALZ_S_7_N2; CLASS CASE; MODEL DIFFMO_2*ALZ(0)=CVD / RISKLIMITS; RUN;

PROC PHREG DATA=SHIM.DIS_2_ALZ_S_7_N2; CLASS CASE; MODEL DIFFMO_2*ALZ(0)=DDX_LIV / RISKLIMITS; RUN;

PROC PHREG DATA=SHIM.DIS_2_ALZ_S_7_N2; CLASS CASE; MODEL DIFFMO_2*ALZ(0)=DDX_CKD / RISKLIMITS; RUN;

PROC PHREG DATA=SHIM.DIS_2_ALZ_S_7_N2; CLASS CASE; MODEL DIFFMO_2*ALZ(0)=DDX_COPD / RISKLIMITS; RUN;

PROC PHREG DATA=SHIM.DIS_2_ALZ_S_7_N2; CLASS CASE; MODEL DIFFMO_2*ALZ(0)=DDX_ASTH / RISKLIMITS; RUN;

PROC PHREG DATA=SHIM.DIS_2_ALZ_S_7_N2; CLASS CASE; MODEL DIFFMO_2*ALZ(0)=DDX_CAN / RISKLIMITS; RUN;

PROC PHREG DATA=SHIM.DIS_2_ALZ_S_7_N2; CLASS CASE; MODEL DIFFMO_2*ALZ(0)=CITY / RISKLIMITS; RUN;

PROC PHREG DATA=SHIM.DIS_2_ALZ_S_7_N2; CLASS CASE CHA_NEW(REF='1'); MODEL DIFFMO_2*ALZ(0)=CHA_NEW / RISKLIMITS; RUN;

PROC PHREG DATA=SHIM.DIS_2_ALZ_S_7_N2; CLASS CASE; MODEL DIFFMO_2*ALZ(0)=INSUP / RISKLIMITS; RUN;

TITLE '====================== PKS ========================';

PROC PHREG DATA=SHIM.DIS_2_PKS_S_7_N2; CLASS CASE; MODEL DIFFMO_2*PKS(0)=CASE / RISKLIMITS; RUN;

PROC PHREG DATA=SHIM.DIS_2_PKS_S_7_N2; CLASS CASE; MODEL DIFFMO_2*PKS(0)=TTX_CHOL / RISKLIMITS; RUN;

PROC PHREG DATA=SHIM.DIS_2_PKS_S_7_N2; CLASS CASE; MODEL DIFFMO_2*PKS(0)=TTX_COA / RISKLIMITS; RUN;

PROC PHREG DATA=SHIM.DIS_2_PKS_S_7_N2; CLASS CASE; MODEL DIFFMO_2*PKS(0)=TTX_PLT / RISKLIMITS; RUN;

PROC PHREG DATA=SHIM.DIS_2_PKS_S_7_N2; CLASS CASE; MODEL DIFFMO_2*PKS(0)=TTX_ANT / RISKLIMITS; RUN;

PROC PHREG DATA=SHIM.DIS_2_PKS_S_7_N2; CLASS CASE; MODEL DIFFMO_2*PKS(0)=DDX_HTN / RISKLIMITS; RUN;

PROC PHREG DATA=SHIM.DIS_2_PKS_S_7_N2; CLASS CASE; MODEL DIFFMO_2*PKS(0)=DDX_DM / RISKLIMITS; RUN;

PROC PHREG DATA=SHIM.DIS_2_PKS_S_7_N2; CLASS CASE; MODEL DIFFMO_2*PKS(0)=CVD / RISKLIMITS; RUN;

PROC PHREG DATA=SHIM.DIS_2_PKS_S_7_N2; CLASS CASE; MODEL DIFFMO_2*PKS(0)=DDX_LIV / RISKLIMITS; RUN;

PROC PHREG DATA=SHIM.DIS_2_PKS_S_7_N2; CLASS CASE; MODEL DIFFMO_2*PKS(0)=DDX_CKD / RISKLIMITS; RUN;

PROC PHREG DATA=SHIM.DIS_2_PKS_S_7_N2; CLASS CASE; MODEL DIFFMO_2*PKS(0)=DDX_COPD / RISKLIMITS; RUN;

PROC PHREG DATA=SHIM.DIS_2_PKS_S_7_N2; CLASS CASE; MODEL DIFFMO_2*PKS(0)=DDX_ASTH / RISKLIMITS; RUN;

PROC PHREG DATA=SHIM.DIS_2_PKS_S_7_N2; CLASS CASE; MODEL DIFFMO_2*PKS(0)=DDX_CAN / RISKLIMITS; RUN;

PROC PHREG DATA=SHIM.DIS_2_PKS_S_7_N2; CLASS CASE; MODEL DIFFMO_2*PKS(0)=CITY / RISKLIMITS; RUN;

PROC PHREG DATA=SHIM.DIS_2_PKS_S_7_N2; CLASS CASE CHA_NEW(REF='1'); MODEL DIFFMO_2*PKS(0)=CHA_NEW / RISKLIMITS; RUN;

PROC PHREG DATA=SHIM.DIS_2_PKS_S_7_N2; CLASS CASE; MODEL DIFFMO_2*PKS(0)=INSUP / RISKLIMITS; RUN;

TITLE '*****************************NAGE = 3***************************';

TITLE '====================== CVA ========================';

PROC PHREG DATA=SHIM.DIS_1_CVA_S_7_N3; CLASS CASE; MODEL DIFFMO_2*CVA(0)=CASE / RISKLIMITS; RUN;

PROC PHREG DATA=SHIM.DIS_1_CVA_S_7_N3; CLASS CASE; MODEL DIFFMO_2*CVA(0)=TTX_CHOL / RISKLIMITS; RUN;

PROC PHREG DATA=SHIM.DIS_1_CVA_S_7_N3; CLASS CASE; MODEL DIFFMO_2*CVA(0)=TTX_COA / RISKLIMITS; RUN;

PROC PHREG DATA=SHIM.DIS_1_CVA_S_7_N3; CLASS CASE; MODEL DIFFMO_2*CVA(0)=TTX_PLT / RISKLIMITS; RUN;

PROC PHREG DATA=SHIM.DIS_1_CVA_S_7_N3; CLASS CASE; MODEL DIFFMO_2*CVA(0)=TTX_ANT / RISKLIMITS; RUN;

PROC PHREG DATA=SHIM.DIS_1_CVA_S_7_N3; CLASS CASE; MODEL DIFFMO_2*CVA(0)=DDX_HTN / RISKLIMITS; RUN;

PROC PHREG DATA=SHIM.DIS_1_CVA_S_7_N3; CLASS CASE; MODEL DIFFMO_2*CVA(0)=DDX_LIV / RISKLIMITS; RUN;

PROC PHREG DATA=SHIM.DIS_1_CVA_S_7_N3; CLASS CASE; MODEL DIFFMO_2*CVA(0)=DDX_CKD / RISKLIMITS; RUN;

PROC PHREG DATA=SHIM.DIS_1_CVA_S_7_N3; CLASS CASE; MODEL DIFFMO_2*CVA(0)=DDX_COPD / RISKLIMITS; RUN;

PROC PHREG DATA=SHIM.DIS_1_CVA_S_7_N3; CLASS CASE; MODEL DIFFMO_2*CVA(0)=DDX_ASTH / RISKLIMITS; RUN;

PROC PHREG DATA=SHIM.DIS_1_CVA_S_7_N3; CLASS CASE; MODEL DIFFMO_2*CVA(0)=DDX_CAN / RISKLIMITS; RUN;

PROC PHREG DATA=SHIM.DIS_1_CVA_S_7_N3; CLASS CASE; MODEL DIFFMO_2*CVA(0)=CITY / RISKLIMITS; RUN;

PROC PHREG DATA=SHIM.DIS_1_CVA_S_7_N3; CLASS CASE CHA_NEW(REF='1'); MODEL DIFFMO_2*CVA(0)=CHA_NEW / RISKLIMITS; RUN;

PROC PHREG DATA=SHIM.DIS_1_CVA_S_7_N3; CLASS CASE; MODEL DIFFMO_2*CVA(0)=INSUP / RISKLIMITS; RUN;

TITLE '====================== IHD ========================';

PROC PHREG DATA=SHIM.DIS_1_IHD_S_7_N3; CLASS CASE; MODEL DIFFMO_2*IHD(0)=CASE / RISKLIMITS; RUN;

PROC PHREG DATA=SHIM.DIS_1_IHD_S_7_N3; CLASS CASE; MODEL DIFFMO_2*IHD(0)=TTX_CHOL / RISKLIMITS; RUN;

PROC PHREG DATA=SHIM.DIS_1_IHD_S_7_N3; CLASS CASE; MODEL DIFFMO_2*IHD(0)=TTX_COA / RISKLIMITS; RUN;

PROC PHREG DATA=SHIM.DIS_1_IHD_S_7_N3; CLASS CASE; MODEL DIFFMO_2*IHD(0)=TTX_PLT / RISKLIMITS; RUN;

PROC PHREG DATA=SHIM.DIS_1_IHD_S_7_N3; CLASS CASE; MODEL DIFFMO_2*IHD(0)=TTX_ANT / RISKLIMITS; RUN;

PROC PHREG DATA=SHIM.DIS_1_IHD_S_7_N3; CLASS CASE; MODEL DIFFMO_2*IHD(0)=DDX_HTN / RISKLIMITS; RUN;

PROC PHREG DATA=SHIM.DIS_1_IHD_S_7_N3; CLASS CASE; MODEL DIFFMO_2*IHD(0)=DDX_LIV / RISKLIMITS; RUN;

PROC PHREG DATA=SHIM.DIS_1_IHD_S_7_N3; CLASS CASE; MODEL DIFFMO_2*IHD(0)=DDX_CKD / RISKLIMITS; RUN;

PROC PHREG DATA=SHIM.DIS_1_IHD_S_7_N3; CLASS CASE; MODEL DIFFMO_2*IHD(0)=DDX_COPD / RISKLIMITS; RUN;

PROC PHREG DATA=SHIM.DIS_1_IHD_S_7_N3; CLASS CASE; MODEL DIFFMO_2*IHD(0)=DDX_ASTH / RISKLIMITS; RUN;

PROC PHREG DATA=SHIM.DIS_1_IHD_S_7_N3; CLASS CASE; MODEL DIFFMO_2*IHD(0)=DDX_CAN / RISKLIMITS; RUN;

PROC PHREG DATA=SHIM.DIS_1_IHD_S_7_N3; CLASS CASE; MODEL DIFFMO_2*IHD(0)=CITY / RISKLIMITS; RUN;

PROC PHREG DATA=SHIM.DIS_1_IHD_S_7_N3; CLASS CASE CHA_NEW(REF='1'); MODEL DIFFMO_2*IHD(0)=CHA_NEW / RISKLIMITS; RUN;

PROC PHREG DATA=SHIM.DIS_1_IHD_S_7_N3; CLASS CASE; MODEL DIFFMO_2*IHD(0)=INSUP / RISKLIMITS; RUN;

TITLE '====================== DM ========================';

PROC PHREG DATA=SHIM.DIS_1_DM_S_7_N3; CLASS CASE; MODEL DIFFMO_2*DM(0)=CASE / RISKLIMITS; RUN;

PROC PHREG DATA=SHIM.DIS_1_DM_S_7_N3; CLASS CASE; MODEL DIFFMO_2*DM(0)=TTX_CHOL / RISKLIMITS; RUN;

PROC PHREG DATA=SHIM.DIS_1_DM_S_7_N3; CLASS CASE; MODEL DIFFMO_2*DM(0)=TTX_COA / RISKLIMITS; RUN;

PROC PHREG DATA=SHIM.DIS_1_DM_S_7_N3; CLASS CASE; MODEL DIFFMO_2*DM(0)=TTX_PLT / RISKLIMITS; RUN;

PROC PHREG DATA=SHIM.DIS_1_DM_S_7_N3; CLASS CASE; MODEL DIFFMO_2*DM(0)=TTX_ANT / RISKLIMITS; RUN;

PROC PHREG DATA=SHIM.DIS_1_DM_S_7_N3; CLASS CASE; MODEL DIFFMO_2*DM(0)=DDX_HTN / RISKLIMITS; RUN;

PROC PHREG DATA=SHIM.DIS_1_DM_S_7_N3; CLASS CASE; MODEL DIFFMO_2*DM(0)=DDX_LIV / RISKLIMITS; RUN;

PROC PHREG DATA=SHIM.DIS_1_DM_S_7_N3; CLASS CASE; MODEL DIFFMO_2*DM(0)=DDX_CKD / RISKLIMITS; RUN;

PROC PHREG DATA=SHIM.DIS_1_DM_S_7_N3; CLASS CASE; MODEL DIFFMO_2*DM(0)=DDX_COPD / RISKLIMITS; RUN;

PROC PHREG DATA=SHIM.DIS_1_DM_S_7_N3; CLASS CASE; MODEL DIFFMO_2*DM(0)=DDX_ASTH / RISKLIMITS; RUN;

PROC PHREG DATA=SHIM.DIS_1_DM_S_7_N3; CLASS CASE; MODEL DIFFMO_2*DM(0)=DDX_CAN / RISKLIMITS; RUN;

PROC PHREG DATA=SHIM.DIS_1_DM_S_7_N3; CLASS CASE; MODEL DIFFMO_2*DM(0)=CITY / RISKLIMITS; RUN;

PROC PHREG DATA=SHIM.DIS_1_DM_S_7_N3; CLASS CASE CHA_NEW(REF='1'); MODEL DIFFMO_2*DM(0)=CHA_NEW / RISKLIMITS; RUN;

PROC PHREG DATA=SHIM.DIS_1_DM_S_7_N3; CLASS CASE; MODEL DIFFMO_2*DM(0)=INSUP / RISKLIMITS; RUN;

TITLE '====================== MI ========================';

PROC PHREG DATA=SHIM.DIS_1_MI_S_7_N3; CLASS CASE; MODEL DIFFMO_2*MI(0)=CASE / RISKLIMITS; RUN;

PROC PHREG DATA=SHIM.DIS_1_MI_S_7_N3; CLASS CASE; MODEL DIFFMO_2*MI(0)=TTX_CHOL / RISKLIMITS; RUN;

PROC PHREG DATA=SHIM.DIS_1_MI_S_7_N3; CLASS CASE; MODEL DIFFMO_2*MI(0)=TTX_COA / RISKLIMITS; RUN;

PROC PHREG DATA=SHIM.DIS_1_MI_S_7_N3; CLASS CASE; MODEL DIFFMO_2*MI(0)=TTX_PLT / RISKLIMITS; RUN;

PROC PHREG DATA=SHIM.DIS_1_MI_S_7_N3; CLASS CASE; MODEL DIFFMO_2*MI(0)=TTX_ANT / RISKLIMITS; RUN;

PROC PHREG DATA=SHIM.DIS_1_MI_S_7_N3; CLASS CASE; MODEL DIFFMO_2*MI(0)=DDX_HTN / RISKLIMITS; RUN;

PROC PHREG DATA=SHIM.DIS_1_MI_S_7_N3; CLASS CASE; MODEL DIFFMO_2*MI(0)=DDX_LIV / RISKLIMITS; RUN;

PROC PHREG DATA=SHIM.DIS_1_MI_S_7_N3; CLASS CASE; MODEL DIFFMO_2*MI(0)=DDX_CKD / RISKLIMITS; RUN;

PROC PHREG DATA=SHIM.DIS_1_MI_S_7_N3; CLASS CASE; MODEL DIFFMO_2*MI(0)=DDX_COPD / RISKLIMITS; RUN;

PROC PHREG DATA=SHIM.DIS_1_MI_S_7_N3; CLASS CASE; MODEL DIFFMO_2*MI(0)=DDX_ASTH / RISKLIMITS; RUN;

PROC PHREG DATA=SHIM.DIS_1_MI_S_7_N3; CLASS CASE; MODEL DIFFMO_2*MI(0)=DDX_CAN / RISKLIMITS; RUN;

PROC PHREG DATA=SHIM.DIS_1_MI_S_7_N3; CLASS CASE; MODEL DIFFMO_2*MI(0)=CITY / RISKLIMITS; RUN;

PROC PHREG DATA=SHIM.DIS_1_MI_S_7_N3; CLASS CASE CHA_NEW(REF='1'); MODEL DIFFMO_2*MI(0)=CHA_NEW / RISKLIMITS; RUN;

PROC PHREG DATA=SHIM.DIS_1_MI_S_7_N3; CLASS CASE; MODEL DIFFMO_2*MI(0)=INSUP / RISKLIMITS; RUN;

TITLE '====================== ALZ ========================';

PROC PHREG DATA=SHIM.DIS_2_ALZ_S_7_N3; CLASS CASE; MODEL DIFFMO_2*ALZ(0)=CASE / RISKLIMITS; RUN;

PROC PHREG DATA=SHIM.DIS_2_ALZ_S_7_N3; CLASS CASE; MODEL DIFFMO_2*ALZ(0)=TTX_CHOL / RISKLIMITS; RUN;

PROC PHREG DATA=SHIM.DIS_2_ALZ_S_7_N3; CLASS CASE; MODEL DIFFMO_2*ALZ(0)=TTX_COA / RISKLIMITS; RUN;

PROC PHREG DATA=SHIM.DIS_2_ALZ_S_7_N3; CLASS CASE; MODEL DIFFMO_2*ALZ(0)=TTX_PLT / RISKLIMITS; RUN;

PROC PHREG DATA=SHIM.DIS_2_ALZ_S_7_N3; CLASS CASE; MODEL DIFFMO_2*ALZ(0)=TTX_ANT / RISKLIMITS; RUN;

PROC PHREG DATA=SHIM.DIS_2_ALZ_S_7_N3; CLASS CASE; MODEL DIFFMO_2*ALZ(0)=DDX_HTN / RISKLIMITS; RUN;

PROC PHREG DATA=SHIM.DIS_2_ALZ_S_7_N3; CLASS CASE; MODEL DIFFMO_2*ALZ(0)=DDX_DM / RISKLIMITS; RUN;

PROC PHREG DATA=SHIM.DIS_2_ALZ_S_7_N3; CLASS CASE; MODEL DIFFMO_2*ALZ(0)=CVD / RISKLIMITS; RUN;

PROC PHREG DATA=SHIM.DIS_2_ALZ_S_7_N3; CLASS CASE; MODEL DIFFMO_2*ALZ(0)=DDX_LIV / RISKLIMITS; RUN;

PROC PHREG DATA=SHIM.DIS_2_ALZ_S_7_N3; CLASS CASE; MODEL DIFFMO_2*ALZ(0)=DDX_CKD / RISKLIMITS; RUN;

PROC PHREG DATA=SHIM.DIS_2_ALZ_S_7_N3; CLASS CASE; MODEL DIFFMO_2*ALZ(0)=DDX_COPD / RISKLIMITS; RUN;

PROC PHREG DATA=SHIM.DIS_2_ALZ_S_7_N3; CLASS CASE; MODEL DIFFMO_2*ALZ(0)=DDX_ASTH / RISKLIMITS; RUN;

PROC PHREG DATA=SHIM.DIS_2_ALZ_S_7_N3; CLASS CASE; MODEL DIFFMO_2*ALZ(0)=DDX_CAN / RISKLIMITS; RUN;

PROC PHREG DATA=SHIM.DIS_2_ALZ_S_7_N3; CLASS CASE; MODEL DIFFMO_2*ALZ(0)=CITY / RISKLIMITS; RUN;

PROC PHREG DATA=SHIM.DIS_2_ALZ_S_7_N3; CLASS CASE CHA_NEW(REF='1'); MODEL DIFFMO_2*ALZ(0)=CHA_NEW / RISKLIMITS; RUN;

PROC PHREG DATA=SHIM.DIS_2_ALZ_S_7_N3; CLASS CASE; MODEL DIFFMO_2*ALZ(0)=INSUP / RISKLIMITS; RUN;

TITLE '====================== PKS ========================';

PROC PHREG DATA=SHIM.DIS_2_PKS_S_7_N3; CLASS CASE; MODEL DIFFMO_2*PKS(0)=CASE / RISKLIMITS; RUN;

PROC PHREG DATA=SHIM.DIS_2_PKS_S_7_N3; CLASS CASE; MODEL DIFFMO_2*PKS(0)=TTX_CHOL / RISKLIMITS; RUN;

PROC PHREG DATA=SHIM.DIS_2_PKS_S_7_N3; CLASS CASE; MODEL DIFFMO_2*PKS(0)=TTX_COA / RISKLIMITS; RUN;

PROC PHREG DATA=SHIM.DIS_2_PKS_S_7_N3; CLASS CASE; MODEL DIFFMO_2*PKS(0)=TTX_PLT / RISKLIMITS; RUN;

PROC PHREG DATA=SHIM.DIS_2_PKS_S_7_N3; CLASS CASE; MODEL DIFFMO_2*PKS(0)=TTX_ANT / RISKLIMITS; RUN;

PROC PHREG DATA=SHIM.DIS_2_PKS_S_7_N3; CLASS CASE; MODEL DIFFMO_2*PKS(0)=DDX_HTN / RISKLIMITS; RUN;

PROC PHREG DATA=SHIM.DIS_2_PKS_S_7_N3; CLASS CASE; MODEL DIFFMO_2*PKS(0)=DDX_DM / RISKLIMITS; RUN;

PROC PHREG DATA=SHIM.DIS_2_PKS_S_7_N3; CLASS CASE; MODEL DIFFMO_2*PKS(0)=CVD / RISKLIMITS; RUN;

PROC PHREG DATA=SHIM.DIS_2_PKS_S_7_N3; CLASS CASE; MODEL DIFFMO_2*PKS(0)=DDX_LIV / RISKLIMITS; RUN;

PROC PHREG DATA=SHIM.DIS_2_PKS_S_7_N3; CLASS CASE; MODEL DIFFMO_2*PKS(0)=DDX_CKD / RISKLIMITS; RUN;

PROC PHREG DATA=SHIM.DIS_2_PKS_S_7_N3; CLASS CASE; MODEL DIFFMO_2*PKS(0)=DDX_COPD / RISKLIMITS; RUN;

PROC PHREG DATA=SHIM.DIS_2_PKS_S_7_N3; CLASS CASE; MODEL DIFFMO_2*PKS(0)=DDX_ASTH / RISKLIMITS; RUN;

PROC PHREG DATA=SHIM.DIS_2_PKS_S_7_N3; CLASS CASE; MODEL DIFFMO_2*PKS(0)=DDX_CAN / RISKLIMITS; RUN;

PROC PHREG DATA=SHIM.DIS_2_PKS_S_7_N3; CLASS CASE; MODEL DIFFMO_2*PKS(0)=CITY / RISKLIMITS; RUN;

PROC PHREG DATA=SHIM.DIS_2_PKS_S_7_N3; CLASS CASE CHA_NEW(REF='1'); MODEL DIFFMO_2*PKS(0)=CHA_NEW / RISKLIMITS; RUN;

PROC PHREG DATA=SHIM.DIS_2_PKS_S_7_N3; CLASS CASE; MODEL DIFFMO_2*PKS(0)=INSUP / RISKLIMITS; RUN;

TITLE '*****************************BAGE = 0 ***************************';

TITLE '====================== CVA ========================';

PROC PHREG DATA=SHIM.DIS_1_CVA_S_7_B0; CLASS CASE; MODEL DIFFMO_2*CVA(0)=CASE / RISKLIMITS; RUN;

PROC PHREG DATA=SHIM.DIS_1_CVA_S_7_B0; CLASS CASE; MODEL DIFFMO_2*CVA(0)=TTX_CHOL / RISKLIMITS; RUN;

PROC PHREG DATA=SHIM.DIS_1_CVA_S_7_B0; CLASS CASE; MODEL DIFFMO_2*CVA(0)=TTX_COA / RISKLIMITS; RUN;

PROC PHREG DATA=SHIM.DIS_1_CVA_S_7_B0; CLASS CASE; MODEL DIFFMO_2*CVA(0)=TTX_PLT / RISKLIMITS; RUN;

PROC PHREG DATA=SHIM.DIS_1_CVA_S_7_B0; CLASS CASE; MODEL DIFFMO_2*CVA(0)=TTX_ANT / RISKLIMITS; RUN;

PROC PHREG DATA=SHIM.DIS_1_CVA_S_7_B0; CLASS CASE; MODEL DIFFMO_2*CVA(0)=DDX_HTN / RISKLIMITS; RUN;

PROC PHREG DATA=SHIM.DIS_1_CVA_S_7_B0; CLASS CASE; MODEL DIFFMO_2*CVA(0)=DDX_LIV / RISKLIMITS; RUN;

PROC PHREG DATA=SHIM.DIS_1_CVA_S_7_B0; CLASS CASE; MODEL DIFFMO_2*CVA(0)=DDX_CKD / RISKLIMITS; RUN;

PROC PHREG DATA=SHIM.DIS_1_CVA_S_7_B0; CLASS CASE; MODEL DIFFMO_2*CVA(0)=DDX_COPD / RISKLIMITS; RUN;

PROC PHREG DATA=SHIM.DIS_1_CVA_S_7_B0; CLASS CASE; MODEL DIFFMO_2*CVA(0)=DDX_ASTH / RISKLIMITS; RUN;

PROC PHREG DATA=SHIM.DIS_1_CVA_S_7_B0; CLASS CASE; MODEL DIFFMO_2*CVA(0)=DDX_CAN / RISKLIMITS; RUN;

PROC PHREG DATA=SHIM.DIS_1_CVA_S_7_B0; CLASS CASE; MODEL DIFFMO_2*CVA(0)=CITY / RISKLIMITS; RUN;

PROC PHREG DATA=SHIM.DIS_1_CVA_S_7_B0; CLASS CASE CHA_NEW(REF='1'); MODEL DIFFMO_2*CVA(0)=CHA_NEW / RISKLIMITS; RUN;

PROC PHREG DATA=SHIM.DIS_1_CVA_S_7_B0; CLASS CASE; MODEL DIFFMO_2*CVA(0)=INSUP / RISKLIMITS; RUN;

TITLE '====================== IHD ========================';

PROC PHREG DATA=SHIM.DIS_1_IHD_S_7_B0; CLASS CASE; MODEL DIFFMO_2*IHD(0)=CASE / RISKLIMITS; RUN;

PROC PHREG DATA=SHIM.DIS_1_IHD_S_7_B0; CLASS CASE; MODEL DIFFMO_2*IHD(0)=TTX_CHOL / RISKLIMITS; RUN;

PROC PHREG DATA=SHIM.DIS_1_IHD_S_7_B0; CLASS CASE; MODEL DIFFMO_2*IHD(0)=TTX_COA / RISKLIMITS; RUN;

PROC PHREG DATA=SHIM.DIS_1_IHD_S_7_B0; CLASS CASE; MODEL DIFFMO_2*IHD(0)=TTX_PLT / RISKLIMITS; RUN;

PROC PHREG DATA=SHIM.DIS_1_IHD_S_7_B0; CLASS CASE; MODEL DIFFMO_2*IHD(0)=TTX_ANT / RISKLIMITS; RUN;

PROC PHREG DATA=SHIM.DIS_1_IHD_S_7_B0; CLASS CASE; MODEL DIFFMO_2*IHD(0)=DDX_HTN / RISKLIMITS; RUN;

PROC PHREG DATA=SHIM.DIS_1_IHD_S_7_B0; CLASS CASE; MODEL DIFFMO_2*IHD(0)=DDX_LIV / RISKLIMITS; RUN;

PROC PHREG DATA=SHIM.DIS_1_IHD_S_7_B0; CLASS CASE; MODEL DIFFMO_2*IHD(0)=DDX_CKD / RISKLIMITS; RUN;

PROC PHREG DATA=SHIM.DIS_1_IHD_S_7_B0; CLASS CASE; MODEL DIFFMO_2*IHD(0)=DDX_COPD / RISKLIMITS; RUN;

PROC PHREG DATA=SHIM.DIS_1_IHD_S_7_B0; CLASS CASE; MODEL DIFFMO_2*IHD(0)=DDX_ASTH / RISKLIMITS; RUN;

PROC PHREG DATA=SHIM.DIS_1_IHD_S_7_B0; CLASS CASE; MODEL DIFFMO_2*IHD(0)=DDX_CAN / RISKLIMITS; RUN;

PROC PHREG DATA=SHIM.DIS_1_IHD_S_7_B0; CLASS CASE; MODEL DIFFMO_2*IHD(0)=CITY / RISKLIMITS; RUN;

PROC PHREG DATA=SHIM.DIS_1_IHD_S_7_B0; CLASS CASE CHA_NEW(REF='1'); MODEL DIFFMO_2*IHD(0)=CHA_NEW / RISKLIMITS; RUN;

PROC PHREG DATA=SHIM.DIS_1_IHD_S_7_B0; CLASS CASE; MODEL DIFFMO_2*IHD(0)=INSUP / RISKLIMITS; RUN;

TITLE '====================== DM =======================';

PROC PHREG DATA=SHIM.DIS_1_DM_S_7_B0; CLASS CASE; MODEL DIFFMO_2*DM(0)=CASE / RISKLIMITS; RUN;

PROC PHREG DATA=SHIM.DIS_1_DM_S_7_B0; CLASS CASE; MODEL DIFFMO_2*DM(0)=TTX_CHOL / RISKLIMITS; RUN;

PROC PHREG DATA=SHIM.DIS_1_DM_S_7_B0; CLASS CASE; MODEL DIFFMO_2*DM(0)=TTX_COA / RISKLIMITS; RUN;

PROC PHREG DATA=SHIM.DIS_1_DM_S_7_B0; CLASS CASE; MODEL DIFFMO_2*DM(0)=TTX_PLT / RISKLIMITS; RUN;

PROC PHREG DATA=SHIM.DIS_1_DM_S_7_B0; CLASS CASE; MODEL DIFFMO_2*DM(0)=TTX_ANT / RISKLIMITS; RUN;

PROC PHREG DATA=SHIM.DIS_1_DM_S_7_B0; CLASS CASE; MODEL DIFFMO_2*DM(0)=DDX_HTN / RISKLIMITS; RUN;

PROC PHREG DATA=SHIM.DIS_1_DM_S_7_B0; CLASS CASE; MODEL DIFFMO_2*DM(0)=DDX_LIV / RISKLIMITS; RUN;

PROC PHREG DATA=SHIM.DIS_1_DM_S_7_B0; CLASS CASE; MODEL DIFFMO_2*DM(0)=DDX_CKD / RISKLIMITS; RUN;

PROC PHREG DATA=SHIM.DIS_1_DM_S_7_B0; CLASS CASE; MODEL DIFFMO_2*DM(0)=DDX_COPD / RISKLIMITS; RUN;

PROC PHREG DATA=SHIM.DIS_1_DM_S_7_B0; CLASS CASE; MODEL DIFFMO_2*DM(0)=DDX_ASTH / RISKLIMITS; RUN;

PROC PHREG DATA=SHIM.DIS_1_DM_S_7_B0; CLASS CASE; MODEL DIFFMO_2*DM(0)=DDX_CAN / RISKLIMITS; RUN;

PROC PHREG DATA=SHIM.DIS_1_DM_S_7_B0; CLASS CASE; MODEL DIFFMO_2*DM(0)=CITY / RISKLIMITS; RUN;

PROC PHREG DATA=SHIM.DIS_1_DM_S_7_B0; CLASS CASE CHA_NEW(REF='1'); MODEL DIFFMO_2*DM(0)=CHA_NEW / RISKLIMITS; RUN;

PROC PHREG DATA=SHIM.DIS_1_DM_S_7_B0; CLASS CASE; MODEL DIFFMO_2*DM(0)=INSUP / RISKLIMITS; RUN;

TITLE '====================== MI ========================';

PROC PHREG DATA=SHIM.DIS_1_MI_S_7_B0; CLASS CASE; MODEL DIFFMO_2*MI(0)=CASE / RISKLIMITS; RUN;

PROC PHREG DATA=SHIM.DIS_1_MI_S_7_B0; CLASS CASE; MODEL DIFFMO_2*MI(0)=TTX_CHOL / RISKLIMITS; RUN;

PROC PHREG DATA=SHIM.DIS_1_MI_S_7_B0; CLASS CASE; MODEL DIFFMO_2*MI(0)=TTX_COA / RISKLIMITS; RUN;

PROC PHREG DATA=SHIM.DIS_1_MI_S_7_B0; CLASS CASE; MODEL DIFFMO_2*MI(0)=TTX_PLT / RISKLIMITS; RUN;

PROC PHREG DATA=SHIM.DIS_1_MI_S_7_B0; CLASS CASE; MODEL DIFFMO_2*MI(0)=TTX_ANT / RISKLIMITS; RUN;

PROC PHREG DATA=SHIM.DIS_1_MI_S_7_B0; CLASS CASE; MODEL DIFFMO_2*MI(0)=DDX_HTN / RISKLIMITS; RUN;

PROC PHREG DATA=SHIM.DIS_1_MI_S_7_B0; CLASS CASE; MODEL DIFFMO_2*MI(0)=DDX_LIV / RISKLIMITS; RUN;

PROC PHREG DATA=SHIM.DIS_1_MI_S_7_B0; CLASS CASE; MODEL DIFFMO_2*MI(0)=DDX_CKD / RISKLIMITS; RUN;

PROC PHREG DATA=SHIM.DIS_1_MI_S_7_B0; CLASS CASE; MODEL DIFFMO_2*MI(0)=DDX_COPD / RISKLIMITS; RUN;

PROC PHREG DATA=SHIM.DIS_1_MI_S_7_B0; CLASS CASE; MODEL DIFFMO_2*MI(0)=DDX_ASTH / RISKLIMITS; RUN;

PROC PHREG DATA=SHIM.DIS_1_MI_S_7_B0; CLASS CASE; MODEL DIFFMO_2*MI(0)=DDX_CAN / RISKLIMITS; RUN;

PROC PHREG DATA=SHIM.DIS_1_MI_S_7_B0; CLASS CASE; MODEL DIFFMO_2*MI(0)=CITY / RISKLIMITS; RUN;

PROC PHREG DATA=SHIM.DIS_1_MI_S_7_B0; CLASS CASE CHA_NEW(REF='1'); MODEL DIFFMO_2*MI(0)=CHA_NEW / RISKLIMITS; RUN;

PROC PHREG DATA=SHIM.DIS_1_MI_S_7_B0; CLASS CASE; MODEL DIFFMO_2*MI(0)=INSUP / RISKLIMITS; RUN;

TITLE '====================== ALZ ========================';

PROC PHREG DATA=SHIM.DIS_2_ALZ_S_7_B0; CLASS CASE; MODEL DIFFMO_2*ALZ(0)=CASE / RISKLIMITS; RUN;

PROC PHREG DATA=SHIM.DIS_2_ALZ_S_7_B0; CLASS CASE; MODEL DIFFMO_2*ALZ(0)=TTX_CHOL / RISKLIMITS; RUN;

PROC PHREG DATA=SHIM.DIS_2_ALZ_S_7_B0; CLASS CASE; MODEL DIFFMO_2*ALZ(0)=TTX_COA / RISKLIMITS; RUN;

PROC PHREG DATA=SHIM.DIS_2_ALZ_S_7_B0; CLASS CASE; MODEL DIFFMO_2*ALZ(0)=TTX_PLT / RISKLIMITS; RUN;

PROC PHREG DATA=SHIM.DIS_2_ALZ_S_7_B0; CLASS CASE; MODEL DIFFMO_2*ALZ(0)=TTX_ANT / RISKLIMITS; RUN;

PROC PHREG DATA=SHIM.DIS_2_ALZ_S_7_B0; CLASS CASE; MODEL DIFFMO_2*ALZ(0)=DDX_HTN / RISKLIMITS; RUN;

PROC PHREG DATA=SHIM.DIS_2_ALZ_S_7_B0; CLASS CASE; MODEL DIFFMO_2*ALZ(0)=DDX_DM / RISKLIMITS; RUN;

PROC PHREG DATA=SHIM.DIS_2_ALZ_S_7_B0; CLASS CASE; MODEL DIFFMO_2*ALZ(0)=CVD / RISKLIMITS; RUN;

PROC PHREG DATA=SHIM.DIS_2_ALZ_S_7_B0; CLASS CASE; MODEL DIFFMO_2*ALZ(0)=DDX_LIV / RISKLIMITS; RUN;

PROC PHREG DATA=SHIM.DIS_2_ALZ_S_7_B0; CLASS CASE; MODEL DIFFMO_2*ALZ(0)=DDX_CKD / RISKLIMITS; RUN;

PROC PHREG DATA=SHIM.DIS_2_ALZ_S_7_B0; CLASS CASE; MODEL DIFFMO_2*ALZ(0)=DDX_COPD / RISKLIMITS; RUN;

PROC PHREG DATA=SHIM.DIS_2_ALZ_S_7_B0; CLASS CASE; MODEL DIFFMO_2*ALZ(0)=DDX_ASTH / RISKLIMITS; RUN;

PROC PHREG DATA=SHIM.DIS_2_ALZ_S_7_B0; CLASS CASE; MODEL DIFFMO_2*ALZ(0)=DDX_CAN / RISKLIMITS; RUN;

PROC PHREG DATA=SHIM.DIS_2_ALZ_S_7_B0; CLASS CASE; MODEL DIFFMO_2*ALZ(0)=CITY / RISKLIMITS; RUN;

PROC PHREG DATA=SHIM.DIS_2_ALZ_S_7_B0; CLASS CASE CHA_NEW(REF='1'); MODEL DIFFMO_2*ALZ(0)=CHA_NEW / RISKLIMITS; RUN;

PROC PHREG DATA=SHIM.DIS_2_ALZ_S_7_B0; CLASS CASE; MODEL DIFFMO_2*ALZ(0)=INSUP / RISKLIMITS; RUN;

TITLE '====================== PKS ========================';

PROC PHREG DATA=SHIM.DIS_2_PKS_S_7_B0; CLASS CASE; MODEL DIFFMO_2*PKS(0)=CASE / RISKLIMITS; RUN;

PROC PHREG DATA=SHIM.DIS_2_PKS_S_7_B0; CLASS CASE; MODEL DIFFMO_2*PKS(0)=TTX_CHOL / RISKLIMITS; RUN;

PROC PHREG DATA=SHIM.DIS_2_PKS_S_7_B0; CLASS CASE; MODEL DIFFMO_2*PKS(0)=TTX_COA / RISKLIMITS; RUN;

PROC PHREG DATA=SHIM.DIS_2_PKS_S_7_B0; CLASS CASE; MODEL DIFFMO_2*PKS(0)=TTX_PLT / RISKLIMITS; RUN;

PROC PHREG DATA=SHIM.DIS_2_PKS_S_7_B0; CLASS CASE; MODEL DIFFMO_2*PKS(0)=TTX_ANT / RISKLIMITS; RUN;

PROC PHREG DATA=SHIM.DIS_2_PKS_S_7_B0; CLASS CASE; MODEL DIFFMO_2*PKS(0)=DDX_HTN / RISKLIMITS; RUN;

PROC PHREG DATA=SHIM.DIS_2_PKS_S_7_B0; CLASS CASE; MODEL DIFFMO_2*PKS(0)=DDX_DM / RISKLIMITS; RUN;

PROC PHREG DATA=SHIM.DIS_2_PKS_S_7_B0; CLASS CASE; MODEL DIFFMO_2*PKS(0)=CVD / RISKLIMITS; RUN;

PROC PHREG DATA=SHIM.DIS_2_PKS_S_7_B0; CLASS CASE; MODEL DIFFMO_2*PKS(0)=DDX_LIV / RISKLIMITS; RUN;

PROC PHREG DATA=SHIM.DIS_2_PKS_S_7_B0; CLASS CASE; MODEL DIFFMO_2*PKS(0)=DDX_CKD / RISKLIMITS; RUN;

PROC PHREG DATA=SHIM.DIS_2_PKS_S_7_B0; CLASS CASE; MODEL DIFFMO_2*PKS(0)=DDX_COPD / RISKLIMITS; RUN;

PROC PHREG DATA=SHIM.DIS_2_PKS_S_7_B0; CLASS CASE; MODEL DIFFMO_2*PKS(0)=DDX_ASTH / RISKLIMITS; RUN;

PROC PHREG DATA=SHIM.DIS_2_PKS_S_7_B0; CLASS CASE; MODEL DIFFMO_2*PKS(0)=DDX_CAN / RISKLIMITS; RUN;

PROC PHREG DATA=SHIM.DIS_2_PKS_S_7_B0; CLASS CASE; MODEL DIFFMO_2*PKS(0)=CITY / RISKLIMITS; RUN;

PROC PHREG DATA=SHIM.DIS_2_PKS_S_7_B0; CLASS CASE CHA_NEW(REF='1'); MODEL DIFFMO_2*PKS(0)=CHA_NEW / RISKLIMITS; RUN;

PROC PHREG DATA=SHIM.DIS_2_PKS_S_7_B0; CLASS CASE; MODEL DIFFMO_2*PKS(0)=INSUP / RISKLIMITS; RUN;

TITLE '*****************************BAGE = 1 ***************************';

TITLE '====================== CVA ========================';

PROC PHREG DATA=SHIM.DIS_1_CVA_S_7_B1; CLASS CASE; MODEL DIFFMO_2*CVA(0)=CASE / RISKLIMITS; RUN;

PROC PHREG DATA=SHIM.DIS_1_CVA_S_7_B1; CLASS CASE; MODEL DIFFMO_2*CVA(0)=TTX_CHOL / RISKLIMITS; RUN;

PROC PHREG DATA=SHIM.DIS_1_CVA_S_7_B1; CLASS CASE; MODEL DIFFMO_2*CVA(0)=TTX_COA / RISKLIMITS; RUN;

PROC PHREG DATA=SHIM.DIS_1_CVA_S_7_B1; CLASS CASE; MODEL DIFFMO_2*CVA(0)=TTX_PLT / RISKLIMITS; RUN;

PROC PHREG DATA=SHIM.DIS_1_CVA_S_7_B1; CLASS CASE; MODEL DIFFMO_2*CVA(0)=TTX_ANT / RISKLIMITS; RUN;

PROC PHREG DATA=SHIM.DIS_1_CVA_S_7_B1; CLASS CASE; MODEL DIFFMO_2*CVA(0)=DDX_HTN / RISKLIMITS; RUN;

PROC PHREG DATA=SHIM.DIS_1_CVA_S_7_B1; CLASS CASE; MODEL DIFFMO_2*CVA(0)=DDX_LIV / RISKLIMITS; RUN;

PROC PHREG DATA=SHIM.DIS_1_CVA_S_7_B1; CLASS CASE; MODEL DIFFMO_2*CVA(0)=DDX_CKD / RISKLIMITS; RUN;

PROC PHREG DATA=SHIM.DIS_1_CVA_S_7_B1; CLASS CASE; MODEL DIFFMO_2*CVA(0)=DDX_COPD / RISKLIMITS; RUN;

PROC PHREG DATA=SHIM.DIS_1_CVA_S_7_B1; CLASS CASE; MODEL DIFFMO_2*CVA(0)=DDX_ASTH / RISKLIMITS; RUN;

PROC PHREG DATA=SHIM.DIS_1_CVA_S_7_B1; CLASS CASE; MODEL DIFFMO_2*CVA(0)=DDX_CAN / RISKLIMITS; RUN;

PROC PHREG DATA=SHIM.DIS_1_CVA_S_7_B1; CLASS CASE; MODEL DIFFMO_2*CVA(0)=CITY / RISKLIMITS; RUN;

PROC PHREG DATA=SHIM.DIS_1_CVA_S_7_B1; CLASS CASE CHA_NEW(REF='1'); MODEL DIFFMO_2*CVA(0)=CHA_NEW / RISKLIMITS; RUN;

PROC PHREG DATA=SHIM.DIS_1_CVA_S_7_B1; CLASS CASE; MODEL DIFFMO_2*CVA(0)=INSUP / RISKLIMITS; RUN;

TITLE '====================== IHD ========================';

PROC PHREG DATA=SHIM.DIS_1_IHD_S_7_B1; CLASS CASE; MODEL DIFFMO_2*IHD(0)=CASE / RISKLIMITS; RUN;

PROC PHREG DATA=SHIM.DIS_1_IHD_S_7_B1; CLASS CASE; MODEL DIFFMO_2*IHD(0)=TTX_CHOL / RISKLIMITS; RUN;

PROC PHREG DATA=SHIM.DIS_1_IHD_S_7_B1; CLASS CASE; MODEL DIFFMO_2*IHD(0)=TTX_COA / RISKLIMITS; RUN;

PROC PHREG DATA=SHIM.DIS_1_IHD_S_7_B1; CLASS CASE; MODEL DIFFMO_2*IHD(0)=TTX_PLT / RISKLIMITS; RUN;

PROC PHREG DATA=SHIM.DIS_1_IHD_S_7_B1; CLASS CASE; MODEL DIFFMO_2*IHD(0)=TTX_ANT / RISKLIMITS; RUN;

PROC PHREG DATA=SHIM.DIS_1_IHD_S_7_B1; CLASS CASE; MODEL DIFFMO_2*IHD(0)=DDX_HTN / RISKLIMITS; RUN;

PROC PHREG DATA=SHIM.DIS_1_IHD_S_7_B1; CLASS CASE; MODEL DIFFMO_2*IHD(0)=DDX_LIV / RISKLIMITS; RUN;

PROC PHREG DATA=SHIM.DIS_1_IHD_S_7_B1; CLASS CASE; MODEL DIFFMO_2*IHD(0)=DDX_CKD / RISKLIMITS; RUN;

PROC PHREG DATA=SHIM.DIS_1_IHD_S_7_B1; CLASS CASE; MODEL DIFFMO_2*IHD(0)=DDX_COPD / RISKLIMITS; RUN;

PROC PHREG DATA=SHIM.DIS_1_IHD_S_7_B1; CLASS CASE; MODEL DIFFMO_2*IHD(0)=DDX_ASTH / RISKLIMITS; RUN;

PROC PHREG DATA=SHIM.DIS_1_IHD_S_7_B1; CLASS CASE; MODEL DIFFMO_2*IHD(0)=DDX_CAN / RISKLIMITS; RUN;

PROC PHREG DATA=SHIM.DIS_1_IHD_S_7_B1; CLASS CASE; MODEL DIFFMO_2*IHD(0)=CITY / RISKLIMITS; RUN;

PROC PHREG DATA=SHIM.DIS_1_IHD_S_7_B1; CLASS CASE CHA_NEW(REF='1'); MODEL DIFFMO_2*IHD(0)=CHA_NEW / RISKLIMITS; RUN;

PROC PHREG DATA=SHIM.DIS_1_IHD_S_7_B1; CLASS CASE; MODEL DIFFMO_2*IHD(0)=INSUP / RISKLIMITS; RUN;

TITLE '====================== DM ========================';

PROC PHREG DATA=SHIM.DIS_1_DM_S_7_B1; CLASS CASE; MODEL DIFFMO_2*DM(0)=CASE / RISKLIMITS; RUN;

PROC PHREG DATA=SHIM.DIS_1_DM_S_7_B1; CLASS CASE; MODEL DIFFMO_2*DM(0)=TTX_CHOL / RISKLIMITS; RUN;

PROC PHREG DATA=SHIM.DIS_1_DM_S_7_B1; CLASS CASE; MODEL DIFFMO_2*DM(0)=TTX_COA / RISKLIMITS; RUN;

PROC PHREG DATA=SHIM.DIS_1_DM_S_7_B1; CLASS CASE; MODEL DIFFMO_2*DM(0)=TTX_PLT / RISKLIMITS; RUN;

PROC PHREG DATA=SHIM.DIS_1_DM_S_7_B1; CLASS CASE; MODEL DIFFMO_2*DM(0)=TTX_ANT / RISKLIMITS; RUN;

PROC PHREG DATA=SHIM.DIS_1_DM_S_7_B1; CLASS CASE; MODEL DIFFMO_2*DM(0)=DDX_HTN / RISKLIMITS; RUN;

PROC PHREG DATA=SHIM.DIS_1_DM_S_7_B1; CLASS CASE; MODEL DIFFMO_2*DM(0)=DDX_LIV / RISKLIMITS; RUN;

PROC PHREG DATA=SHIM.DIS_1_DM_S_7_B1; CLASS CASE; MODEL DIFFMO_2*DM(0)=DDX_CKD / RISKLIMITS; RUN;

PROC PHREG DATA=SHIM.DIS_1_DM_S_7_B1; CLASS CASE; MODEL DIFFMO_2*DM(0)=DDX_COPD / RISKLIMITS; RUN;

PROC PHREG DATA=SHIM.DIS_1_DM_S_7_B1; CLASS CASE; MODEL DIFFMO_2*DM(0)=DDX_ASTH / RISKLIMITS; RUN;

PROC PHREG DATA=SHIM.DIS_1_DM_S_7_B1; CLASS CASE; MODEL DIFFMO_2*DM(0)=DDX_CAN / RISKLIMITS; RUN;

PROC PHREG DATA=SHIM.DIS_1_DM_S_7_B1; CLASS CASE; MODEL DIFFMO_2*DM(0)=CITY / RISKLIMITS; RUN;

PROC PHREG DATA=SHIM.DIS_1_DM_S_7_B1; CLASS CASE CHA_NEW(REF='1'); MODEL DIFFMO_2*DM(0)=CHA_NEW / RISKLIMITS; RUN;

PROC PHREG DATA=SHIM.DIS_1_DM_S_7_B1; CLASS CASE; MODEL DIFFMO_2*DM(0)=INSUP / RISKLIMITS; RUN;

TITLE '====================== MI ========================';

PROC PHREG DATA=SHIM.DIS_1_MI_S_7_B1; CLASS CASE; MODEL DIFFMO_2*MI(0)=CASE / RISKLIMITS; RUN;

PROC PHREG DATA=SHIM.DIS_1_MI_S_7_B1; CLASS CASE; MODEL DIFFMO_2*MI(0)=TTX_CHOL / RISKLIMITS; RUN;

PROC PHREG DATA=SHIM.DIS_1_MI_S_7_B1; CLASS CASE; MODEL DIFFMO_2*MI(0)=TTX_COA / RISKLIMITS; RUN;

PROC PHREG DATA=SHIM.DIS_1_MI_S_7_B1; CLASS CASE; MODEL DIFFMO_2*MI(0)=TTX_PLT / RISKLIMITS; RUN;

PROC PHREG DATA=SHIM.DIS_1_MI_S_7_B1; CLASS CASE; MODEL DIFFMO_2*MI(0)=TTX_ANT / RISKLIMITS; RUN;

PROC PHREG DATA=SHIM.DIS_1_MI_S_7_B1; CLASS CASE; MODEL DIFFMO_2*MI(0)=DDX_HTN / RISKLIMITS; RUN;

PROC PHREG DATA=SHIM.DIS_1_MI_S_7_B1; CLASS CASE; MODEL DIFFMO_2*MI(0)=DDX_LIV / RISKLIMITS; RUN;

PROC PHREG DATA=SHIM.DIS_1_MI_S_7_B1; CLASS CASE; MODEL DIFFMO_2*MI(0)=DDX_CKD / RISKLIMITS; RUN;

PROC PHREG DATA=SHIM.DIS_1_MI_S_7_B1; CLASS CASE; MODEL DIFFMO_2*MI(0)=DDX_COPD / RISKLIMITS; RUN;

PROC PHREG DATA=SHIM.DIS_1_MI_S_7_B1; CLASS CASE; MODEL DIFFMO_2*MI(0)=DDX_ASTH / RISKLIMITS; RUN;

PROC PHREG DATA=SHIM.DIS_1_MI_S_7_B1; CLASS CASE; MODEL DIFFMO_2*MI(0)=DDX_CAN / RISKLIMITS; RUN;

PROC PHREG DATA=SHIM.DIS_1_MI_S_7_B1; CLASS CASE; MODEL DIFFMO_2*MI(0)=CITY / RISKLIMITS; RUN;

PROC PHREG DATA=SHIM.DIS_1_MI_S_7_B1; CLASS CASE CHA_NEW(REF='1'); MODEL DIFFMO_2*MI(0)=CHA_NEW / RISKLIMITS; RUN;

PROC PHREG DATA=SHIM.DIS_1_MI_S_7_B1; CLASS CASE; MODEL DIFFMO_2*MI(0)=INSUP / RISKLIMITS; RUN;

TITLE '====================== ALZ ========================';

PROC PHREG DATA=SHIM.DIS_2_ALZ_S_7_B1; CLASS CASE; MODEL DIFFMO_2*ALZ(0)=CASE / RISKLIMITS; RUN;

PROC PHREG DATA=SHIM.DIS_2_ALZ_S_7_B1; CLASS CASE; MODEL DIFFMO_2*ALZ(0)=TTX_CHOL / RISKLIMITS; RUN;

PROC PHREG DATA=SHIM.DIS_2_ALZ_S_7_B1; CLASS CASE; MODEL DIFFMO_2*ALZ(0)=TTX_COA / RISKLIMITS; RUN;

PROC PHREG DATA=SHIM.DIS_2_ALZ_S_7_B1; CLASS CASE; MODEL DIFFMO_2*ALZ(0)=TTX_PLT / RISKLIMITS; RUN;

PROC PHREG DATA=SHIM.DIS_2_ALZ_S_7_B1; CLASS CASE; MODEL DIFFMO_2*ALZ(0)=TTX_ANT / RISKLIMITS; RUN;

PROC PHREG DATA=SHIM.DIS_2_ALZ_S_7_B1; CLASS CASE; MODEL DIFFMO_2*ALZ(0)=DDX_HTN / RISKLIMITS; RUN;

PROC PHREG DATA=SHIM.DIS_2_ALZ_S_7_B1; CLASS CASE; MODEL DIFFMO_2*ALZ(0)=DDX_DM / RISKLIMITS; RUN;

PROC PHREG DATA=SHIM.DIS_2_ALZ_S_7_B1; CLASS CASE; MODEL DIFFMO_2*ALZ(0)=CVD / RISKLIMITS; RUN;

PROC PHREG DATA=SHIM.DIS_2_ALZ_S_7_B1; CLASS CASE; MODEL DIFFMO_2*ALZ(0)=DDX_LIV / RISKLIMITS; RUN;

PROC PHREG DATA=SHIM.DIS_2_ALZ_S_7_B1; CLASS CASE; MODEL DIFFMO_2*ALZ(0)=DDX_CKD / RISKLIMITS; RUN;

PROC PHREG DATA=SHIM.DIS_2_ALZ_S_7_B1; CLASS CASE; MODEL DIFFMO_2*ALZ(0)=DDX_COPD / RISKLIMITS; RUN;

PROC PHREG DATA=SHIM.DIS_2_ALZ_S_7_B1; CLASS CASE; MODEL DIFFMO_2*ALZ(0)=DDX_ASTH / RISKLIMITS; RUN;

PROC PHREG DATA=SHIM.DIS_2_ALZ_S_7_B1; CLASS CASE; MODEL DIFFMO_2*ALZ(0)=DDX_CAN / RISKLIMITS; RUN;

PROC PHREG DATA=SHIM.DIS_2_ALZ_S_7_B1; CLASS CASE; MODEL DIFFMO_2*ALZ(0)=CITY / RISKLIMITS; RUN;

PROC PHREG DATA=SHIM.DIS_2_ALZ_S_7_B1; CLASS CASE CHA_NEW(REF='1'); MODEL DIFFMO_2*ALZ(0)=CHA_NEW / RISKLIMITS; RUN;

PROC PHREG DATA=SHIM.DIS_2_ALZ_S_7_B1; CLASS CASE; MODEL DIFFMO_2*ALZ(0)=INSUP / RISKLIMITS; RUN;

TITLE '====================== PKS ========================';

PROC PHREG DATA=SHIM.DIS_2_PKS_S_7_B1; CLASS CASE; MODEL DIFFMO_2*PKS(0)=CASE / RISKLIMITS; RUN;

PROC PHREG DATA=SHIM.DIS_2_PKS_S_7_B1; CLASS CASE; MODEL DIFFMO_2*PKS(0)=TTX_CHOL / RISKLIMITS; RUN;

PROC PHREG DATA=SHIM.DIS_2_PKS_S_7_B1; CLASS CASE; MODEL DIFFMO_2*PKS(0)=TTX_COA / RISKLIMITS; RUN;

PROC PHREG DATA=SHIM.DIS_2_PKS_S_7_B1; CLASS CASE; MODEL DIFFMO_2*PKS(0)=TTX_PLT / RISKLIMITS; RUN;

PROC PHREG DATA=SHIM.DIS_2_PKS_S_7_B1; CLASS CASE; MODEL DIFFMO_2*PKS(0)=TTX_ANT / RISKLIMITS; RUN;

PROC PHREG DATA=SHIM.DIS_2_PKS_S_7_B1; CLASS CASE; MODEL DIFFMO_2*PKS(0)=DDX_HTN / RISKLIMITS; RUN;

PROC PHREG DATA=SHIM.DIS_2_PKS_S_7_B1; CLASS CASE; MODEL DIFFMO_2*PKS(0)=DDX_DM / RISKLIMITS; RUN;

PROC PHREG DATA=SHIM.DIS_2_PKS_S_7_B1; CLASS CASE; MODEL DIFFMO_2*PKS(0)=CVD / RISKLIMITS; RUN;

PROC PHREG DATA=SHIM.DIS_2_PKS_S_7_B1; CLASS CASE; MODEL DIFFMO_2*PKS(0)=DDX_LIV / RISKLIMITS; RUN;

PROC PHREG DATA=SHIM.DIS_2_PKS_S_7_B1; CLASS CASE; MODEL DIFFMO_2*PKS(0)=DDX_CKD / RISKLIMITS; RUN;

PROC PHREG DATA=SHIM.DIS_2_PKS_S_7_B1; CLASS CASE; MODEL DIFFMO_2*PKS(0)=DDX_COPD / RISKLIMITS; RUN;

PROC PHREG DATA=SHIM.DIS_2_PKS_S_7_B1; CLASS CASE; MODEL DIFFMO_2*PKS(0)=DDX_ASTH / RISKLIMITS; RUN;

PROC PHREG DATA=SHIM.DIS_2_PKS_S_7_B1; CLASS CASE; MODEL DIFFMO_2*PKS(0)=DDX_CAN / RISKLIMITS; RUN;

PROC PHREG DATA=SHIM.DIS_2_PKS_S_7_B1; CLASS CASE; MODEL DIFFMO_2*PKS(0)=CITY / RISKLIMITS; RUN;

PROC PHREG DATA=SHIM.DIS_2_PKS_S_7_B1; CLASS CASE CHA_NEW(REF='1'); MODEL DIFFMO_2*PKS(0)=CHA_NEW / RISKLIMITS; RUN;

PROC PHREG DATA=SHIM.DIS_2_PKS_S_7_B1; CLASS CASE; MODEL DIFFMO_2*PKS(0)=INSUP / RISKLIMITS; RUN;

*/
